# A Practical Guide to Visualization and Statistical Analysis of *R. solanacearum* Infection Data Using R

**DOI:** 10.3389/fpls.2017.00623

**Published:** 2017-04-24

**Authors:** Niklas Schandry

**Affiliations:** Department of General Genetics, Centre for the Molecular Biology of Plants (ZMBP), University of TübingenTübingen, Germany

**Keywords:** *Ralstonia solanacearum*, data analysis, linear mixed effects model, survival analysis, regression analysis, non-parametric testing, phytopathology, code:R

## Abstract

This paper describes and summarizes approaches for visualization and statistical analysis using data from *Ralstonia solanacearum* infection experiments based on methods and concepts that are broadly applicable. Members of the *R. solanacearum* species complex cause bacterial wilt disease. Bacterial wilt is a lethal plant disease and has been studied for over 100 years. During this time various methods to quantify disease and different ways to analyze the generated data have been employed. Here, I aim to provide a general background on three distinct and commonly used measures of disease: the area under the disease progression curve, longitudinal recordings of disease severity and host survival. I will discuss how one can proceed with visualization, statistical analysis, and interpretation using different datasets while revisiting the general concepts of statistical analysis. Datasets and R code to perform all analyses discussed here are included in the supplement.

## Introduction

The *Ralstonia solanacearum* species complex (Rssc) is the causal agent of bacterial wilt disease and is one of the most devastating bacterial plant pathogens known (Mansfield et al., 2012). Endemic in warmer climates and listed as a quarantine organism in other regions, the Rssc exhibits a remarkably broad host range. Over 200 plant families have been reported as hosts for *R. solanacearum* in the literature (Genin and Denny, 2012). *R. solanacearum* is a soil-borne plant pathogen, and natural infections usually start with an invasion of the root and subsequent colonization of xylem vessels, also in the aerial parts of the plant. Most compatible interactions between *R. solanacearum* and a host end with the plant dying of bacterial wilt disease. As for most xylem inhabiting plant pathogens, genetic resistances are scarce (Huet, 2014; Bae et al., 2015).

Research into how these pathogen species, initially described as “*Bacillus solanacearum*” (Smith, 1896), cause disease has been carried out for over a century and many aspects of bacterial wilt disease are the subjects of active, experimental research. Experiments with *R. solanacearum* and their plant hosts are usually aimed at assessing the performance of different strains or strain genotypes on a single plant species. Alternatively, to identify genetic resources of resistance in plant genotypes, a single bacterial strain can be assayed across multiple plant genotypes. To assess the performance of a bacterial strain on a certain plant, the plant is infected using a pure culture. After infection, the plant is monitored for the development of bacterial wilt disease and the disease development is scored in regular time intervals. However, after conducting an experiment one is faced with a new challenge: analyzing the collected data.

Here, I will discuss and compare a range of statistical methods which have been used in recent *R. solanacearum* literature. These are either based on an analysis of the area under the disease progression curve (AUDPC, used for example in Strange and Scott, 2005; Wydra and Beri, 2006; Hadiwiyono et al., 2007; N’Guessan et al., 2012; Lebeau et al., 2013; Meng et al., 2015), the relationship of disease index and time in a linear framework (Franks et al., 2008; Colburn-Clifford et al., 2010; Plener et al., 2010; Jacobs et al., 2012; Monteiro et al., 2012; Le Roux et al., 2015; Ailloud et al., 2016; Mori et al., 2016) or survival analysis (e.g., Plener et al., 2010; Remigi et al., 2011; Poueymiro et al., 2014; Wang et al., 2015) in the context of a biological dataset. I will present and discuss the outputs of different analyses performed on one dataset to provide some orientation regarding the interpretation and applicability of specific approaches.

All analysis presented here can be repeated and explored in more detail using the *R* scripts and corresponding datasets, found in the Supplementary Files of this publication. The scripts are provided in *rmarkdown* format that aims to provide reproducibility in data analysis. In *rmarkdown* this goal is achieved by generating a combination of free text, verbatim analysis code and the code output, which can be plots, tables or any other output produced by the *R* code in a single document (Allaire et al., 2015). In the Supplementary Material, rmarkdown files, the datasets analyzed and the output (in html format) are provided.

This paper provides an entry-point into statistical analysis, using disease assay data from *R. solanacearum* infection experiments as examples, with an emphasis on reproducible statistical reporting and including some guidelines on the interpretation of model coefficients in the context of plant disease. I will limit myself to analysis which can be derived from the one definition of the Disease Index (see Material and Methods), however, different definitions have been used in the literature. For example, Katawczik et al. (2016) use a weighted measurement of disease incidence and perform an analysis in a generalized linear framework.

The analysis performed here on the disease index over time assumes a linear relationship, but for some datasets use of logistic models (as for example used here to study *Verticilium* wilt Ben et al., 2013) may be more appropriate. Logistic regression is a complex approach and interpretation of the model coefficients can be challenging. Therefore, logistic regression models will here only be discussed in the context of survival analysis but not to assess differences in the relationship of disease index and time directly. The core concepts and the rationale are broadly applicable and described in a manner accessible to non-mathematicians.

## Materials and Methods

### Material

#### Recording Data and Quantifying Disease

The “Disease Index” (DI) is a commonly used measure to quantify disease phenotypes. However, the DI is not formally defined, and different definitions are used in the literature. I will use a commonly used definition of the DI, where wilting symptoms are quantified regularly over a defined time, based on a scale of 0–4. Here, one whole number corresponds to a 25% interval of total wilted leaves per plant.

Formally, this DI is defined as:

$$\begin{array}{c} \mathrm{DI}=\frac{w}{t}*4 \end{array}$$

where w is the number of wilted leaves, and t is the number of total leaves of a single plant. This is multiplied by 4 and rounded with a precision of either 1 or 0.5. One DI is recorded per individual and time point. Independently of the infection method used and the precise research question, one score is commonly recorded per plant per day. Recovery from the infection (a decrease in DI from one time point to the next) is not typical for *R. solanacearum* infections and therefore not considered in modeling. In experimental inoculations, the total observation time is typically between 10 and 30 days.

Depending on the scientific question one aims to answer in the analysis, the DI can either be used directly as a response variable, or a more suitable response variable can be derived from it.

#### Response Variables

**FIGURE 1:**
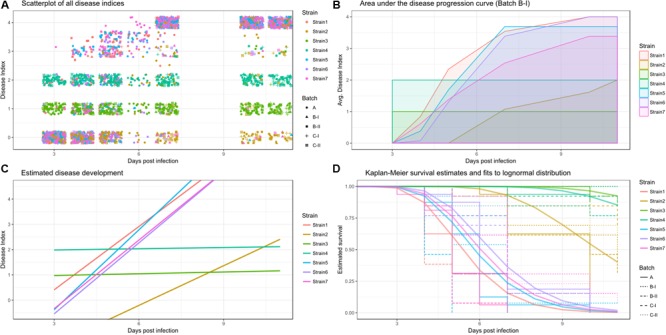
**Raw and processed data colored by strain. (A)** Overview of the raw disease index recordings, individual data-points shaped by replicate. **(B)** Disease progression curve, averaged across all plants for an individual batch. **(C)** Estimates of a linear mixed effects model of disease development per strain (averaged across all replicates and individuals). **(D)** Kaplan-Meier survival estimates are shown in light colors with a stepwise display, with different linetypes indicating experimental replicates. Solid, thicker lines show the survival regression fit to a lognormal distribution across all replicates. The event of interest was defined as a disease index of 2.5.

This section will explain how the disease index and time can be used to analyze different aspects of disease. **Figure 1** shows an example disease index data set (**Figure 1A**) analyzed using the three different methods (**Figures 1B–D**). These three methods are the area under the disease progression curve, analysis of disease indexes over time and survival analysis. Each of these measures has specific biological implications, outlined in the subsequent paragraphs and each analysis based on these different responses should be interpreted differently, to be able to make conclusions on the underlying biological phenomena. Conducting multiple analyses using distinct measures and comparing their respective outcomes can be a great aid in arriving at biologically meaningful conclusions.

The three response variables, and respective analyses are outlined below.

(I) Area under the disease progression curve (AUDPC): AUDPC is a historically used and well-established measure of disease in plant pathology and bacterial wilt research (Strange and Scott, 2005; Wydra and Beri, 2006; Hadiwiyono et al., 2007; N’Guessan et al., 2012; Lebeau et al., 2013; Meng et al., 2015). Briefly, the disease progression is drawn, and then the area defined by that curve and the x-axis is calculated. To draw the disease progression curve, one takes the mean DI for each time point, and then connects the dots. Once the disease progression curve was drawn, the area under that curve can be calculated. As the AUDPC will increase with time, reasonable comparisons of AUDPC can only be made for experiments of the same observation time (**Figure 1B**). As the AUDPC provides a linked measure of disease incidence and time, one can consider AUDPC a measure of disease severity, meaning it can be used to summarize the disease progression over time in a single value.

(II) Disease indices over time: in this approach, the DI is scored daily, based on the relative number of wilted leaves per plant, as described above. Subsequently, the DI is used as a response variable for the analysis. Treatment and treatment in relation to the time that has passed since inoculation are used as predictors (**Figure 1C**) (Franks et al., 2008; Colburn-Clifford et al., 2010; Plener et al., 2010; Jacobs et al., 2012; Monteiro et al., 2012; Ailloud et al., 2016; Mori et al., 2016). An analysis of disease indices over time usually aims to describe the disease progression curve itself as opposed to an analysis of the area encompassed by that curve outlined above. Two different approaches may be taken, either one can attempt to analyze the speed of disease progression, while disease is still actively developing, this can be done in a simple linear framework. However, an alternative could be the application of logistic regression models (Ben et al., 2013), which can accommodate the overall shape of the disease progression curve. Here, I will use linear regression to analyze the speed of disease progression, and more sophisticated, generalized linear models are employed for survival analysis (see below).

(III) Survival: methods from Survival analysis aim to describe the incidence of a certain event within a population over time. In the eponymous situation, that event is “death,” and one is interested in how long it takes until a certain fraction of the population has died, for example to assess efficacy of a treatment. However, any binary event of interest, such as symptomatic vs. asymptomatic hosts or infected vs. uninfected hosts can be used.

For the purpose of survival analysis, disease severity is transformed into a binary scoring, by defining a specific DI value as the event of interest and then recording when each individual reaches this DI value (Plener et al., 2010; Remigi et al., 2011; Poueymiro et al., 2014; Wang et al., 2015). For simplicity, I will continue here using host death as the event of interest, however, in Supplementary Material IIB it is exemplified how these methods can be used to analyze symptom onset.

To be suitable for survival analysis, the disease index dataset needs to be turned into a survival dataset. In a survival dataset, if an individual dies, the day of death is recorded, together with a status indicating “dead.” If an individual survives until the end of the trial, this is recorded as status “alive,” and the last day of observation is recorded as date. Based on the number of subjects in a cohort alive at a given time point, a Kaplan-Meier survival estimate can be calculated and further, the survival over time can be fitted to a specific distribution to proceed with parametric testing (**Figure 1D**). Survival analysis provides a way to analyze *survival of populations* upon bacterial challenge. Additionally, survival analysis offers methods to estimate and compare the hazard, which is the risk of dying at a given time point, different populations are exposed to.

#### Data Tables

Statistical analysis is based on table calculations. Proper table formatting is crucial to be able to properly interface with the R framework of analysis and syntax. In the Supplementary R scripts, formatting is done within R to generate data that conform with the concepts of tidy data (Wickham, 2012).

#### R & R Packages

The R language and environment is maintained by the R foundation and available from R-project.org (R Core Team, 2014). RStudio (RStudio Team, 2015) is an integrated development environment for R, free for academic researchers.

Many of the functions used in the Supplementary scripts are not part of the R base installation. Instead, these are made available through packages and development of new packages and methods is an active area of research. R packages can be retrieved from the Comprehensive R Archive Network (CRAN).

To be able to use the Supplementary R scripts the following packages need to be installed.

For data manipulation and visualization: tidyr (Wickham, 2016c), dplyr (Wickham and Francois, 2016), ggplot2 (Wickham, 2009), modelr (Wickham, 2016a) and stringr (Wickham, 2016b) [these and other helpful packages are part of the *tidyverse* (Wickham, 2016d)].

Functions from the following packages are used for analysis: MESS (Ekstrøm, 2016), survival (Therneau and Grambsch, 2009; Therneau, 2015a), survcomp (Haibe-Kains et al., 2008; Schroder et al., 2011), rms (Harrel, 2015, 2016), coxme (Therneau, 2015b), lme4 (Bates et al., 2014), lmerTest (Kuznetsova et al., 2016), multcomp (Hothorn et al., 2008), and rcompanion (Mangiafico, 2017).

To able to work with the Supplementary “R Markdown” (.Rmd) files, the packages rmarkdown (Allaire et al., 2015) and knitr (Xie, 2014, 2015, 2016) are required. Some additional table output formatting is done using stargazer (Hlavac, 2015).

#### Datasets

The dataset used for Figure generation in this manuscript (S1) is based on a modified version of the dataset published in Wang et al. (2015) (S2) and both the modified and the original datasets are available in the supplement. Specifically, Strain3 and Strain4 were edited to exhibit an unusual behavior in terms of disease development. While this may not be a biologically probable behavior it is helpful in understanding the outcome of different analysis methods. Datasets used in S3 were published in Lowe et al. (2015) and Lowe-Power et al. (2016). The dataset from Ravelomanantsoa & Prior (S4) has not been previously published and is therefore only available in a pseudonymized form. A summary of the dataset used in the main text is given in **Table 1**.

**Table 1:** Overview of the example dataset.

Variable name	Type	Range	Comments
Strain	Categorial predictor (fixed)	1–7	Sixty eight subjects per strain
Disease index	Categorial response	0–4	One full number corresponds to 25% wilting
DPI	Continuous predictor (fixed)	3–11	Days post-infection
Batch	Categorial predictor (random)	5 Batches	Hundred and twelve subjects in Batch A, 91 in the other
Subject	Categorial predictor (random)	1–476	Each plant is assigned a unique subject identifier
AUDPC	Continuous response	0–29.75	Calculated from DPI and DI

### Analysis Methods

#### Basic Principles of Regression Analysis

Many popular types of statistical analysis are based around linear regression. As implied by the name, linear models assume that y and x exhibit a linear relationship.

When performing a linear regression analysis, one sets out to solve a linear function. A simple linear function, with one response variable (y) and one predictor (x) can be written as:

$$\begin{array}{c} y=a+b*x+e \end{array}$$

Here, “a” is the intercept, “e” is an error term and “b” is the slope. In all linear models discussed and employed here, the part that one aims to estimate and subsequently compare, corresponds to a or b. The term fitting is used to mean “optimally solving the formula for a, b and e given the values of x and y recorded.” Assuming that y is a single summary measure of disease, and x is used to denote treatments, we aim to estimate b for each individual treatment.

One can estimate the value for b and a, that best fit to the observed data. This “best fit” is optimized to exhibit the least distances to the recorded data. These distances are known as residuals. When a linear model for a single predictor (x) is solved regarding a, one essentially performs a pairwise comparison of y and x. Each distinct y is recorded paired to a single x value, and these pairs are compared. This can be understood visually if x and y are both continuous variables, one will be able to draw a line that determines y based on x. If x is not a continuous variable, but instead, for example different treatments, this becomes harder to visualize as a line, instead this can be thought of individual means that will be obtained for y depending on the value x.

**FIGURE 2:**
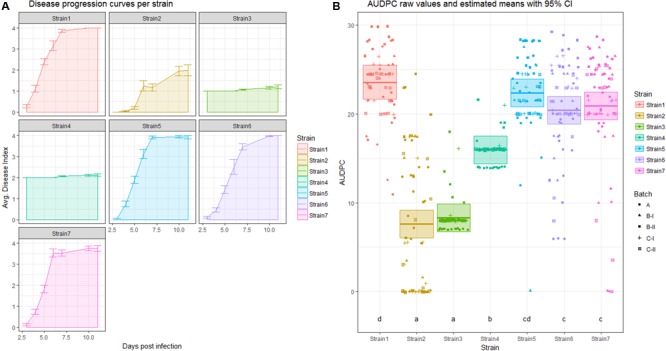
**Analysis of area under the disease progression curve (AUDPC). (A)** Disease progression curve as a line with error bars indicating standard error of the mean. The area under the curve is shaded in a lighter color according to the legend. **(B)** Means (thick horizontal lines) and 95% confidence intervals (CIs) (shaded boxes) of the AUDPC values as estimated by a linear mixed effects model (see material S1) colored by strain. Calculated areas for all individuals are plotted, with symbols indicating different replicates. Letters above the strain names indicate the significance group.

Linear models can be extended to include multiple predictor variables. This leads to an introduction of additional “x” predictors in the formula. Each predictor has its specific intercept. In **Figure 2B**, estimated “a” for each strain (corresponding to x) based on the AUDPC value (corresponding to y) is shown.

Linear regression models will attempt to estimate “a” for each known x. Statistical analysis of linear regression models can be used to assess if the estimated values for a and more importantly differences between them, are well-supported by the data. Inherently, linear models assume a normal distribution of response variables. All calculations in a linear model to estimate the true mean are performed based on this assumption. An example of this is the use of the mean to assess differences. The mean is not necessarily a useful measure for the center of a distribution, if that distribution is not normal.

Statistical analyses that assume a defined distribution and aim to estimate a certain parameter of that specific distribution, for example the mean in case of a normal distribution, are commonly referred to as “parametric.”

After model fitting the model can be explored regarding its goodness of fit, effectively assessing how close the model is to the data. Briefly, a model that has the least amount of assumptions (predictors) is preferred over one that does not provide a significantly better explanation but includes more assumptions. Model testing and selection, however, are beyond the scope of this manuscript.

In a linear model with a single predictor the estimated value of a for each level of the predictor is the mean of y across all observations for that predictor, relative to a baseline (see next section) (**Table 2**).

**Table 2:** Estimated coefficients of the analyses performed.

	Wilting analysis			Survival	
	AUDPC	Disease development		CoxME	Lognormal fit
	Mean	Slope	y-Intercept	Hazard ratio	exp(Location)
Strain1	23.6	0.84	–2.2	NA(^∗^)	5.38
Strain2	7.6	0.54	–3.5	0.07^A^	10.30
Strain3	8.3	0.05	0.9	0.01^A^	16.02
Strain4	15.9	0.04	1.9	0.01	14.46
Strain5	22.4	1.06	–3.6	0.72	5.81
Strain6	20.4	0.93	–3.4	0.44	6.40
Strain7	20.9	0.92	–3.2	0.58	6.03

*^A^Dataset violates the proportional hazards assumption; hazard ratios may not be reliable. (^∗^) Not applicable as Strain1 is the reference for ratio calculation.*

The term “estimate” often causes confusion. One reason why one has to estimate, as opposed to precisely calculate the true mean, is that one should assume that the model is incomplete and the data one has in hand is a random sample of the true population. In every experiment and in every replicate, despite best efforts to control as many factors as possible, there are things that are beyond the experimenters control. The fact that these are not controllable, does not exclude these have an influence. Hence, statistical analysis will always aim to provide a measure of certainty that what one measures is due to a change of known treatment, and not the consequence of other, uncontrolled, factors. Since it is not possible to know the influence of unknown factors, one should estimate the influence of the known treatment, with a specific level of certainty. This is related to significance testing, and is explored below in more detail.

#### Relationship between Linear Models and Analysis of Variance (ANOVA)

Analysis of variance (ANOVA) is a specific case of linear models, where the means of individual treatments, are compared to the “grand mean.” The grand mean is calculated across all treatments. Subsequently, all treatments are compared to the grand mean. In the language of linear models, this is termed effect coding of the predictor variable, as it compares the effect of each individual predictor relative to the grand mean.

Linear models allow for other types of coding of the predictor variable(s), which may be easier to interpret. I will make use of linear models, where the influence of each strain is analyzed in relation to a reference strain (treatment coding).

#### Analysis Using Linear Mixed Effect Models

Similar to classical linear models, linear mixed effect models (LMM) attempt to fit the observed data to a linear function.

In contrast to classic linear models, LMMs account for two types of predictors, also called effects, those of interest (fixed effects) and those that are not of direct interest (random effects).

One can use an LMM to analyze disease by using a measure of disease as the response variable. Depending on the experimental design and research hypothesis possible fixed effects could for example be: bacterial strain, plant genotype, soil type, competitor strains, or fertilization status.

The accompanying script and dataset provides examples, where LMMs with the AUDPC or repeated measurements of the disease index as response variables are used to assess the influence of different strains on disease development.

#### Survival Analysis

The term survival analysis unites a range of methods which aim to characterize time-to-event data across different populations. Classically the event of interest is death of an individual, or failure of a product.

##### Survival analysis: Kaplan-Meier estimates

Kaplan-Meier estimates are specific for survival analysis and are used to estimate survival times. Based on time, and amount of living individuals at a given time point, it is possible to generate a curve that describes the relative survival at any given time point. The commonly used display is the Kaplan-Meier estimate of survival (**Figure 1D**). After the first time point with an event, the number of survivors for the later time points needs to be estimated, as the population is no longer the same as in the beginning. Kaplan-Meier estimates calculated for different treatments can be compared using pairwise testing, e.g., using the Log-rank test (Altman and Bland, 1998; Bland and Altman, 1998, 2004). While the log-rank test loses power if the proportional hazards assumption is violated, it is not necessarily inappropriate. The “survival” R package allows for two variants of log-rank tests (Therneau, 2015a). The “log-rank” test is more powerful in detecting late differences while the Peto & Peto modification of the Gehan-Wilcoxon test has greater power in detecting early differences (Therneau, 2015a).

Kaplan-Meier estimates can also be analyzed using parametric regression models. The R package “survival” (Therneau, 2015a) allows for parametric analysis using four different distributions. These are the logistic distribution, lognormal distribution, Gaussian distribution, and Weibull distribution.

##### Survival analysis: Hazards

Hazards in survival analysis, describe the probability of experiencing an event at a given time point. If the hazards for the individual groups receiving different treatment can be described relative to each other by a constant these hazards are called “proportional hazards.” More visually, groups exposed to proportional hazards will usually generate non-crossing Kaplan-Meier estimates. Depending on whether the hazards are proportional different statistical methods apply.

To analyze the effect of using difference treatments, the analysis of (log transformed) hazard ratios can be used. Hazard ratios, are a ratio of the hazards of two experimental groups. If the hazard ratio is close to or exactly 1 one can assume that these hazards are equal. Hazard analysis can also be performed in a mixed model framework (Therneau, 2015b). However, comparison of hazard ratios will only yield reliable results if those hazards are proportional. In the case of non-proportional hazards other methods may be preferable for data-analysis.

The proportional hazards condition is not necessarily met in *R. solanacearum* infection studies. While this is a mere observation across multiple datasets, it may be helpful to remember what the classical application survival analysis is, namely to monitor survival across separate populations. It should be noted here, that this is a sensible approach if both populations are expected to decline similarly within the observation period. For example, a classical application for survival analysis is comparing medical or surgical intervention on patients that suffer from a medical condition. In this case, intervention is intended to prolong life.

In experimental infections with *R. solanacearum* this may not be the case. Presumably, without treatment none of the individual plants would die within the observation time. Depending on the strains used and their precise, probably not completely understood, individual interactions with the given host the disease progression may be drastically different. This may lead to a violation of the proportional hazards assumption. One should consider if, depending on the experimental design and research hypothesis, non-proportional hazards for, e.g., different strains constitutes a relevant finding.

#### Statistical Significance Testing

The choice of statistical analysis used should be made based on the underlying research hypothesis. If one is interested in the steepness of the disease progression curve, e.g., because one assumes that treatments will change the speed of disease development, linear regression of repeated DI recordings may be a useful approach. If however, one is interested in the fraction of survivors per timepoint, for example when comparing different plant cultivars in field trials, survival analysis may prove more powerful and relevant. Throughout this manuscript and the supplement I will largely employ generalized linear hypothesis testing, while adjusting for multiple comparisons using Tukey’s method, to assess statistically significant differences (Hothorn et al., 2008).

Useful and informative statistical analysis requires a clear hypothesis that describes the expected outcome. Usually the research hypothesis is that a (specific) change of treatments will lead to a (specific) change of outcome. Statistical testing attempts to lend credence to the research hypothesis via falsification of the null hypothesis. A null-hypothesis matching to the research hypothesis above would be: a change of treatment will lead to no change of outcome. One indicator whether the null hypothesis is true, is the *p*-value. For the purpose of significance testing one needs to define alpha, the significance threshold. Commonly used is an alpha of 0.05 (5%). If a *p*-value below alpha is obtained, this is taken as an indication that the null hypothesis is wrong and usually the research hypothesis is accepted instead. But, how does one get a *p*-value?

To arrive at a *p*-value, one assumes the null hypothesis to be true. Then, one estimates the true mean for each treatment. Next, one compares the estimated mean to whatever is stated in the null hypothesis. Usually the null hypothesis states either that the true mean is a specific value, or alternatively that the difference between two means obtained for two different treatments is zero. A difference in means is also called an effect.

Based on the difference in means (or the difference from a single mean to a defined value, corresponding to the mean under the null hypothesis), the degrees of freedom and test-specific calculations one arrives at a certain value, known as the test statistic. This test statistic is compared to the distribution of the test statistic. The *p*-value describes the region of the test distribution, where the obtained test statistic is located. For example, a *p*-value of 0.01 indicates that 1% of area of the test distribution are further away from the center of the test distribution than the calculated test statistic. In other words, the *p*-value describes the probability of observing an event as extreme or more extreme than the experimental outcome assuming the null hypothesis and all test specific assumptions were fulfilled.

If the obtained *p*-value is below alpha, one can reject the null hypothesis. Usually, the research hypothesis is accepted. *p*-Values are sensitive to a number of factors for example, larger sample sizes will usually decrease the calculated *p*-value, even if the observed effect is the same, because it is assumed that with more observations, an estimation of the true mean is more precise. Alpha and sample size should be defined before conducting the experiment to minimize the chance of wrongly rejecting the null hypothesis (type I error) and to maximize the chance of finding a true effect (i.e., minimizing the type II error). Power analysis is beyond the scope of this manuscript, but a starting point is the R package “pwr” (Champely, 2016).

Interpretation of the *p*-value is not necessarily easy and may be confounded by test-specific assumptions. A low *p*-value does not necessarily indicate a large difference in means; it should be understood to imply that one is unlikely to observe that outcome assuming the null hypothesis was true. Often this is not of direct interest, but instead what is more important is to know the difference in means. A significant test result for a comparatively small effect does not necessarily imply biological significance, but significance within the model used for analysis.

To combine the display of (difference in) means while providing a way to visually assess certainty of the estimate, I will make use of estimated means, and 95% confidence intervals (CIs). In such a display, if the mean of one treatment is not within the 95% CI of another, those two treatments are different assuming a significance level of 0.05 (Krzywinski and Altman, 2013).

Additionally, I will make use of “compact letter displays” to indicate groups of statistical significance. Treatments that are assigned to the same “letter” group are not significantly different. Treatments that are assigned different letters using this method, display a significance difference. For example, a treatment in group “ab” is different from one in group “c.” A treatment in group “ab” is not different from those in group “a” or “b.” However, treatments that are assigned only group “a” are different from those in group “b.” For the purpose of inferring treatment specific differences from a model that includes an interaction and covariates, the treatment coefficients are averaged.

## Results

### Choice of Response Variable and Predictors

Once a response variable has been collected, or the collected data has been transformed into a suitable response variable, the data analysis can be conducted. Already in the choice of the response variable one may be guided by a specific hypothesis. However, this hypothesis now needs to be formulated more explicitly. For a mixed model analysis one has to define which of the possible experimental factors are of interest, and which are considered not of interest. A standard example would be assessing the performance of different strains. “Strain” is then a fixed effect, as one is interested in seeing if there is a difference between the different strains. However, at the same time, one may be aware of another factor that one assumes to have influenced the outcome of the experiment. For example, individual variation or variation on pan-individual scales from seed batches or replicates may influence the outcome of the response variable through random sampling from the true population. If one is aware of such an influence, but not explicitly interested, probably because one is assuming that its influence is not systematic in its contribution to the outcome, this predictor can be treated as a “random effect.” An alternative way to distinguish fixed and random effects is by their presumed reproducibility. Strain effects should be reproducible, meaning that the differential performance of known strains should not change from replicate to replicate. I will treat replication as a random effect in most models, as it usually influences the outcome in most datasets, but is not of direct interest.

Using a response variable and one or more effects, which can be either fixed, or random, one can fit the data to a LMM, which can then be investigated regarding the influence of fixed effects on the response variable.

### Analysis of AUDPC Using an LMM

Once one has calculated the AUDPC value for each individual in the experiment, one can use these data to fit a linear mixed effects model. As the AUDPC contains information on time and the DI, the only possible predictors left are strain and replicate. In **Figure 2A**, the averaged AUDPC values are shown for each individual strain. In **Figure 2B**, the AUDPC values calculated for each individual are shown in a scatterplot, colored and separated by strain and shaped according to the replicate they belong to. Thick horizontal bars and translucently shaded boxes are used to show the estimated mean and corresponding 95% CIs, as estimated by an LMM. Below each boxplot a letter indicating significance group of each strain in an analysis of AUDPC as a response variable, with Strain as a fixed and Batch as a random effect is given. In **Figure 4A**, the pairwise difference in means is plotted, with a 95% CI. Here, if the pairwise difference with CI contains 0, this comparison is not significantly different.

**Figure 2A** also contains an example of one of the weaknesses of AUDPC analysis. As can be seen for Strain2 and Strain3, the AUDPC is not significantly different between those strains (**Figure 2**). However, one may be inclined to think that those strains behave differentially, by looking at the average disease index over time (**Figure 2**).

This highlights that AUDPC does not provide a good measure of disease development over time. It instead provides an approximate measure of disease severity over an aggregate time period.

### Analysis of the Disease Development Using LMMs

Since AUDPC based analysis appears to sometimes perform poorly although differential disease development is observed, other methods are necessary to identify differences in disease development. Disease development here is taken to mean that one is interested in the disease index in relation to time and treatment. Repeated-measure ANOVA can be used to analyze DI and time per treatment (Jacobs et al., 2013; Lowe-Power et al., 2016). However, when using repeated measure ANOVA one should be aware that the arrow of time is not considered in this analysis. In a repeated-measure ANOVA one compares strains per time point.

**FIGURE 3:**
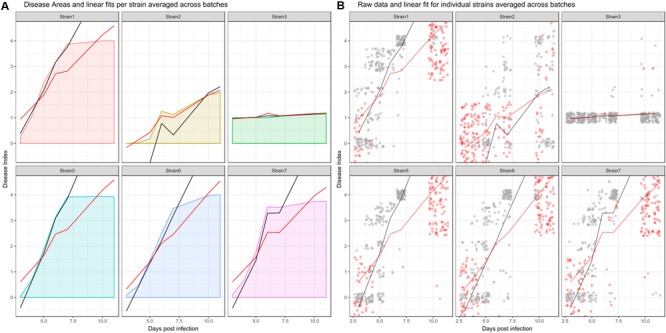
**Effect of removing recordings before disease onset and dead individuals from the dataset before model fitting when analyzing disease development. (A)** Averaged curve for individual strains, colored by strain based on all datapoints. Red and black lines show averaged linear fits. Red line: predictions from a model fit to the dataset without removing dead individuals, black line: predictions from a model fit to the dataset after removing recordings that do not reflect disease development. **(B)** Raw data across all experimental replicates. Red dots indicate observations that were removed, as they constitute either re-observations of already dead individuals or recordings that occurred before phenotypic disease development began. Gray dots indicate observations that were retained. Line coloring is the same as in the left panel. Strain4 is omitted as, similar to Strain3, disease index recordings are not affected by the above criteria.

In a LMM used to analyze disease development, it is possible to include the arrow of time. To properly account for differential disease development over time, an interaction between treatment and time is included. To analyze only the duration where disease is still developing, I suggest cleaning the dataset before fitting the data, as it may contain data that is not of interest for this specific analysis. In biological terms, a disease index of 4 reflects a plant that wilted completely. Those plants will not recover or die further, but a linear model assumes continuous relationships between y (here, the disease index) and x (here, time). To effectively analyze only those time points where disease is still developing and partially remove the categorical character of the disease index, one could simply remove all observations for an individual, after that particular plant has reached a disease index of 4. After doing so, one is effectively only using those observations to fit a model that actually reflect disease development. The effect of removing re-observations before model fitting is shown in **Figure 3** (black vs. red line). As can be seen from **Figure 3**, removing re-observations of dead individuals will in most cases lead to a fit that better reflects the average increase in DI over time, and therefore has an increased slope compared to a fit calculated using the full dataset.

To perform statistical significance testing on the linear mixed effects model, one performs pairwise comparisons of the estimated mean per strain. Such analysis can also be performed visually, for example by plotting the pairwise difference in slope between strains with the 95% CI. Unlike the *p*-value itself, an assessment of the pairwise difference with CIs actually allows for an approximation of the true difference. The absolute difference from 0 reflects the absolute difference in estimated means, and hence can be used to assess if there is a strong or weak difference relative to the other comparisons. Alternatively, one can compare the estimated means with CIs. If the mean of one treatment is not within the CI of the other those treatments are significantly different.

**FIGURE 4:**
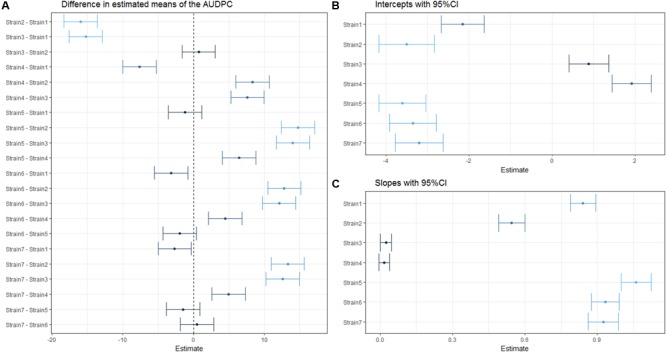
**Estimated (difference in) means of the coefficient(s) within one linear mixed effect model.** Dots indicate the estimated (difference in) means and horizontal error-bars indicate the 95% CI of the estimate. Dots and error bars are colored based on their distance from 0, where a dark color indicates a value is close to 0, while lighter colors indicate a larger difference from 0. **(A)** Pairwise difference in means using AUDPC as a response. **(B)** Estimated strain specific y-intercepts of the LMM used to analyze disease development. **(C)** Estimated strain specific slopes from the same LMM as in **(B)**.

It is evident from **Figure 4**, that while linear mixed effect models using the AUDPC as the response (**Figure 4A**) and those based on repeated measures of the disease index (**Figures 4B,C**) sometimes agree, certain comparisons differ quite drastically between the two analyses. For example, Strain2 and Strain3 are not identified as different in the AUDPC analysis (**Figure 4A**), while they exhibit a great difference in both intercept and slope estimated by an LMM used to analyze disease development (**Figures 4B,C**). This again emphasizes the impact of the choice of both response variable and predictor on the outcome of the analysis.

### Survival Analysis

#### Background and Data Type

The two types of analysis discussed previously are both based on the disease development of an individual plant. However, if a sufficiently large number of individuals are analyzed as part of an experiment, these may be viewed as a population and disease can be analyzed per population.

Before being able to start with survival analysis, the disease index scorings need to be converted to a survival table. To generate a survival table one needs to check which of the repeated observations for one individual is the first to cross a threshold that defines an event. This time point is recorded, together with a binary status indicator set to the state of “dead.” If an individual never passes the threshold, the last day of observation is recorded together with the status “alive.” Subjects that leave the study before the last day of observation can be recorded as alive on that day, known as right censoring. All variables that specify a fixed or random effect such as Strain, Plant or Batch, should be retained in the survival table. Based on such a table one can now analyze how a population survives over time, and, e.g., compare the impact of different strain treatments on the survival.

**FIGURE 5:**
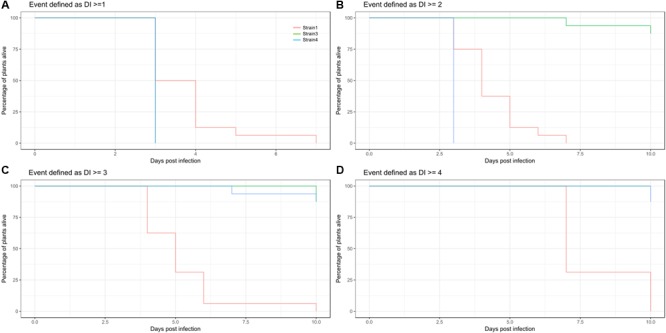
**Consequence of threshold settings on survival estimates. (A–D)** Kaplan Meier estimates of survival for Strain1 (red), Strain3 (green), and Strain4 (blue) illustrating the different outputs depending on cutoff for an event. **(A)** Estimated survival if event is defined as a disease index recording of > = 1. **(B)** Event defined as DI > = 2, **(C)** event defined as DI > = 3, **(D)** event defined as DI > = 4 (death).

The threshold of event generation has to be set by the user, and should not be done without inspecting the data first. As exemplified in **Figure 5** changed survival threshold can have dramatic impact on the resulting survival estimates.

### Survival Analysis

In the example dataset used here, different types of survival analysis can be explored. For all explorations below, the event of interest was defined as a disease index of > = 2.5. Pairwise log-rank comparisons of the Kaplan-Meier estimates can be performed, however, one should be aware that multiple pairwise comparisons are performed, which should be adjusted for (in **Table 3** Bonferroni adjustments were used). Generally, for multiple comparisons it is more advisable to perform a mixed effect analysis and subsequent comparison of estimated coefficients.

**Table 3:** Results of significance testing for the different analysis and tests.

	Wilting analysis		Survival		
Strain	AUDPC	Disease development	Log-rank *p*-value^A^	CoxME log(HR)^∗^	Lognormal fit
Strain1	A	A	–	A	A
Strain2	B	B	<1 E-10	C^A^	C
Strain3	B	C	<1 E-10	D^A^	D
Strain4	C	D	<1 E-10	D	D
Strain5	AD	A	0.112	AB	AB
Strain6	D	DE	4.5 E-06	B	B
Strain7	D	E	0.015	B	AB

*Mean coefficients are given in Table, Strains which are assigned to the same group are not significantly different. ^A^*p*-values obtained by pair-wise testing against Strain1 (adjusted for multiple comparisons); ^∗^Proportional hazard assumption violated; HR, hazard ratio.*

For this particular event, Strain2 and Strain3 significantly violate the proportional hazards relative to Strain1. Hazard ratios can however still be obtained from a Cox-Mixed effect model and can be used to compare strains, although this may in this case be unreliable.

Alternatively, the use of parametric survival regressions is a common practice. Here, the Kaplan-Meier estimate is fitted to a certain distribution (see Material and Methods). As it is a parametric fit, two parameters of the distribution are estimated for the model. One of them is the scale of the model, the other is referred to as location or shape, which is the center of the distribution. Naturally, the formula of these distributions are more complex than those of a linear model. As the dataset used here produced the best survival regression fit when the lognormal distribution was used, this distribution will be used in the subsequent example.

**FIGURE 6:**
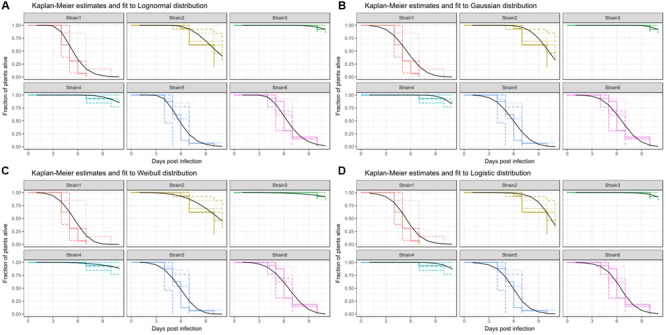
**Kaplan-Meier estimates of survival and fits produced by survival regression analysis for Strain1–Strain6.** Strain7 is not significantly different from Strain5 and therefore was omitted in this display. Different colors are used for the survival estimates of the individual strains shown. Different line-types are used for different replicates for Kaplan-Meier estimates. Regression analysis was performed across all replicates and the resulting fits are plotted with a black curve. The distribution for fitting were: **(A)** Weibull distribution, **(B)** Logistic distribution, **(C)** Gaussian distribution, and **(D)** Lognormal distribution.

A survival fit to a lognormal distribution returns two parameters, a global scale parameter (in this model 0.261), which applies to all treatments, and a treatment specific shape, or location, parameter. Usually, the shape parameter indicates the center of the distribution, e.g., for a normal distribution shape is the mean. In the case of the lognormal distribution, the shape parameter indicates the turning point of the curve, on a ln(x) scale. Therefore, exp(shape) is informative, as it gives the time-point where 50% of the population are estimated to have experienced the event (**Figure 6** and **Table 3**). It should be noted that the parametrization of survival regression depends on the distribution used for fitting and therefore the relationship between the estimated parameter and the center of the distribution may change if other distributions are used.

Different methods may result in different interpretations of the dataset. In **Table 2**, relevant estimated mean model coefficients are provided, and **Table 3** provides an overview of the inferred statistical differences, per strain, for each analysis.

## Discussion

Reproducibility is not only important in experimental procedures, but is also crucial when it comes to data analysis. Without a detailed explanation of the conducted analysis, it is nearly impossible for others to assess whether the analysis was appropriate and, perhaps more importantly, follow the reported conclusions. A common, unified nomenclature and analysis methods within a specific field of research will make cross-comparisons within that field more straight-forward, and may prove useful in achieving an over-arching scientific objective.

However, already in the field of statistics, the meaning of a certain word is not always unambiguous. Hence, *rmarkdown* (Allaire et al., 2015) facilitates the generation of standardized reports containing analysis code, code output and explanatory text. All analysis discussed here can be found in the accompanying document S1 with the original data. Using the code in S1, a complete analysis of the S1_data.csv dataset can be performed and can be easily adapted to other datasets. How this can be transferred, and how different analysis perform on other datasets is explored in Supplementary Documents S2, S3, and S4.

The three different measures of disease used here as examples each have different properties. The AUDPC measure reduces DI and time to a single value per individual, and therefore some information is lost. It is possible that distinct curves return very similar AUDPC values, as seen for Strain2 and Strain3 in **Figure 2B**. Both belong to significance group “a,” meaning that there is no statistically significant difference between those treatments regarding the AUDPC. However, if one inspects the actual shape of the curve (**Figure 2A**) one may be inclined to think that these strains are quite different in their disease development. Indeed, when the same dataset is analyzed using a linear mixed effects model based on repeated measures of the disease index, these strains exhibit a significant (α = 0.05) difference in means (**Figure 4**).

The linear mixed model for disease development employed in the analysis shown here, specifies a fixed interaction between treatment and time. By specifying an interaction between strain and time, it is assumed that the change in disease incidence over time is strain specific.

Methods from survival analysis require a survival table. If a survival table is generated based on a certain DI threshold value, the results are likely to differ depending on the value used to determine an event. As can be seen from **Figure 5**, transforming DI into event data can result in rather different Kaplan-Meier estimates for the same treatments. This will further influence all other analyses that are performed based on the survival table and its derivatives, like log-rank testing, estimation of hazard ratios or regression analysis. Therefore, the transformation from DI to survival should be done carefully and should be kept in mind when interpreting the results of the analyses.

As a guide to overall interpretation of the analysis presented here: AUDPC provides a measure in overall disease incidence. By using disease index and time as response variable and predictor, respectively, LMMs can also be used to analyze strain specific differences in disease development. Survival analysis provides a sensitive way to analyze time-to-event. Diverse events can be analyzed using survival analysis, such as disease or symptom onset or disease end. Other events, not based on the DI, could be bacterial presence in an individual, or bacterial populations crossing a certain density during colonization.

Finally, by combining different analyses and comparing their result, one may be able to gain insights into the biology. For example, in Supplementary Material 3-II, the two strains compared exhibit no overall statistically significant difference in AUDPC or disease development. However, when the disease development LMM is inspected more carefully, one finds that the intercepts do not change significantly, while the slopes are different. An increase in disease index of about 0.32 per day is estimated for the wild-type strain, while the mutant is estimated to wilt its host with a speed of about 0.24 disease indices per day. Those same strains are also significantly different when the disease onset is analyzed using survival analysis, or when disease incidence is analyzed using a repeated measure ANOVA, as presented in Lowe et al. (2015). Taken together this indicates, that: overall disease severity (approximated by AUDPC) does not change, estimated disease onset (intercepts do not change, but disease development is affected (slopes).

Combining this with the findings of the analysis of population wide disease onset using survival analysis further indicates that the fraction of the population that shows symptoms per time point is slightly lower when the mutant strain is used compared to the wild-type. This can be seen from non-parametric log-rank testing, parametric survival regression, and by the hazard ratio test. In summary it appears that the mutant strain is slower in causing symptoms, but not in overall disease severity (AUDPC).

This could be taken to indicate a delay in host colonization. As can be seen Figure 5 of Lowe et al. (2015) this indeed the case, here it is shown that the population size in root tissue differs significantly at 3 DPI but not at 6 DPI.

In summary, a combination of different statistical analysis methods can be used to understand specific differences between treatments. Once the specific differences have been identified, these can be used to develop a new research hypothesis.

## Author Contributions

The author confirms being the sole contributor of this work and approved it for publication.

## Conflict of Interest Statement

The author declares that the research was conducted in the absence of any commercial or financial relationships that could be construed as a potential conflict of interest.
