# Supplementary material for: A Practical Guide to Visualization and Statistical Analysis of R. solanacearum Infection Data Using R
Source: Front Plant Sci. 2017 Apr 24;8:623. doi: 10.3389/fpls.2017.00623 (PMC5401893; doi:10.3389/fpls.2017.00623)
Supplement: Supplementary file 1 [file Data_Sheet_1.ZIP › S1_example_dataset.html]

Approaches to analyze experimental R. solanacearum infections: Supplementary Material 1: R Code and explanations


# Approaches to analyze experimental *R. solanacearum* infections: *Supplementary Material 1:* R Code and explanations

#### *Niklas Schandry*

# General information on this document

This document is part of the supplementary material of “A practical guide to descriptive and statistical analysis of R. solanacearum infection data using R”. Here, I aim to convey how the analysis described in the main text can be performed. In this file, S1, a modified version of the Wang et al. dataset is used.

# Data Type

The disease index score is based the severity of symptoms. A symptom read-out (usually “% wilted leaves”) is turned into a numeric score. This score ranges from 0-4, and each number is used to reflect a certain interval of disease severity. The value of the disease index can never be below 0, or above 4. Therefore, the disease index is not a continuous response variable, but is ordinal. However, for simplicity and consistency with published analysis, i will treat the disease index as if it was a continuous variable. If the disease index is distributed normally this should be okay.

# Data Import, Formatting and preparation

## Specifying the data

Initially, data has to be read into R. Before data can be read, the directory containing the data needs to be specified and set. In R this is called the working directory. Anything that is read into, or exported from R will use this directory unless explicitly pointed to a different directory.

```
###Install all required packages:
install.packages(c("MESS","lme4","lmerTest","multcomp","survival","rms","coxme","stargazer","tidyverse","rcompanion"))
#biocLite("survcomp")
###Define Working Directory and set it
###Note for the Markdown version: R-Markdown cannot set the working directory
###R markdown will always use the directory the .Rmd file is located in
###In the .Rmd file this code section is not actually evaluated and only serves illustratory purpose.
wd <- c("~/My_Data/DataDirectory/")
setwd(wd)
```

Next, the dataset is specified.

```
###Name of the file to be read
table <- c("S1_dataset.csv")
```

The dataset used here (“S2\_data.csv”) is a pseudonymized and partially edited version of the dataset presented in Wang et al., 2015, kindly provided by Nemo Peeters. Strain3 and Strain4 have been edited to serve illustratory purpose.

## Reading data and formatting

The data can be read into *R* and stored in a data frame. I am calling that data frame disease\_index

```
disease_index <- as.data.frame(read.table(table, header=T,
                                          sep=";" , ###Sets the seperator of the csv file
                                          dec=","), ###Sets the decimal operator of the csv file
                                          stringsAsFactors=F) ###
```

Using str() one can see if the table was properly imported.

```
str(disease_index)
```

```
## 'data.frame':    476 obs. of  12 variables:
##  $ Strain: Factor w/ 7 levels "Strain1","Strain2",..: 1 1 1 1 1 1 1 1 1 1 ...
##  $ Plant : Factor w/ 1 level "Medicago": 1 1 1 1 1 1 1 1 1 1 ...
##  $ X3    : num  1 2 0 1 0 0 0 1 2 1 ...
##  $ X4    : num  3 3 0 3 2 0 1 3 3 1 ...
##  $ X5    : num  3.5 3.5 1 3.5 2 0 3.5 3.5 3 2 ...
##  $ X6    : num  3.5 3.5 3 3.5 3.5 0 3.5 3.5 3.5 3 ...
##  $ X7    : num  4 4 3.5 4 4 2.5 4 4 4 3.5 ...
##  $ X8    : logi  NA NA NA NA NA NA ...
##  $ X9    : logi  NA NA NA NA NA NA ...
##  $ X10   : num  4 4 4 4 4 4 4 4 4 4 ...
##  $ X11   : num  NA NA NA NA NA NA NA NA NA NA ...
##  $ Batch : Factor w/ 5 levels "A","B-I","B-II",..: 1 1 1 1 1 1 1 1 1 1 ...
```

If the table was properly imported, “Xnumber” columns should be either numeric, or logical (if they were empty). If this is the case, generation of a “subject”" column helps further analysis, which assings a unique, numeric identifier to each individual. Strain, Plant and Batch columns need to be factors. If this is not the case this can be coerced to a factor by, e.g. “di\_long\(Strain <- as.factor(di\_long\)Strain)”

```
disease_index$subject <- c(1:nrow(disease_index))
```

Now, the table of disease index recordings, needs to converted into long format for later analysis, conforming with a data structure often referred to as “tidy” (Wickham, 2008) . Long format means, that instead of having one column of each day, a new column is generated that indicates the day post infection (DPI).

```
library("tidyr")
```

```
di_long_na <- gather(disease_index, key=DPI, 
                     value=DI,
                     X3:X11, ####Specify here the days the plants were observed (this means 3 to 11)
                     na.rm=F)
```

Which will generate a long table, containing missing observations coded as NA. The table structure is as follows:

```
str(di_long_na)
```

```
## 'data.frame':    4284 obs. of  6 variables:
##  $ Strain : Factor w/ 7 levels "Strain1","Strain2",..: 1 1 1 1 1 1 1 1 1 1 ...
##  $ Plant  : Factor w/ 1 level "Medicago": 1 1 1 1 1 1 1 1 1 1 ...
##  $ Batch  : Factor w/ 5 levels "A","B-I","B-II",..: 1 1 1 1 1 1 1 1 1 1 ...
##  $ subject: int  1 2 3 4 5 6 7 8 9 10 ...
##  $ DPI    : chr  "X3" "X3" "X3" "X3" ...
##  $ DI     : num  1 2 0 1 0 0 0 1 2 1 ...
```

Next, some modifications the table structure are neccessary to make sure that all variables (columns) are in the proper format.

```
di_long_na$DI <- as.numeric(di_long_na$DI) ###Turns DI into numeric
di_long <- na.omit(di_long_na) ###Generate a table that does not contain missing observations
di_long$DPI <- na.omit( as.numeric (unlist( strsplit( as.character( di_long$DPI ), "X" ) ) ) )  ###Discard the "X" and save the number as a number (instead of a factor), it is not elegant, but it works
```

## Defining the contrasts

A crucial step that will influence all outputs of the statistical analysis is setting the “contrasts”. Contrasts specifies the reference for each of the variables. “Treatment” contrasts specify that the first alphabetical level will be used as a reference for all others (see Strain below), while a “sum” constrast means that the reference value is the mean across all levels of that variable (the grand mean).

```
####Specify what should be "appropriate" contrasts
contrasts(di_long$Strain) <- "contr.treatment"###First alphabetical strain will be the baseline!
contrasts(di_long$Batch) <- "contr.sum" ###Batches will be averaged to generate the baseline!
```

## Add a censoring variable

Now that the data has been read into R, and formatted properly, a new variable is added to the data. This variable is called “Useful” and is a binary yes/no variable. The purpose of this variable is marking observations, that are re-observations of a subject that has previously reached disease index 4. As disease index of 4 means 100% wilting, the plant died when it reached this degree of wilting. Since death is permanent, continuing to observe this plant is unlikely to provide new information. To more accurately capture the linear phase of disease development, all observations where no symptoms are visible, except the one on the day before disease onset, will also be assigned to Useful = No

```
###Interesting R-related observation, the below does not work when subsetting is done with filter(), 
###because filter does not retain rownames!
for (i in 1:max(di_long$subject)) { ###Go by subject
  dummy1 <- di_long[di_long$subject==i,] ##Create a first dummy object, that is a subset of the full data containing the current subject 
  if(min(dummy1$DI) == 0){
  #remove those observations that are before disease onset, except the one directly before disease onset.
  dummy1 <- dummy1[dummy1$DPI %in% (max(dummy1$DPI[dummy1$DI==0]):max(dummy1$DPI)),]
  } 
  if (max(dummy1$DI) == 4) { ###If this subject dies at some point
    dummy2 <- dummy1[dummy1$DI==4,] ###Make a new dummy object, that only contains those recordings where DI=4
    NEW <- di_long[di_long$subject==i & (di_long$DPI %in% min(dummy1$DPI):min(dummy2$DPI)),] ###Generate data subset "NEW", which contains those observations for a subject, that are between (including) the last recording where DI=0 and the first recording where disease index is 4.
  } else { ###If this dubject does not die
    NEW <- dummy1 ###New is the same as dummy1
  }
  di_long$Useful[rownames(di_long) %in% rownames(NEW)] <- c("Yes") ###All of those row(names) that are part of the "NEW" object are useful. Therefore these receive status "Yes" in column "Useful"
}
di_long$Useful[which(is.na(di_long$Useful))] <- c("No") ###Those that are not yes (and therefore are NA) become No
rm(dummy1,dummy2)
```

After this and the earlier code blocks have been run, the di\_long data frame should look similar to this:

```
library("broom")
```

```
str(di_long)
```

```
## 'data.frame':    2856 obs. of  7 variables:
##  $ Strain : Factor w/ 7 levels "Strain1","Strain2",..: 1 1 1 1 1 1 1 1 1 1 ...
##   ..- attr(*, "contrasts")= chr "contr.treatment"
##  $ Plant  : Factor w/ 1 level "Medicago": 1 1 1 1 1 1 1 1 1 1 ...
##  $ Batch  : Factor w/ 5 levels "A","B-I","B-II",..: 1 1 1 1 1 1 1 1 1 1 ...
##   ..- attr(*, "contrasts")= chr "contr.sum"
##  $ subject: int  1 2 3 4 5 6 7 8 9 10 ...
##  $ DPI    : atomic  3 3 3 3 3 3 3 3 3 3 ...
##   ..- attr(*, "na.action")=Class 'omit'  int [1:2856] 1 3 5 7 9 11 13 15 17 19 ...
##  $ DI     : num  1 2 0 1 0 0 0 1 2 1 ...
##  $ Useful : chr  "Yes" "Yes" "No" "Yes" ...
##  - attr(*, "na.action")=Class 'omit'  Named int [1:1428] 1541 1542 1543 1544 1545 1546 1547 1548 1549 1550 ...
##   .. ..- attr(*, "names")= chr [1:1428] "1541" "1542" "1543" "1544" ...
```

```
tidy(di_long) ###This can be used to assess the descriptive statistics of the data. It may be adivsable to look at SKEW and KURTOSIS of the DI (response) to determined wether this is approximately normally distributed (both close to 0, if they are above "absolute 1" the data is probably really not normal).
```

```
## Warning: NAs introduced by coercion
```

```
## Warning in FUN(newX[, i], ...): no non-missing arguments to min; returning
## Inf
```

```
## Warning in FUN(newX[, i], ...): no non-missing arguments to max; returning
## -Inf
```

```
##    column    n       mean         sd median    trimmed      mad min  max
## 1 Strain* 2856   4.000000   2.000350    4.0   4.000000   2.9652   1    7
## 2  Plant* 2856   1.000000   0.000000    1.0   1.000000   0.0000   1    1
## 3  Batch* 2856   2.911765   1.442629    3.0   2.889764   1.4826   1    5
## 4 subject 2856 238.500000 137.433123  238.5 238.500000 176.4294   1  476
## 5     DPI 2856   6.470588   2.852076    5.5   6.338583   2.2239   3   11
## 6      DI 2856   1.907563   1.582810    2.0   1.884514   2.9652   0    4
## 7 Useful* 2856        NaN         NA     NA        NaN       NA Inf -Inf
##   range       skew  kurtosis         se
## 1     6 0.00000000 -1.251225 0.03743061
## 2     0        NaN       NaN 0.00000000
## 3     4 0.06609884 -1.342605 0.02699452
## 4   475 0.00000000 -1.201271 2.57165269
## 5     8 0.39572616 -1.327143 0.05336813
## 6     4 0.14288963 -1.518671 0.02961759
## 7  -Inf         NA        NA         NA
```

With this, the data has been imported into R, and formatted according to the needs of *most* of the subsequent analysis. When actively trying out different analysis, it may be advisable to generate a backup copy of the table.

```
di_long_full <- di_long
```

Alternatively, this table can also be exported

```
write.csv(di_long, file = "My_Formatted_Data.csv")
```

# Analysis of the area under the disease progression curve (AUDPC)

Area under the disease progression curve (AUDPC) is a classical approach to analyze disease progression recordings.

## Rationale and data preparation

Here, the area under the curve is calculated, and the curve is drawn using disease index (y) and time (x). To get an overall impression per experiment, one can calculate the average disease index per day, within one experimental group, using:

```
library("dplyr")
```

```
###Below i make use of some of the nice functionality provided by the tidyverse, most remarkably the pipe ( %>% ).
di_summary <- di_long %>% group_by(Plant, Strain, Batch, DPI) %>% 
  summarise(mean(DI),sd(DI),sd(DI)/sqrt(length(DI))) ##calculate within Batch mean, sd, and se for each Plant/Strain combination.
##Averages within each replicate. This summary table is mainly helpful for plotting, not used for analysis...
colnames(di_summary) <- c("Plant", "Strain", "Batch", "DPI", "mean", "sd", "se") ###Assign correct columnnames
```

Using these summaries, one can take a look at the averaged disease progression, using

```
library("tidyverse")
library("stringr")
```

```
ggplot(filter(di_summary, Batch=="A")) + ###Use Only Batch A (too busy otherwise)
  aes(x=DPI,y=mean,color=Strain) + ###Color by Strain, specify x and y.
  geom_area(aes(fill=Strain),position="identity",alpha=0.15) + ###Area plot, colored by Strain
  ##geom_errorbar(aes(x=DPI, ymax=mean+se, ymin=mean-se), size=0.25)+ ###This line adds SE. 
  ##geom_errorbar(aes(x=DPI, ymax=mean+sd, ymin=mean-sd), size=0.25)+ ###This line adds SD. Don't use both.
  facet_wrap(~Strain) + ###One plot per strain 
  labs(x = "Days post infection", y = "Avg. Disease Index") + #Labels
  ggtitle("Disease Areas,\nper strain, for batch A") #Title
```

From this plot, one can see that in the example dataset the areas differ quite drastically between different strains. As can be seen here, all observations can be included in an AUDCP analysis, but one should take care that total observation times are similar, identical if possible. As the area increases with both, increased disease index and prolonged time, experiments of different length should not be compared using this approach. To calculated the actual AUDPC for each individual in the dataset a new data frame is created. As AUDCP is calculated from both disease index and DPI, this can not be stored in a reasonable way in the long data frame generated earlier.

```
library("MESS")
```

```
####Build a table of AUDCPs, per subject
auc_df <- data.frame() ###Make auc_df data frame
for (i in 1:max(di_long$subject)) { ##Go by subject
  temp <- di_long[di_long$subject==i,] ###Subset full table into the subject table
  temp <- droplevels(temp) ###Drop levels, so levels works properly below
  auc_df[i,1] <- i ###Subject number
  auc_df[i,2] <- levels(temp$Strain) ###Strain
  auc_df[i,3] <- levels(temp$Plant) ###Plant
  auc_df[i,4] <- levels(temp$Batch) ###Batch
  auc_df[i,5] <- auc(temp$DPI,temp$DI) ###AUC; i assume that trapezoid rule is fine here. 
  ### Additionally, auc calculation starts with the lowest x (DPI). I think this is sensible
  ### I assume that if one specifies "from=0", the curve is expanded by a triangle that covers the range from 
  ### 0 to whatever is the value at the first observation. I think the first observation should ideally be 0
  ### if data was recorded from the beginning..
}
colnames(auc_df) <- c("subject","Strain","Plant","Batch","AUC") ###Name columns in AUC datafarame
auc_df$Strain <- as.factor(auc_df$Strain) #refactor
auc_df$Plant <- as.factor(auc_df$Plant) #refactor
auc_df$Batch <- as.factor(auc_df$Batch) #refactor
str(auc_df)
```

```
## 'data.frame':    476 obs. of  5 variables:
##  $ subject: int  1 2 3 4 5 6 7 8 9 10 ...
##  $ Strain : Factor w/ 7 levels "Strain1","Strain2",..: 1 1 1 1 1 1 1 1 1 1 ...
##  $ Plant  : Factor w/ 1 level "Medicago": 1 1 1 1 1 1 1 1 1 1 ...
##  $ Batch  : Factor w/ 5 levels "A","B-I","B-II",..: 1 1 1 1 1 1 1 1 1 1 ...
##  $ AUC    : num  24.5 25 17 24.5 21.5 11 22 24.5 24.5 19.5 ...
```

The auc data.frame contains one area under the curve per subject and all other subject specific variables as stored in the original table.

## Analysis of differences in area under the disease progression curve

An initial assessment of strain specific differences in AUDPC can be performed visually, for example by generating boxpots.

```
ggplot(auc_df) + geom_boxplot(
  aes(x=Strain, y=AUC, color=Strain),  #Plot boxplots of AUCs, by strains
  notch=F) +
  labs(y="AUDPC") +
  facet_wrap(~Batch) + ###Individual plots per batch (and plant if applicable)
  ggtitle("Figure 2\nArea Under the Disease Progression Curve per strain across batches")
```

Next, one can use the area under the disease progression curve, to build a linear model, or a linear mixed effects model.

```
library("lme4")
library("lmerTest")
```

```
summary(lm(AUC~Batch,data=auc_df)) ###Can be used to identify batch effects. If there are none, including batch as a random factor below is not necessary (but also not necessarily wrong).
```

```
## 
## Call:
## lm(formula = AUC ~ Batch, data = auc_df)
## 
## Residuals:
##     Min      1Q  Median      3Q     Max 
## -20.115  -6.217   2.261   6.137  12.033 
## 
## Coefficients:
##             Estimate Std. Error t value Pr(>|t|)    
## (Intercept)  15.9888     0.7289  21.935  < 2e-16 ***
## BatchB-I      0.2282     1.0887   0.210  0.83407    
## BatchB-II     4.1265     1.0887   3.790  0.00017 ***
## BatchC-I      0.8738     1.0887   0.803  0.42260    
## BatchC-II    -0.1207     1.0887  -0.111  0.91176    
## ---
## Signif. codes:  0 '***' 0.001 '**' 0.01 '*' 0.05 '.' 0.1 ' ' 1
## 
## Residual standard error: 7.714 on 471 degrees of freedom
## Multiple R-squared:  0.04011,    Adjusted R-squared:  0.03196 
## F-statistic: 4.921 on 4 and 471 DF,  p-value: 0.000679
```

```
auc_lmer <- lmer(
  AUC ~ Strain + (1|Batch),   ### AUC modeled as a function of strain, random effects of batch 
    data = auc_df  ) #Linear mixed effects model.

auc_lm <- lm(AUC~Strain+Batch,data=auc_df) #Linear model.

AIC(auc_lm,auc_lmer) #Lower AIC, better fit, linear model is slightly better.
```

```
##          df      AIC
## auc_lm   12 2828.300
## auc_lmer  9 2832.056
```

```
ggplot(data=auc_df, aes(y=AUC, x=Strain)) +geom_boxplot(aes(colour=Batch)) + ggtitle("Boxplot of AUDCP per strain by experimental batch")
```

The model can be explored using various functions, such as summary.

```
summary(auc_lmer)  ### A model summary, containing information on the model.
```

```
## Linear mixed model fit by REML t-tests use Satterthwaite approximations
##   to degrees of freedom [lmerMod]
## Formula: AUC ~ Strain + (1 | Batch)
##    Data: auc_df
## 
## REML criterion at convergence: 2814.1
## 
## Scaled residuals: 
##     Min      1Q  Median      3Q     Max 
## -4.6437 -0.3615  0.0894  0.4529  3.0154 
## 
## Random effects:
##  Groups   Name        Variance Std.Dev.
##  Batch    (Intercept)  2.92    1.709   
##  Residual             21.69    4.657   
## Number of obs: 476, groups:  Batch, 5
## 
## Fixed effects:
##               Estimate Std. Error       df t value Pr(>|t|)    
## (Intercept)    23.5642     0.9504   8.2000  24.793 5.43e-09 ***
## StrainStrain2 -15.9816     0.7987 465.0000 -20.009  < 2e-16 ***
## StrainStrain3 -15.2500     0.7987 465.0000 -19.093  < 2e-16 ***
## StrainStrain4  -7.6324     0.7987 465.0000  -9.556  < 2e-16 ***
## StrainStrain5  -1.2059     0.7987 465.0000  -1.510   0.1318    
## StrainStrain6  -3.1581     0.7987 465.0000  -3.954 8.89e-05 ***
## StrainStrain7  -2.6691     0.7987 465.0000  -3.342   0.0009 ***
## ---
## Signif. codes:  0 '***' 0.001 '**' 0.01 '*' 0.05 '.' 0.1 ' ' 1
## 
## Correlation of Fixed Effects:
##             (Intr) StrnS2 StrnS3 StrnS4 StrnS5 StrnS6
## StrainStrn2 -0.420                                   
## StrainStrn3 -0.420  0.500                            
## StrainStrn4 -0.420  0.500  0.500                     
## StrainStrn5 -0.420  0.500  0.500  0.500              
## StrainStrn6 -0.420  0.500  0.500  0.500  0.500       
## StrainStrn7 -0.420  0.500  0.500  0.500  0.500  0.500
```

```
tidy(auc_lmer) ### A cleaner display using tidy.
```

```
##                      term   estimate std.error  statistic    group
## 1             (Intercept)  23.564204 0.9504373  24.793011    fixed
## 2           StrainStrain2 -15.981618 0.7987329 -20.008712    fixed
## 3           StrainStrain3 -15.250000 0.7987329 -19.092739    fixed
## 4           StrainStrain4  -7.632353 0.7987329  -9.555575    fixed
## 5           StrainStrain5  -1.205882 0.7987329  -1.509744    fixed
## 6           StrainStrain6  -3.158088 0.7987329  -3.953873    fixed
## 7           StrainStrain7  -2.669118 0.7987329  -3.341690    fixed
## 8    sd_(Intercept).Batch   1.708872        NA         NA    Batch
## 9 sd_Observation.Residual   4.657373        NA         NA Residual
```

```
###The tidy output explained:

#Term: A discription: (Intercept) overall intercept. Intercept depends on the contrasts set initially. Here treatment contrasts are used, so Intercept = First alphabetical strain (Strain1).

#StrainStrain2: Difference in the estimate (slope), between Strain2 and the (Intercept)

#Estimate: The estimated slope
```

Model summaries contain information on the estimated slopes (Estimate) and standard errors, together with a t- and corresponding p-value in the output of the summary function. Note, that the above only contains information on differences between different levels and the reference, which is called (Intercept). The reference is contrast dependent.

It may be quite relevant to know how individual strains compare to each other (and not just how each strain compares to Strain1). This can be analyzed using a generalized linear hypothesis test, while ajusting for multiple comparisons using Tukey’s method.

```
library("multcomp")
library("rcompanion")
library("stringr")
```

```
tidy(summary(glht(auc_lmer, linfct=mcp(Strain="Tukey"))))
```

```
##                  lhs rhs    estimate std.error   statistic      p.value
## 1  Strain2 - Strain1   0 -15.9816176 0.7987329 -20.0087122 0.000000e+00
## 2  Strain3 - Strain1   0 -15.2500000 0.7987329 -19.0927394 0.000000e+00
## 3  Strain4 - Strain1   0  -7.6323529 0.7987329  -9.5555755 0.000000e+00
## 4  Strain5 - Strain1   0  -1.2058824 0.7987329  -1.5097441 7.391830e-01
## 5  Strain6 - Strain1   0  -3.1580882 0.7987329  -3.9538725 1.558965e-03
## 6  Strain7 - Strain1   0  -2.6691176 0.7987329  -3.3416897 1.466401e-02
## 7  Strain3 - Strain2   0   0.7316176 0.7987329   0.9159728 9.701571e-01
## 8  Strain4 - Strain2   0   8.3492647 0.7987329  10.4531367 0.000000e+00
## 9  Strain5 - Strain2   0  14.7757353 0.7987329  18.4989681 0.000000e+00
## 10 Strain6 - Strain2   0  12.8235294 0.7987329  16.0548397 0.000000e+00
## 11 Strain7 - Strain2   0  13.3125000 0.7987329  16.6670225 0.000000e+00
## 12 Strain4 - Strain3   0   7.6176471 0.7987329   9.5371640 0.000000e+00
## 13 Strain5 - Strain3   0  14.0441176 0.7987329  17.5829953 0.000000e+00
## 14 Strain6 - Strain3   0  12.0919118 0.7987329  15.1388669 0.000000e+00
## 15 Strain7 - Strain3   0  12.5808824 0.7987329  15.7510497 0.000000e+00
## 16 Strain5 - Strain4   0   6.4264706 0.7987329   8.0458314 4.440892e-15
## 17 Strain6 - Strain4   0   4.4742647 0.7987329   5.6017030 2.263718e-07
## 18 Strain7 - Strain4   0   4.9632353 0.7987329   6.2138858 3.836682e-09
## 19 Strain6 - Strain5   0  -1.9522059 0.7987329  -2.4441284 1.800268e-01
## 20 Strain7 - Strain5   0  -1.4632353 0.7987329  -1.8319456 5.262924e-01
## 21 Strain7 - Strain6   0   0.4889706 0.7987329   0.6121828 9.964592e-01
```

This information can, for example, be integrated into a boxplot of the individual disease areas. For example, using a compact letter display, in conbination with AUDPC, combines statistical and visual information. First, however, the grouping letters need to be calculated. Then, these letters are added to the boxplot generated earlier.

```
auc_cld <- cld(glht(auc_lmer, linfct=mcp(Strain="Tukey"))) ###Save letters
```

```
## Warning in RET$pfunction("adjusted", ...): Completion with error > abseps
```

```
auc_cld <- cbind(levels(auc_df$Strain),auc_cld$mcletters$Letters) ###bind letters to columns
colnames(auc_cld) <- c("Strain","Letter") ###Name columns
auc_cld <- as.data.frame(auc_cld) #Coerce to dataframe
###Integrate letters into auc_df##
auc_df <- left_join(auc_df,auc_cld,by="Strain",copy=T) ###Add letter information
###Some extra scripting to make the mean and CI plot.
auc_CI <- as.data.frame(tidy(confint(auc_lmer)))
```

```
## Computing profile confidence intervals ...
```

```
auc_CI <- auc_CI[3:nrow(auc_CI),] ##Drop sig01, sigma
auc_CI$Strain <- levels(auc_df$Strain)
###Mean relative to Strain1 (except strain1 that one is absolute)

for (i in 1:nrow(auc_CI)){
  if (i==1){
    auc_CI$mean[i] <- mean(c(auc_CI$X2.5..[i],auc_CI$X97.5..[i]))
    auc_CI$upr[i] <- auc_CI$X97.5..[i]
    auc_CI$lwr[i] <- auc_CI$X2.5..[i]
  } else {
    auc_CI$mean[i] <- c(auc_CI$mean[1]+mean(c(auc_CI$X2.5..[i],auc_CI$X97.5..[i])))
    auc_CI$upr[i] <- c(auc_CI$mean[1]+auc_CI$X97.5..[i])
    auc_CI$lwr[i] <- c(auc_CI$mean[1]+auc_CI$X2.5..[i])
  }
}
 
####Generate plot of meanCI of AUDCP with significance letters and raw data as jittered points
  ggplot(aes(x=Strain, y=AUC, color=Strain),data=auc_df) + ###Plot the auc_df
  geom_crossbar(data = auc_CI, aes(x = Strain, y = mean, ymin = lwr, ymax = upr,fill=Strain), alpha=0.3) +
  geom_jitter(aes(shape=Batch)) + ###with jitter overplotted, symbol shape defined by batch
  geom_text(aes(x=Strain, y=-3, label=Letter),color="black", data=auc_cld) + ###Get the letters from auc_cld 
  #and write those to position y=-3
  labs(y="AUDPC") + #Y-Axis label
  ggtitle("AUDPC raw values and mean from the LMM\nwith 95% CI per strain and grouping letters") #Title
```

Finally, one can plot the pairwise difference in AUDPC means with confidence intervals for the linear model and the linear mixed effects model.

```
pairwise_confint_AUDPC_lm <- as.data.frame(confint(glht(auc_lm, mcp(Strain = "Tukey")))$confint)
pairwise_confint_AUDPC_lm$Comparison <- rownames(pairwise_confint_AUDPC_lm)

pairwise_confint_AUDPC_lmer <- as.data.frame(confint(glht(auc_lmer, mcp(Strain = "Tukey")))$confint)
pairwise_confint_AUDPC_lmer$Comparison <- rownames(pairwise_confint_AUDPC_lmer)

###Plot the comparisons, below may not be the most straight-foward way to plot this the way I want it, but it works.
ggplot(pairwise_confint_AUDPC_lm, aes(x = Comparison, y = Estimate, ymin = lwr, ymax = upr, color = Comparison)) + ###Plot Comparison on x, estimate on y
  scale_x_discrete(limits = rev(levels(as.factor(pairwise_confint_AUDPC_lm$Comparison)))) + ###Rescale x, so the order is inverted 
  geom_errorbar() + geom_point() + ###Draw data
  coord_flip() +  theme(legend.position="none") + xlab("") +###Invert X and Y, hide legend
  ggtitle("Difference in means of the AUDPC \nin the linear model with 95% confidence interval") ##Add a title
```

```
####Plot of the comparisons in the LMM. Here the bars are colored by their absolute value, instead of by their name.
ggplot(pairwise_confint_AUDPC_lmer, aes(x = Comparison, y = Estimate, ymin = lwr, ymax = upr, color = abs(Estimate))) + ###Plot Comparison on x, estimate on y
  scale_x_discrete(limits = rev(levels(as.factor(pairwise_confint_AUDPC_lmer$Comparison)))) + ###Rescale x, so the order is inverted 
  geom_errorbar() + geom_point() + ###Draw data
  coord_flip() +  theme(legend.position="none") + xlab("") +###Invert X and Y, hide legend
  ggtitle("Difference in means of the AUDPC \nin the LMM with 95% confidence interval") ##Add a title
```

Estimates of lm and lmm are very similar. As a rule of thumb, comparisons where “0” is not part of the 95% confidence interval are likely to produce a signficant p-value (assuming significance is denoted by p<0.05).

# Analysis of Disease development

## Using a Repeated Measure ANOVA

Repeated-measure ANOVA can be used to analyze DI and time. However, when using repeated measure ANOVA one should be aware that the arrow of time is not considered in this analysis. Here the variable denoting the measurements is put into the Error() term. *This section is one of the few parts that require user input to define the split list*.

```
#Linear model of rep measure is as follows
contrasts(di_long$Strain) <- "contr.sum"
rm_aov <- aov(DI~Strain + Error(DPI), data = di_long)
###The below Provides differences to Strain1. 
summary(rm_aov, split=list(Strain = list(Strain2=1,Strain3=2,Strain4=3,Strain5=4,Strain6=5,Strain7=6)))
```

```
## 
## Error: DPI
##           Df Sum Sq Mean Sq F value Pr(>F)
## Residuals  1   2479    2479               
## 
## Error: Within
##                     Df Sum Sq Mean Sq F value   Pr(>F)    
## Strain               6   1104   184.0  146.79  < 2e-16 ***
##   Strain: Strain2    1     18    18.4   14.67 0.000131 ***
##   Strain: Strain3    1    667   666.7  531.94  < 2e-16 ***
##   Strain: Strain4    1    215   214.8  171.39  < 2e-16 ***
##   Strain: Strain5    1     40    40.4   32.27 1.47e-08 ***
##   Strain: Strain6    1    129   129.1  103.00  < 2e-16 ***
##   Strain: Strain7    1     34    34.4   27.46 1.72e-07 ***
## Residuals         2848   3569     1.3                     
## ---
## Signif. codes:  0 '***' 0.001 '**' 0.01 '*' 0.05 '.' 0.1 ' ' 1
```

```
###I personally think that LMMs are nicer to investigate and offer greater flexibility.
```

## Linear mixed effects model

### Rationale

In this section, the data is analyzed using a linear mixed effect model. While such models have been used already in the previous section, to test for a strain specific influence on the area under the disease progression curve, different data is used to build the model in this section. As mentioned, AUDPC summarizes disease incidence and time into a single variable, the area. However, in certain cases the AUDCP could be very similar, while the actual disease progression is different. Taking a look at Figure 1, we see that Strain2 and Strain3 display different disease progressions. For Strain 2, the Disease Index continually rises over time. For Strain3, the the disease appears to set on very quickly, in fact before the observations started, but from there it does not increase in severity. However, if we compare the impact of those two strains on the AUDCP (Fig2, 3), it becomes evident, that the AUDPCs for these strains are not statistically different, both strains belong to group “a”. Other methods may be more sensitive to such differences. Below a different approach using linear mixed effect models is taken.

### Model

The main difference between this analysis and the one based on the AUDPC is the response variable. In the AUDPC analysis the area was the response variable. Here in this section, the response variable is the Disease Index, and time is included as predictor (covariate) in the model. A linear mixed effects model is generated. Here, the DI is modeled on the fixed effects “Strain” and an interaction of Strain and Time. Batch is included as a random effect, meaning it is not of direct interest, but assumed to introduce variation, specifically by affecting the intercept.

```
###Define contrasts for lmer
contrasts(di_long$Strain) <- "contr.treatment"###First alphabetical strain will be the baseline!
#contrasts(di_long$Plant) <- "contr.treatment"###First alphabetical plant will be the baseline!
contrasts(di_long$Batch) <- "contr.sum" ###Batches will be averaged
###Drop things that are not "Useful"
di_long_useful <- filter(di_long, Useful=="Yes")
str(di_long_useful)
```

```
## 'data.frame':    1901 obs. of  7 variables:
##  $ Strain : Factor w/ 7 levels "Strain1","Strain2",..: 1 1 1 1 1 1 1 1 1 1 ...
##   ..- attr(*, "contrasts")= chr "contr.treatment"
##  $ Plant  : Factor w/ 1 level "Medicago": 1 1 1 1 1 1 1 1 1 1 ...
##  $ Batch  : Factor w/ 5 levels "A","B-I","B-II",..: 1 1 1 1 1 1 1 1 1 1 ...
##   ..- attr(*, "contrasts")= chr "contr.sum"
##  $ subject: int  1 2 4 5 7 8 9 10 11 12 ...
##  $ DPI    : atomic  3 3 3 3 3 3 3 3 3 3 ...
##   ..- attr(*, "na.action")=Class 'omit'  int [1:2856] 1 3 5 7 9 11 13 15 17 19 ...
##  $ DI     : num  1 2 1 0 0 1 2 1 0 2 ...
##  $ Useful : chr  "Yes" "Yes" "Yes" "Yes" ...
##  - attr(*, "na.action")=Class 'omit'  Named int [1:1428] 1541 1542 1543 1544 1545 1546 1547 1548 1549 1550 ...
##   .. ..- attr(*, "names")= chr [1:1428] "1541" "1542" "1543" "1544" ...
```

```
## Build linear mixed effect model(s) ####
## only Strain as fixed effect (a simple model),
## Batch and subject are assumed to have random effects.
## Strain and time are assumed to interact, meaning that for each day, the strain specific slope is estimated.
## See page 6 (Table2) of the lme4 vignette on how to construct error terms
## This model is supposed to capture disease development, so it will work with the data previously deemed "useful"

disease_lmer <- lmer(DI~Strain+Strain:DPI +(1|Batch)+(1|subject),data=di_long_useful)
```

The model can be investigated using summary functions. The pairwise comparisons can be plotted with 95%CI to assess how different two strains are.

```
###Check model summary
summary(disease_lmer)
```

```
## Linear mixed model fit by REML t-tests use Satterthwaite approximations
##   to degrees of freedom [lmerMod]
## Formula: DI ~ Strain + Strain:DPI + (1 | Batch) + (1 | subject)
##    Data: di_long_useful
## 
## REML criterion at convergence: 4885.9
## 
## Scaled residuals: 
##     Min      1Q  Median      3Q     Max 
## -3.4325 -0.3999  0.0021  0.3596  3.2849 
## 
## Random effects:
##  Groups   Name        Variance Std.Dev.
##  subject  (Intercept) 1.0381   1.0189  
##  Batch    (Intercept) 0.1678   0.4097  
##  Residual             0.4169   0.6457  
## Number of obs: 1901, groups:  subject, 476; Batch, 5
## 
## Fixed effects:
##                     Estimate Std. Error         df t value Pr(>|t|)    
## (Intercept)         -2.15059    0.26604   15.30000  -8.084 6.48e-07 ***
## StrainStrain2       -1.36731    0.32617 1553.00000  -4.192 2.92e-05 ***
## StrainStrain3        3.03661    0.24256  898.40000  12.519  < 2e-16 ***
## StrainStrain4        4.06498    0.24253  897.90000  16.761  < 2e-16 ***
## StrainStrain5       -1.45363    0.28979 1445.50000  -5.016 5.92e-07 ***
## StrainStrain6       -1.20492    0.28924 1405.90000  -4.166 3.29e-05 ***
## StrainStrain7       -1.06011    0.29048 1342.10000  -3.649 0.000273 ***
## StrainStrain1:DPI    0.84087    0.02675 1257.30000  31.434  < 2e-16 ***
## StrainStrain2:DPI    0.54726    0.02588 1611.70000  21.144  < 2e-16 ***
## StrainStrain3:DPI    0.02389    0.01133 1201.70000   2.108 0.035206 *  
## StrainStrain4:DPI    0.01727    0.01129 1201.40000   1.529 0.126461    
## StrainStrain5:DPI    1.06117    0.03181 1303.10000  33.363  < 2e-16 ***
## StrainStrain6:DPI    0.93431    0.02893 1416.40000  32.295  < 2e-16 ***
## StrainStrain7:DPI    0.92674    0.03029 1575.40000  30.597  < 2e-16 ***
## ---
## Signif. codes:  0 '***' 0.001 '**' 0.01 '*' 0.05 '.' 0.1 ' ' 1
```

```
## 
## Correlation matrix not shown by default, as p = 14 > 12.
## Use print(x, correlation=TRUE)  or
##   vcov(x)     if you need it
```

```
###E.g. plot confints
###Make pairwise confints and plot them, with a flipped coordinate system
pairwise_confint <- as.data.frame(confint(glht(disease_lmer, mcp(Strain = "Tukey", interaction_average=T)))$confint)
pairwise_confint$Comparison <- rownames(pairwise_confint)

ggplot(pairwise_confint, aes(x = Comparison, y = Estimate, ymin = lwr, ymax = upr, color = abs(Estimate))) + ###Plot Comparison on x, estimate on y
  scale_x_discrete(limits = rev(levels(as.factor(pairwise_confint$Comparison)))) + ###Rescale x, so the order is inverted 
  geom_errorbar() + geom_point() + ###Draw data
  coord_flip() +  theme(legend.position="none") + xlab("") + ###Invert X and Y, hide legend
  ggtitle("Difference in means with 95% confidence interval \ncolored by absolute estimated difference") ##Add a title
```

```
confint_model <- as.data.frame(tidy(confint(disease_lmer)))
```

```
## Computing profile confidence intervals ...
```

```
confint_slopes <- confint_model[ (1 + 3 + nlevels( di_long$Strain ) ) : ( 3 + 2*nlevels( di_long$Strain ) ) , 2:3 ]

colnames(confint_slopes) <- c("lwr","upr")
confint_slopes$Estimate <- rowMeans(confint_slopes)
confint_slopes$Strain <- levels(di_long$Strain)

###Plot the estimates, below may not be the most straight-foward way to plot this the way I want it, but it works.
ggplot(confint_slopes, aes(x = Strain, y = Estimate, ymin = lwr, ymax = upr, color = abs(Estimate))) + ###Plot Comparison on x, estimate on y
  scale_x_discrete(limits = rev(levels(as.factor(confint_slopes$Strain)))) + ###Rescale x, so the order is inverted 
  geom_errorbar() + geom_point() + ###Draw data
  coord_flip() +  theme(legend.position="none") + xlab("") +###Invert X and Y, hide legend
  ggtitle("Absolute slopes with 95%CI") ##Add a title
```

```
#Intercepts are treatment contrasted
confint_icep <- confint_model[4:( 3 + nlevels( di_long$Strain )),]

confint_icep$Strain <- levels(di_long$Strain)

for (i in 1:nrow(confint_icep)){
  if (i==1){
    confint_icep$Estimate[i] <- mean(c(confint_icep$X2.5..[i],confint_icep$X97.5..[i]))
    confint_icep$upr[i] <- confint_icep$X97.5..[i]
    confint_icep$lwr[i] <- confint_icep$X2.5..[i]
  } else {
    confint_icep$Estimate[i] <- c(confint_icep$Estimate[1]+mean(c(confint_icep$X2.5..[i],confint_icep$X97.5..[i])))
    confint_icep$upr[i] <- c(confint_icep$Estimate[1]+confint_icep$X97.5..[i])
    confint_icep$lwr[i] <- c(confint_icep$Estimate[1]+confint_icep$X2.5..[i])
  }
}

ggplot(confint_icep, aes(x = Strain, y = Estimate, ymin = lwr, ymax = upr, color = abs(Estimate))) + ###Plot Comparison on x, estimate on y
  scale_x_discrete(limits = rev(levels(as.factor(confint_icep$Strain)))) + ###Rescale x, so the order is inverted 
  geom_errorbar() + geom_point() + ###Draw data
  coord_flip() +  theme(legend.position="none") + xlab("") +###Invert X and Y, hide legend
  ggtitle("Absolute intercept with 95%CI") ##Add a title
```

Essentially, all of the tools used to analyze the linear model for area under the disease progression curve, can be applied to the disease model:

```
###Test hypothesis that all strains are equal and do compact letter grouping
#Using multcomp glht
summary(glht(disease_lmer, linfct=mcp(Strain="Tukey",interaction_average = T)))
```

```
## 
##   Simultaneous Tests for General Linear Hypotheses
## 
## Multiple Comparisons of Means: Tukey Contrasts
## 
## 
## Fit: lme4::lmer(formula = DI ~ Strain + Strain:DPI + (1 | Batch) + 
##     (1 | subject), data = di_long_useful)
## 
## Linear Hypotheses:
##                        Estimate Std. Error z value Pr(>|z|)    
## Strain2 - Strain1 == 0 -1.36731    0.32617  -4.192  < 0.001 ***
## Strain3 - Strain1 == 0  3.03661    0.24256  12.519  < 0.001 ***
## Strain4 - Strain1 == 0  4.06498    0.24253  16.761  < 0.001 ***
## Strain5 - Strain1 == 0 -1.45363    0.28979  -5.016  < 0.001 ***
## Strain6 - Strain1 == 0 -1.20492    0.28924  -4.166  < 0.001 ***
## Strain7 - Strain1 == 0 -1.06011    0.29048  -3.649  0.00489 ** 
## Strain3 - Strain2 == 0  4.40392    0.30144  14.610  < 0.001 ***
## Strain4 - Strain2 == 0  5.43230    0.30141  18.023  < 0.001 ***
## Strain5 - Strain2 == 0 -0.08632    0.34046  -0.254  0.99998    
## Strain6 - Strain2 == 0  0.16239    0.34021   0.477  0.99909    
## Strain7 - Strain2 == 0  0.30721    0.34086   0.901  0.97144    
## Strain4 - Strain3 == 0  1.02838    0.20802   4.944  < 0.001 ***
## Strain5 - Strain3 == 0 -4.49024    0.26164 -17.162  < 0.001 ***
## Strain6 - Strain3 == 0 -4.24153    0.26102 -16.250  < 0.001 ***
## Strain7 - Strain3 == 0 -4.09671    0.26242 -15.611  < 0.001 ***
## Strain5 - Strain4 == 0 -5.51861    0.26161 -21.095  < 0.001 ***
## Strain6 - Strain4 == 0 -5.26991    0.26098 -20.193  < 0.001 ***
## Strain7 - Strain4 == 0 -5.12509    0.26239 -19.532  < 0.001 ***
## Strain6 - Strain5 == 0  0.24871    0.30542   0.814  0.98295    
## Strain7 - Strain5 == 0  0.39352    0.30648   1.284  0.85537    
## Strain7 - Strain6 == 0  0.14482    0.30607   0.473  0.99913    
## ---
## Signif. codes:  0 '***' 0.001 '**' 0.01 '*' 0.05 '.' 0.1 ' ' 1
## (Adjusted p values reported -- single-step method)
```

```
cld(glht(disease_lmer, linfct=mcp(Strain="Tukey", interaction_average=T)))
```

```
## Strain1 Strain2 Strain3 Strain4 Strain5 Strain6 Strain7 
##     "d"     "b"     "a"     "c"     "b"     "b"     "b"
```

```
#Using lmerTest lsMeans
lmerlsm <- difflsmeans(disease_lmer)$diffs.lsmeans.table
lmerlsm
```

```
##                          Estimate Standard Error    DF t-value Lower CI
## Strain Strain1 - Strain2   3.1598         0.2013 400.9   15.69   2.7640
## Strain Strain1 - Strain3   1.9512         0.1839 289.5   10.61   1.5893
## Strain Strain1 - Strain4   0.9633         0.1838 289.5    5.24   0.6014
## Strain Strain1 - Strain5   0.1087         0.1879 318.5    0.58  -0.2609
## Strain Strain1 - Strain6   0.6344         0.1867 309.9    3.40   0.2670
## Strain Strain1 - Strain7   0.5358         0.1872 313.2    2.86   0.1676
## Strain Strain2 - Strain3  -1.2086         0.1984 376.2   -6.09  -1.5987
## Strain Strain2 - Strain4  -2.1966         0.1984 376.2  -11.07  -2.5866
## Strain Strain2 - Strain5  -3.0512         0.2021 407.5  -15.10  -3.4484
## Strain Strain2 - Strain6  -2.5254         0.2010 398.3  -12.56  -2.9207
## Strain Strain2 - Strain7  -2.6240         0.2015 401.8  -13.03  -3.0200
## Strain Strain3 - Strain4  -0.9880         0.1806 267.4   -5.47  -1.3435
## Strain Strain3 - Strain5  -1.8425         0.1847 295.4   -9.98  -2.2060
## Strain Strain3 - Strain6  -1.3168         0.1835 287.1   -7.17  -1.6780
## Strain Strain3 - Strain7  -1.4154         0.1840 290.3   -7.69  -1.7775
## Strain Strain4 - Strain5  -0.8546         0.1847 295.3   -4.63  -1.2180
## Strain Strain4 - Strain6  -0.3288         0.1835 287.1   -1.79  -0.6901
## Strain Strain4 - Strain7  -0.4274         0.1840 290.2   -2.32  -0.7895
## Strain Strain5 - Strain6   0.5257         0.1875 316.0    2.80   0.1568
## Strain Strain5 - Strain7   0.4272         0.1880 319.3    2.27   0.0573
## Strain Strain6 - Strain7  -0.0986         0.1869 310.7   -0.53  -0.4662
##                          Upper CI p-value
## Strain Strain1 - Strain2   3.5556  0.0000
## Strain Strain1 - Strain3   2.3131  0.0000
## Strain Strain1 - Strain4   1.3251  0.0000
## Strain Strain1 - Strain5   0.4783  0.5634
## Strain Strain1 - Strain6   1.0018  0.0008
## Strain Strain1 - Strain7   0.9041  0.0045
## Strain Strain2 - Strain3  -0.8185  0.0000
## Strain Strain2 - Strain4  -1.8065  0.0000
## Strain Strain2 - Strain5  -2.6539  0.0000
## Strain Strain2 - Strain6  -2.1302  0.0000
## Strain Strain2 - Strain7  -2.2279  0.0000
## Strain Strain3 - Strain4  -0.6324  0.0000
## Strain Strain3 - Strain5  -1.4791  0.0000
## Strain Strain3 - Strain6  -0.9556  0.0000
## Strain Strain3 - Strain7  -1.0533  0.0000
## Strain Strain4 - Strain5  -0.4912  0.0000
## Strain Strain4 - Strain6   0.0324  0.0742
## Strain Strain4 - Strain7  -0.0653  0.0209
## Strain Strain5 - Strain6   0.8947  0.0054
## Strain Strain5 - Strain7   0.7970  0.0237
## Strain Strain6 - Strain7   0.2691  0.5982
```

```
Comparison = str_split_fixed(rownames(lmerlsm),"Strain ",2)[,2]

### Produce compact letter display

library(rcompanion)

cldList(comparison = Comparison,
        p.value    = p.adjust(lmerlsm$'p-value',
                       method =  "bonferroni")   ,
        threshold = 0.05)
```

```
##     Group Letter MonoLetter
## 1 Strain1      a      a    
## 2 Strain2      b       b   
## 3 Strain3      c        c  
## 4 Strain4      d         d 
## 5 Strain5     ae      a   e
## 6 Strain6     de         de
## 7 Strain7    ade      a  de
```

Using the linear model and raw data, different displays can be plotted, for example, a boxplot of the “Useful” data-points combined with the predictions (extrapolations) of the linear model.

```
## 
## Attaching package: 'modelr'
```

```
## The following object is masked from 'package:broom':
## 
##     bootstrap
```

```
##Add predictions to full dataset
di_long <- add_predictions(di_long,disease_lmer,var="lmer.pred")
```

```
## Warning: contrasts dropped from factor Strain
```

```
ggplot(data=di_long, aes(x=DPI,y=DI))+
  geom_boxplot(aes(color=Strain,group=DPI),data=filter(di_long, Useful=="Yes"))+
  geom_smooth(aes(y=lmer.pred,color=Strain), method="lm", alpha=0.6) +
  facet_wrap(~Strain)+
  ggtitle("Boxplots and linear fit for individual strains")
```

Finally, of the methods discussed here, those relying on a linear model of raw observations over time, can be used for other kinds of observations, e.g. bacterial titers.

At least briefly, plot residuals of the lmm to assess if this appears normal (random)

```
di_long$resid[di_long$Useful=="Yes"] <- resid(disease_lmer)
ggplot(di_long[di_long$Useful=="Yes",], aes(x=DPI,y=resid)) + geom_jitter(aes(color=Strain))
```

# Survival Analysis

## Background

Survival analysis is different in several aspects from the earlier approaches. Survival analysis, also known that time-to-event analysis, builds on a different dataset, which can be generated from raw disease indices. Survival analysis provides a way to analyze the time-to-event recordings within one population. In the case of the data used here, what is of interest is an event that can be referred to as *death*. Correctly defining *death* in this context is crucial for the outcome of the analysis. *Death*, here, is defined as a subject reaching a certain disease index, from which it cannot recover. In this script the disease index that defines the threashold to *death* is called “cutoff”.

```
###Set the cutoff by assigning a number to the variable cutoff
cutoff <- c(2.5)
```

All of the observations that are above the dotted line in the plot below are *dead*, at the day they cross that line. Those that never cross the line are alive until the end of observations, and are “right censored”, meaning that their event was not observed during the time this subject was observed.

```
ggplot(data=di_long) +
                  geom_jitter(aes(x=DPI, y=DI,color=Strain,shape=Batch)) +
                  geom_segment(aes(x=0, xend=max(DPI)+0.5,y=cutoff, yend=cutoff), linetype="dotted") +
                  labs(x = "Days post infection", y = "Disease indices", title="Scatterplot of disease indices\n cutoff plotted as dotted line") +
                  coord_cartesian(xlim=c(-0.1,10))
```

## Generation of a survival table

A “survival table” can be generated using the following code. This code works on the long table, generated in the beginning, and the cutoff variable defined above.

```
###Generate survival table
surv_from_DI <- data.frame(Subject=disease_index$subject,
                         Strain=disease_index$Strain,
                         Plant=disease_index$Plant,
                         Batch=disease_index$Batch)
###Fill survival table based on the di_long table. This generates warnings. These can be ignored and come from the min()
for (i in 1:max(di_long$subject)) { #Go by subject
  dummy <- di_long[di_long$subject==i,] #generate dummy for the subject
  if (is.infinite(min(dummy$DPI[which(dummy$DI >= cutoff)]))) { #If none of the DI is greater than the cutoff (this is where warnings are generated, min on an empty object returns infinite and a warning!)
    surv_from_DI$End[i] <- max(dummy$DPI) #Generate a  observation, censoring at the maximum DPI recorded
    surv_from_DI$Death[i] <- 0 #Still alive, because it did not pass the cutoff
  } else { #If more than zero DI are greater than the cutoff
    surv_from_DI$End[i] <- min(dummy$DPI[which(dummy$DI >= cutoff)]) #Use the lowest DPI where condition is met
    surv_from_DI$Death[i] <- 1 #record as dead
  }
}
rm(dummy)
```

## Kaplan-Meier estimates of survival

Kaplan-Meier estimates of survival are the basic tool of survival analysis. These can be estimated using the survfit function from the “survival” package.c

```
library("survival")
```

```
surv_DI_fit <- survfit(Surv(End, Death) ~Strain +strata(Batch), data=surv_from_DI)
```

The survminer package provides the ggsurvplot() function. This works nicely on datasets with few treatments. However, for the data presented here, I think it is easier to initially generate a data frame that contains the fit and plot with “normal” ggplot2

```
library("stringr")
###Strata dummy generation, modified from kmiddleton / rexamples 
strata_dummy <-NULL
for(i in 1:length(surv_DI_fit$strata)){
      # add vector for one strata according to number of rows of strata
      strata_dummy <- c(strata_dummy, rep(names(surv_DI_fit$strata)[i], surv_DI_fit$strata[i]))
}
###Data frame generation inspired by a post by Hadley Wickham to the ggplot2 Googlegroup
surv_DI_fit.df <- data.frame( 
  time = surv_DI_fit$time, 
  n.risk = surv_DI_fit$n.risk, 
  n.event = surv_DI_fit$n.event, 
  surv = surv_DI_fit$surv, 
  strata = strata_dummy, 
  upper = surv_DI_fit$upper, 
  lower = surv_DI_fit$lower 
) 
zeros <- data.frame(time = 0, surv = 1, strata = names((surv_DI_fit$strata)), 
  upper = 1, lower = 1)

surv_DI_fit.df <- plyr::rbind.fill(zeros, surv_DI_fit.df) ###I dont want to load plyr because i guess it will interfere with dplyr...
rm(strata_dummy)
rm(zeros)

##The Batch and Strain columns are regenerated from the strata field, there are probably more elegant ways to do this

surv_DI_fit.df$Batch <- as.factor( str_split_fixed(
  matrix( nrow=length(surv_DI_fit.df$strata),ncol=2, unlist(strsplit(as.character(surv_DI_fit.df$strata),", ")), byrow=T )[,2],"=",2)[,2])
surv_DI_fit.df$Strain <- as.factor( str_split_fixed(
    matrix( nrow=length(surv_DI_fit.df$strata),ncol=2, unlist(strsplit(as.character(surv_DI_fit.df$strata),", ")), byrow=T )[,1],"=",2)[,2])

###End of data frame generation
###Start plotting
ggplot(surv_DI_fit.df, aes(time, surv, colour = Strain)) + 
  geom_step(aes(y = surv*100,linetype=Batch)) +
  facet_wrap(~Strain) +
  ggtitle("Survival estimates for all Batches")
```

## Cox-Proportional hazards and hazard ratios

Different approaches to survival analysis, are based on analysing the hazards. The hazard is the probability of experiencing an event at a given timepoint. Many hazard based analysis assume that hazards are proportional between treatments, meaning that they differ by a fixed factor. Hazards were strongly influenced by Cox and a basic model is the cox proportional hazards model.

```
###Cox-Proportional hazards####
#Build model
srv_coxph <- coxph(Surv(End, Death) ~Strain + strata(Batch), data=surv_from_DI) 
###Check porportionality of hazards
cox.zph(srv_coxph, transform = "log")
```

```
##                   rho   chisq       p
## StrainStrain2 0.18015  9.3930 0.00218
## StrainStrain3 0.14624  5.9522 0.01470
## StrainStrain4 0.05944  1.0938 0.29564
## StrainStrain5 0.02911  0.2734 0.60104
## StrainStrain6 0.10233  3.3698 0.06640
## StrainStrain7 0.00789  0.0202 0.88708
## GLOBAL             NA 15.4792 0.01684
```

If the proportionality of hazards is violated within the dataset can be seen from the p-value(s) returned by cox.zph. One can then continue with a comparison of hazard ratios, however hazard ratios are not appropriate if the assumption of proportional hazards is violated.

```
library("survcomp")
library("rms")
```

```
####Hazard ratio
haz_rats <- hazard.ratio(x= surv_from_DI$Strain, 
                         surv.time = surv_from_DI$End, 
                         surv.event = surv_from_DI$Death, 
                         strat = surv_from_DI$Batch, 
                         method.test = "wald" ) ###Overall hazard ratios

###Pairwise hazard ratios / modified from the pairwise chisq calculation
pw_hazrats <- matrix(0., nlevels(surv_from_DI$Strain),nlevels(surv_from_DI$Strain))
for (i in 1:nlevels(surv_from_DI$Strain)) {
  for (j in (1:nlevels(surv_from_DI$Strain))[-i]) {
    datasubset <- droplevels(subset( surv_from_DI,
      surv_from_DI$Strain %in% (unique(surv_from_DI$Strain))[c(i,j)]))
    temp <- hazard.ratio(
      x= datasubset$Strain, 
      surv.time = datasubset$End, 
      surv.event = datasubset$Death, 
      strat = datasubset$Batch, 
      method.test = "likelihood.ratio" ###Define test to determine p.
      )
    pw_hazrats[i,j] <- temp$p.value
    }
}
```

```
## Warning in fitter(X, Y, strats, offset, init, control, weights = weights, :
## Loglik converged before variable 1 ; beta may be infinite.

## Warning in fitter(X, Y, strats, offset, init, control, weights = weights, :
## Loglik converged before variable 1 ; beta may be infinite.

## Warning in fitter(X, Y, strats, offset, init, control, weights = weights, :
## Loglik converged before variable 1 ; beta may be infinite.

## Warning in fitter(X, Y, strats, offset, init, control, weights = weights, :
## Loglik converged before variable 1 ; beta may be infinite.
```

```
colnames(pw_hazrats) <- levels(surv_from_DI$Strain)
rownames(pw_hazrats) <- levels(surv_from_DI$Strain)
```

```
stargazer::stargazer(pw_hazrats,type="html",title="Pairwise hazard ratio pvalues")
```

**Pairwise hazard ratio pvalues**

|  | | | | | | | |
|  | Strain1 | Strain2 | Strain3 | Strain4 | Strain5 | Strain6 | Strain7 |
|  | | | | | | | |
| Strain1 | 0 | 0 | 0 | 0 | 0.103 | 0.0002 | 0.008 |
| Strain2 | 0 | 0 | 0 | 0 | 0 | 0 | 0 |
| Strain3 | 0 | 0 | 0 | 0.486 | 0 | 0 | 0 |
| Strain4 | 0 | 0 | 0.486 | 0 | 0 | 0 | 0 |
| Strain5 | 0.103 | 0 | 0 | 0 | 0 | 0.032 | 0.277 |
| Strain6 | 0.0002 | 0 | 0 | 0 | 0.032 | 0 | 0.370 |
| Strain7 | 0.008 | 0 | 0 | 0 | 0.277 | 0.370 | 0 |
|  | | | | | | | |

If the hazards are found to be non-proportional, as is observed here, it might be a good idea to perform survival regression analysis, or pairwise log-rank testing (see below) instead of hazard ratio tests. Alternatives may also come from the use of Cox mixed-effects model, similar to linear mixed-effects model, but with a different type of response variable.

```
library("coxme")
```

```
## Loading required package: bdsmatrix
```

```
## 
## Attaching package: 'bdsmatrix'
```

```
## The following object is masked from 'package:SparseM':
## 
##     backsolve
```

```
## The following object is masked from 'package:base':
## 
##     backsolve
```

```
cme <-  coxme(Surv(End, Death) ~Strain + (1|Batch), data=surv_from_DI)
anova(cme)
```

```
## Analysis of Deviance Table
##  Cox model: response is Surv(End, Death)
## Terms added sequentially (first to last)
## 
##         loglik  Chisq Df Pr(>|Chi|)    
## NULL   -1815.3                         
## Strain -1548.2 534.22  6  < 2.2e-16 ***
## ---
## Signif. codes:  0 '***' 0.001 '**' 0.01 '*' 0.05 '.' 0.1 ' ' 1
```

```
summary(glht(cme,linfct=mcp(Strain="Tukey")))
```

```
## 
##   Simultaneous Tests for General Linear Hypotheses
## 
## Multiple Comparisons of Means: Tukey Contrasts
## 
## 
## Fit: coxme(formula = Surv(End, Death) ~ Strain + (1 | Batch), data = surv_from_DI)
## 
## Linear Hypotheses:
##                        Estimate Std. Error z value Pr(>|z|)    
## Strain2 - Strain1 == 0  -2.6103     0.2236 -11.676   <0.001 ***
## Strain3 - Strain1 == 0  -4.9793     0.4769 -10.442   <0.001 ***
## Strain4 - Strain1 == 0  -4.5863     0.4119 -11.135   <0.001 ***
## Strain5 - Strain1 == 0  -0.3290     0.1735  -1.896   0.4477    
## Strain6 - Strain1 == 0  -0.8283     0.1771  -4.677   <0.001 ***
## Strain7 - Strain1 == 0  -0.5452     0.1755  -3.107   0.0259 *  
## Strain3 - Strain2 == 0  -2.3691     0.4762  -4.975   <0.001 ***
## Strain4 - Strain2 == 0  -1.9760     0.4117  -4.799   <0.001 ***
## Strain5 - Strain2 == 0   2.2812     0.2211  10.320   <0.001 ***
## Strain6 - Strain2 == 0   1.7820     0.2130   8.365   <0.001 ***
## Strain7 - Strain2 == 0   2.0651     0.2178   9.480   <0.001 ***
## Strain4 - Strain3 == 0   0.3930     0.5856   0.671   0.9930    
## Strain5 - Strain3 == 0   4.6503     0.4754   9.781   <0.001 ***
## Strain6 - Strain3 == 0   4.1510     0.4710   8.813   <0.001 ***
## Strain7 - Strain3 == 0   4.4341     0.4735   9.365   <0.001 ***
## Strain5 - Strain4 == 0   4.2573     0.4102  10.379   <0.001 ***
## Strain6 - Strain4 == 0   3.7580     0.4054   9.271   <0.001 ***
## Strain7 - Strain4 == 0   4.0411     0.4080   9.903   <0.001 ***
## Strain6 - Strain5 == 0  -0.4993     0.1769  -2.822   0.0608 .  
## Strain7 - Strain5 == 0  -0.2162     0.1761  -1.228   0.8674    
## Strain7 - Strain6 == 0   0.2831     0.1788   1.584   0.6622    
## ---
## Signif. codes:  0 '***' 0.001 '**' 0.01 '*' 0.05 '.' 0.1 ' ' 1
## (Adjusted p values reported -- single-step method)
```

## Analysis of survival curves and fits

Comparing the Kaplan-Meier survival estimates can be done in different ways. Here i will use non-parametric log-rank testing and parametric survival regression.

### Logrank testing

The below produces all pairwise comparisons of the Kaplan Meier estimate of survival using a logrank test.

```
###Make a table of pairwise chisq pvalues, for the logrank test.
#Based on a post to the R Mailing list by T. Therneau
pw_logrank_test_type <- 0 ###0 for logrank, 1 for peto and peto
pw_logrank <- matrix(0., nlevels(surv_from_DI$Strain),nlevels(surv_from_DI$Strain))
for (i in 1:nlevels(surv_from_DI$Strain)) {
  for (j in (1:nlevels(surv_from_DI$Strain))[-i]) {
    datasubset <- droplevels(subset( surv_from_DI,
      surv_from_DI$Strain %in% (unique(surv_from_DI$Strain))[c(i,j)]))
    temp <- survdiff(Surv(End, Death)~Strain+strata(Batch), data=datasubset, rho=pw_logrank_test_type)
      
    pw_logrank[i,j] <- pchisq(temp$chisq, df=1, lower=F) ##df will always be 1 because this is pairwise
    }
}
colnames(pw_logrank) <- levels(surv_from_DI$Strain)
rownames(pw_logrank) <- levels(surv_from_DI$Strain)
#Make dummy adjustment table
pw_logrank_adjBon <- pw_logrank
#Fill adjusted pvalue table.
for (i in 1:ncol(pw_logrank)) {
  pw_logrank_adjBon[,i] <- cbind(p.adjust(pw_logrank[,i], method="bonferroni"))
}
```

```
stargazer::stargazer(pw_logrank_adjBon,type="html",title="Pairwise Chisq p-values (Bonferroni adjusted)")
```

**Pairwise Chisq p-values (Bonferroni adjusted)**

|  | | | | | | | |
|  | Strain1 | Strain2 | Strain3 | Strain4 | Strain5 | Strain6 | Strain7 |
|  | | | | | | | |
| Strain1 | 0 | 0 | 0 | 0 | 0.112 | 0.00005 | 0.012 |
| Strain2 | 0 | 0 | 0 | 0.00000 | 0 | 0 | 0 |
| Strain3 | 0 | 0 | 0 | 1 | 0 | 0 | 0 |
| Strain4 | 0 | 0.00000 | 1 | 0 | 0 | 0 | 0 |
| Strain5 | 0.112 | 0 | 0 | 0 | 0 | 0.106 | 1 |
| Strain6 | 0.00005 | 0 | 0 | 0 | 0.106 | 0 | 1 |
| Strain7 | 0.012 | 0 | 0 | 0 | 1 | 1 | 0 |
|  | | | | | | | |

### Regressions

Generally a survival regression does not assume proportionality of hazards. A survival regression is fit to a distribution, defined by dist=“”.

```
####Survival Regression###
###This is done using functions from rms.
###psm is a survival::survreg wrapper. but the output is more handle-able for some other functions.
ddist <- datadist(surv_from_DI)
options(datadist="ddist")
psurv_gaus <- psm(Surv(End, Death) ~Strain, data=surv_from_DI, dist="gaussian")
psurv_logistic <- psm(Surv(End, Death) ~Strain, data=surv_from_DI, dist="logistic")
psurv_lnorm <- psm(Surv(End, Death) ~Strain, data=surv_from_DI, dist="lognormal")
psurv_wei <- psm(Surv(End, Death) ~Strain, data=surv_from_DI, dist="weibull")

###Same with survreg()
s_reg_gaus <- survreg(Surv(End, Death) ~Strain, data=surv_from_DI, dist="gaussian")
s_reg_logistic <- survreg(Surv(End, Death) ~Strain, data=surv_from_DI, dist="logistic")
s_reg_lnorm <- survreg(Surv(End, Death) ~Strain, data=surv_from_DI, dist="lognormal")
s_reg_wei <- survreg(Surv(End, Death) ~Strain, data=surv_from_DI, dist="weibull")  

aic.scores.psurv <- rbind(
  extractAIC(psurv_wei),
  extractAIC(psurv_gaus),
  extractAIC(psurv_logistic),
  extractAIC(psurv_lnorm))
###Make useable AIC table
rownames(aic.scores.psurv) <- c("Weibull", "Gaussian", "Logist", "Lognorm")
colnames(aic.scores.psurv) <- c("df", "AIC")
###Call table
```

```
stargazer::stargazer(aic.scores.psurv,type="html",title="AIC Scores")
```

**AIC Scores**

|  | | |
|  | df | AIC |
|  | | |
| Weibull | 8 | 1,399.637 |
| Gaussian | 8 | 1,416.756 |
| Logist | 8 | 1,397.225 |
| Lognorm | 8 | 1,339.207 |
|  | | |

From the table above, the model with the lowest AIC score can be chosen. For this analysis, this is the lognormal model, but this does not have to apply to other experiments. Then, one can inspect that model for significant differences.

```
summary(glht(psurv_lnorm,linfct=mcp(Strain="Tukey")))
```

```
## 
##   Simultaneous Tests for General Linear Hypotheses
## 
## Multiple Comparisons of Means: Tukey Contrasts
## 
## 
## Fit: psm(formula = Surv(End, Death) ~ Strain, data = surv_from_DI, 
##     dist = "lognormal")
## 
## Linear Hypotheses:
##                        Estimate Std. Error z value Pr(>|z|)    
## Strain2 - Strain1 == 0  0.65022    0.04681  13.891  < 0.001 ***
## Strain3 - Strain1 == 0  1.09163    0.06964  15.676  < 0.001 ***
## Strain4 - Strain1 == 0  0.98912    0.06098  16.222  < 0.001 ***
## Strain5 - Strain1 == 0  0.07761    0.04482   1.732  0.58349    
## Strain6 - Strain1 == 0  0.17362    0.04480   3.875  0.00186 ** 
## Strain7 - Strain1 == 0  0.11550    0.04485   2.575  0.12743    
## Strain3 - Strain2 == 0  0.44140    0.06993   6.312  < 0.001 ***
## Strain4 - Strain2 == 0  0.33890    0.06144   5.515  < 0.001 ***
## Strain5 - Strain2 == 0 -0.57261    0.04682 -12.231  < 0.001 ***
## Strain6 - Strain2 == 0 -0.47660    0.04681 -10.182  < 0.001 ***
## Strain7 - Strain2 == 0 -0.53472    0.04683 -11.418  < 0.001 ***
## Strain4 - Strain3 == 0 -0.10251    0.07796  -1.315  0.83893    
## Strain5 - Strain3 == 0 -1.01402    0.06963 -14.563  < 0.001 ***
## Strain6 - Strain3 == 0 -0.91801    0.06964 -13.182  < 0.001 ***
## Strain7 - Strain3 == 0 -0.97612    0.06961 -14.023  < 0.001 ***
## Strain5 - Strain4 == 0 -0.91151    0.06097 -14.951  < 0.001 ***
## Strain6 - Strain4 == 0 -0.81550    0.06098 -13.374  < 0.001 ***
## Strain7 - Strain4 == 0 -0.87361    0.06095 -14.334  < 0.001 ***
## Strain6 - Strain5 == 0  0.09601    0.04482   2.142  0.31697    
## Strain7 - Strain5 == 0  0.03789    0.04487   0.845  0.97912    
## Strain7 - Strain6 == 0 -0.05812    0.04485  -1.296  0.84802    
## ---
## Signif. codes:  0 '***' 0.001 '**' 0.01 '*' 0.05 '.' 0.1 ' ' 1
## (Adjusted p values reported -- single-step method)
```

Again, one can then inspect the different effects, for example using pairwise comparisons of means.

```
pairwise_confint_sreg <- as.data.frame(confint(glht(psurv_lnorm, mcp(Strain = "Tukey")))$confint)
pairwise_confint_sreg$Comparison <- rownames(pairwise_confint_sreg)
###Plot the comparisons, below may not be the most straight-foward way to plot this the way I want it, but it works.
ggplot(pairwise_confint_sreg, aes(x = Comparison, y = Estimate, ymin = lwr, ymax = upr, color = Comparison)) + ###Plot Comparison on x, estimate on y
  scale_x_discrete(limits = rev(levels(as.factor(pairwise_confint$Comparison)))) + ###Rescale x, so the order is inverted 
  geom_errorbar() + geom_point() + ###Draw data
  coord_flip() +  theme(legend.position="none") + xlab("") +###Invert X and Y, hide legend
  ggtitle("Difference in means with 95% Confidence interval") ##Add a title
```

### Plotting of parametric survival regression

It is possible, but not really easy, to plot the curves generated using parametric survival regression. These curves are the result of fitting the data to a distribution in the earlier section. Doing this in a manner that is compatible with ggplot2 requires a nice dataframe. Below is code to generate plots of the KM estimates per batch and the generated fit. This is performed for the four distributions above, and can be adapted to different distributions if necessary.

```
###Step 1, extract the coefficients. These are relative to Strain1 because Strain is treatment contrasted.

for (i in 1:nlevels(surv_DI_fit.df$Strain)) { #For loop through strains
  if(i==1) { #Strain1 is relative to itself, so no change
  coef_wei <- list()
  coef_logistic <- list()
  coef_gaus <- list()
  coef_lnorm <- list()
  coef_wei[i] <- coef(s_reg_wei)[i]
  coef_logistic[i] <- coef(s_reg_logistic)[i]
  coef_gaus[i] <- coef(s_reg_gaus)[i]
  coef_lnorm[i] <- coef(s_reg_lnorm)[i]
  } else { ###Other strains are relative to 1
  coef_wei[i] <- coef(s_reg_wei)[1] + coef(s_reg_wei)[i]
  coef_logistic[i] <- coef(s_reg_logistic)[1] + coef(s_reg_logistic)[i]
  coef_gaus[i] <- coef(s_reg_gaus)[1] + coef(s_reg_gaus)[i]
  coef_lnorm[i] <- coef(s_reg_lnorm)[1] + coef(s_reg_lnorm)[i]
  }
}
##Step 2
####Store the coefficients and the scale in a new data frame, of parameters
### Keep in mind that survreg.distributions$weibull is different from rweibull, hence the difference in names.
sregparams <- data.frame(
  Strain = rep(levels(surv_from_DI$Strain),4 ), #Fill with strains
  scale.wei = exp(unlist(coef_wei)), #weibull fit scale parameters
  scale.logistic = rep(s_reg_logistic$scale, nlevels(surv_from_DI$Strain)), #fill with logis scales
  scale.gaus = rep(s_reg_gaus$scale, nlevels(surv_from_DI$Strain)), #fill with gaus scales
  scale.lnorm = rep(s_reg_lnorm$scale, nlevels(surv_from_DI$Strain)), #fill with lnorm scale
  shape.wei =  rep(1/s_reg_wei$scale, nlevels(surv_from_DI$Strain)), #shape for weibull
  shape.logistic = unlist(coef_logistic), #shape for logistic
  shape.gaus =  unlist(coef_gaus), #shape for gaus
  shape.lnorm =   unlist(coef_lnorm) #shape for lnorm
  )
##Step 3
###Calculate the "daily" value of each curve
for (i in 1:nlevels(surv_DI_fit.df$Strain)){
  if(i==1) {
    wei <- list()
    logis <- list()
    gaus <- list()
    lnorm <- list()
  }
  x <- levels(surv_DI_fit.df$Strain)[i]
  n <- c(1:max(surv_from_DI$End))
  data <- filter(sregparams, Strain==x)
  time <- n
  wei <- cbind(wei, pweibull(
    q=n,
    scale=data$scale.wei,
    shape=data$shape.wei,
    lower.tail=FALSE))
  logis <- cbind(logis,plogis(
    q=n,
    scale=data$scale.logistic,
    location=data$shape.logistic,
    lower.tail=FALSE  ))
  gaus <- cbind(gaus,pnorm(
   q=n,
   sd=data$scale.gaus,
   mean=data$shape.gaus,
   lower.tail = F))
  lnorm <- cbind(lnorm, plnorm(
    q=n,
    sd=data$scale.lnorm,
    mean=data$shape.lnorm,
    lower.tail=F))
}

##Step 4
###Put all the curves into a data.frame that contains information on "time" and also "Strain", for compatibility with other data.frames
sreg_curves <- data.frame(
  wei.sreg = cbind(unlist(wei)),
  logis.sreg = cbind(unlist(logis)),
  gaus.sreg = cbind(unlist(gaus)),
  lnorm.sreg = cbind(unlist(lnorm)),
  Strain = rep(unlist(levels(surv_DI_fit.df$Strain)),each=max(surv_from_DI$End)),
  time = rep(c(1:max(surv_from_DI$End)), nlevels(surv_DI_fit.df$Strain))
)
##Step 5
###Turn that data.frame into a long data.frame (not used here but could be handy.)
sreg_long <- sreg_curves %>% gather(., key="Distribution",values = c(lnorm.sreg, wei.sreg,gaus.sreg,logis.sreg) )
sreg_long$Distribution <- as.factor(sreg_long$Distribution)
##Levels: gaus.sreg lnorm.sreg logis.sreg wei.sreg
levels(sreg_long$Distribution) <- c("Gaussian","Lognormal","Loglogistic","Weibull")
```

Now, these can be plotted and inspected visually..

```
###Plot of KM+Weibull
  ggplot(surv_DI_fit.df, aes(time, surv, colour = Strain)) +
  geom_step(aes(linetype=Batch)) +
  geom_line(data=sreg_curves,aes(y=wei.sreg),color="black") +
  facet_wrap(~Strain) + 
  ggtitle("Kaplan-Meier estimates and fit to\nWeibull distribution")
```

```
###Plot of KM+Logistic
  ggplot(surv_DI_fit.df, aes(time, surv, colour = Strain)) +
    geom_step(aes(linetype=Batch)) +
    geom_line(data=sreg_curves,aes(y=logis.sreg),color="black") +
    facet_wrap(~Strain) + 
    ggtitle("Kaplan-Meier estimates and fit to\n Logistic distribution")
```

```
###Plot of KM+Gaussian  
  ggplot(surv_DI_fit.df, aes(time, surv, colour = Strain)) +
    geom_step(aes(linetype=Batch)) +
    geom_line(data=sreg_curves,aes(y=gaus.sreg),color="black") +
    facet_wrap(~Strain) + 
    ggtitle("Kaplan-Meier estimates and fit to\nGaussian distribution")
```

```
###Plot of KM+Lognormal  
  ggplot(surv_DI_fit.df, aes(time, surv, colour = Strain)) +
    geom_step(aes(linetype=Batch)) +
    geom_line(data=sreg_curves,aes(y=lnorm.sreg),color="black") +
    facet_wrap(~Strain) + 
    ggtitle("Kaplan-Meier estimates and fit to\nLognormal distribution")
```

# Comparison of analyis method results.

An inherent question when analyzing data, is which analysis produced which result and why. Below, the outputs from the three major analysis performed are printed, so they can be compared.

```
summary(auc_lmer)
```

```
## Linear mixed model fit by REML t-tests use Satterthwaite approximations
##   to degrees of freedom [lmerMod]
## Formula: AUC ~ Strain + (1 | Batch)
##    Data: auc_df
## 
## REML criterion at convergence: 2814.1
## 
## Scaled residuals: 
##     Min      1Q  Median      3Q     Max 
## -4.6437 -0.3615  0.0894  0.4529  3.0154 
## 
## Random effects:
##  Groups   Name        Variance Std.Dev.
##  Batch    (Intercept)  2.92    1.709   
##  Residual             21.69    4.657   
## Number of obs: 476, groups:  Batch, 5
## 
## Fixed effects:
##               Estimate Std. Error       df t value Pr(>|t|)    
## (Intercept)    23.5642     0.9504   8.2000  24.793 5.43e-09 ***
## StrainStrain2 -15.9816     0.7987 465.0000 -20.009  < 2e-16 ***
## StrainStrain3 -15.2500     0.7987 465.0000 -19.093  < 2e-16 ***
## StrainStrain4  -7.6324     0.7987 465.0000  -9.556  < 2e-16 ***
## StrainStrain5  -1.2059     0.7987 465.0000  -1.510   0.1318    
## StrainStrain6  -3.1581     0.7987 465.0000  -3.954 8.89e-05 ***
## StrainStrain7  -2.6691     0.7987 465.0000  -3.342   0.0009 ***
## ---
## Signif. codes:  0 '***' 0.001 '**' 0.01 '*' 0.05 '.' 0.1 ' ' 1
## 
## Correlation of Fixed Effects:
##             (Intr) StrnS2 StrnS3 StrnS4 StrnS5 StrnS6
## StrainStrn2 -0.420                                   
## StrainStrn3 -0.420  0.500                            
## StrainStrn4 -0.420  0.500  0.500                     
## StrainStrn5 -0.420  0.500  0.500  0.500              
## StrainStrn6 -0.420  0.500  0.500  0.500  0.500       
## StrainStrn7 -0.420  0.500  0.500  0.500  0.500  0.500
```

```
summary(disease_lmer)
```

```
## Linear mixed model fit by REML t-tests use Satterthwaite approximations
##   to degrees of freedom [lmerMod]
## Formula: DI ~ Strain + Strain:DPI + (1 | Batch) + (1 | subject)
##    Data: di_long_useful
## 
## REML criterion at convergence: 4885.9
## 
## Scaled residuals: 
##     Min      1Q  Median      3Q     Max 
## -3.4325 -0.3999  0.0021  0.3596  3.2849 
## 
## Random effects:
##  Groups   Name        Variance Std.Dev.
##  subject  (Intercept) 1.0381   1.0189  
##  Batch    (Intercept) 0.1678   0.4097  
##  Residual             0.4169   0.6457  
## Number of obs: 1901, groups:  subject, 476; Batch, 5
## 
## Fixed effects:
##                     Estimate Std. Error         df t value Pr(>|t|)    
## (Intercept)         -2.15059    0.26604   15.30000  -8.084 6.48e-07 ***
## StrainStrain2       -1.36731    0.32617 1553.00000  -4.192 2.92e-05 ***
## StrainStrain3        3.03661    0.24256  898.40000  12.519  < 2e-16 ***
## StrainStrain4        4.06498    0.24253  897.90000  16.761  < 2e-16 ***
## StrainStrain5       -1.45363    0.28979 1445.50000  -5.016 5.92e-07 ***
## StrainStrain6       -1.20492    0.28924 1405.90000  -4.166 3.29e-05 ***
## StrainStrain7       -1.06011    0.29048 1342.10000  -3.649 0.000273 ***
## StrainStrain1:DPI    0.84087    0.02675 1257.30000  31.434  < 2e-16 ***
## StrainStrain2:DPI    0.54726    0.02588 1611.70000  21.144  < 2e-16 ***
## StrainStrain3:DPI    0.02389    0.01133 1201.70000   2.108 0.035206 *  
## StrainStrain4:DPI    0.01727    0.01129 1201.40000   1.529 0.126461    
## StrainStrain5:DPI    1.06117    0.03181 1303.10000  33.363  < 2e-16 ***
## StrainStrain6:DPI    0.93431    0.02893 1416.40000  32.295  < 2e-16 ***
## StrainStrain7:DPI    0.92674    0.03029 1575.40000  30.597  < 2e-16 ***
## ---
## Signif. codes:  0 '***' 0.001 '**' 0.01 '*' 0.05 '.' 0.1 ' ' 1
```

```
## 
## Correlation matrix not shown by default, as p = 14 > 12.
## Use print(x, correlation=TRUE)  or
##   vcov(x)     if you need it
```

```
summary(psurv_lnorm)
```

```
##              Effects              Response : Surv(End, Death) 
## 
##  Factor                   Low High Diff. Effect   S.E.     Lower 0.95
##  Strain - Strain2:Strain1 1   2    NA    0.650220 0.046809  0.558240 
##   Survival Time Ratio     1   2    NA    1.916000       NA  1.747600 
##  Strain - Strain3:Strain1 1   3    NA    1.091600 0.069639  0.954780 
##   Survival Time Ratio     1   3    NA    2.979100       NA  2.598100 
##  Strain - Strain4:Strain1 1   4    NA    0.989120 0.060975  0.869300 
##   Survival Time Ratio     1   4    NA    2.688900       NA  2.385200 
##  Strain - Strain5:Strain1 1   5    NA    0.077608 0.044819 -0.010464 
##   Survival Time Ratio     1   5    NA    1.080700       NA  0.989590 
##  Strain - Strain6:Strain1 1   6    NA    0.173620 0.044804  0.085577 
##   Survival Time Ratio     1   6    NA    1.189600       NA  1.089300 
##  Strain - Strain7:Strain1 1   7    NA    0.115500 0.044854  0.027363 
##   Survival Time Ratio     1   7    NA    1.122400       NA  1.027700 
##  Upper 0.95
##  0.74220   
##  2.10060   
##  1.22850   
##  3.41600   
##  1.10890   
##  3.03110   
##  0.16568   
##  1.18020   
##  0.26166   
##  1.29910   
##  0.20364   
##  1.22590
```

An easier comparisons might be accomplished with the compact letter display.

```
cld(glht(auc_lmer, linfct=mcp(Strain="Tukey")))
```

```
## Strain1 Strain2 Strain3 Strain4 Strain5 Strain6 Strain7 
##     "d"     "a"     "a"     "b"    "cd"     "c"     "c"
```

```
#Compact letters for lmerTest objects are a little tricky. This solution comes from the rcompanion. Mangiafico, S.S. 2015. An R Companion for the Handbook of Biological Statistics, version 1.3.0. 
### Extract lsmeans table


cldList(comparison = Comparison,
        p.value    = p.adjust(lmerlsm$'p-value',
                       method =  "bonferroni")   ,
        threshold = 0.05)
```

```
##     Group Letter MonoLetter
## 1 Strain1      a      a    
## 2 Strain2      b       b   
## 3 Strain3      c        c  
## 4 Strain4      d         d 
## 5 Strain5     ae      a   e
## 6 Strain6     de         de
## 7 Strain7    ade      a  de
```

```
cld(glht(srv_coxph,linfct=mcp(Strain="Tukey")))
```

```
## Strain1 Strain2 Strain3 Strain4 Strain5 Strain6 Strain7 
##     "a"     "c"     "d"     "d"    "ab"     "b"     "b"
```

```
cld(glht(psurv_lnorm,linfct=mcp(Strain="Tukey")))
```

```
## Strain1 Strain2 Strain3 Strain4 Strain5 Strain6 Strain7 
##     "a"     "c"     "d"     "d"    "ab"     "b"    "ab"
```

```
cld(glht(cme,linfct=mcp(Strain="Tukey")))
```

```
## Strain1 Strain2 Strain3 Strain4 Strain5 Strain6 Strain7 
##     "a"     "c"     "d"     "d"    "ab"     "b"     "b"
```

# Session Info

```
sessionInfo()
```

```
## R version 3.3.3 (2017-03-06)
## Platform: x86_64-w64-mingw32/x64 (64-bit)
## Running under: Windows 10 x64 (build 14393)
## 
## locale:
## [1] LC_COLLATE=English_United States.1252 
## [2] LC_CTYPE=English_United States.1252   
## [3] LC_MONETARY=English_United States.1252
## [4] LC_NUMERIC=C                          
## [5] LC_TIME=English_United States.1252    
## 
## attached base packages:
## [1] stats     graphics  grDevices utils     datasets  methods   base     
## 
## other attached packages:
##  [1] coxme_2.2-5      bdsmatrix_1.3-2  rms_5.1-0        SparseM_1.74    
##  [5] Hmisc_4.0-2      Formula_1.2-1    lattice_0.20-34  survcomp_1.24.0 
##  [9] prodlim_1.5.9    modelr_0.1.0     rcompanion_1.5.0 multcomp_1.4-6  
## [13] TH.data_1.0-8    MASS_7.3-45      survival_2.40-1  mvtnorm_1.0-5   
## [17] lmerTest_2.0-33  lme4_1.1-12      Matrix_1.2-8     MESS_0.4-3      
## [21] geepack_1.2-1    stringr_1.2.0    purrr_0.2.2      readr_1.0.0     
## [25] tibble_1.2       ggplot2_2.2.1    tidyverse_1.1.1  dplyr_0.5.0     
## [29] broom_0.4.2      tidyr_0.6.1     
## 
## loaded via a namespace (and not attached):
##  [1] minqa_1.2.4          colorspace_1.3-2     modeltools_0.2-21   
##  [4] rprojroot_1.2        htmlTable_1.9        base64enc_0.1-3     
##  [7] mc2d_0.1-18          MatrixModels_0.4-1   manipulate_1.0.1    
## [10] lubridate_1.6.0      coin_1.1-3           xml2_1.1.1          
## [13] codetools_0.2-15     splines_3.3.3        mnormt_1.5-5        
## [16] knitr_1.15.1         SuppDists_1.1-9.4    ade4_1.7-5          
## [19] ordinal_2015.6-28    jsonlite_1.3         nloptr_1.0.4        
## [22] pbkrtest_0.4-7       cluster_2.0.5        httr_1.2.1          
## [25] backports_1.0.5      assertthat_0.1       lazyeval_0.2.0      
## [28] acepack_1.4.1        htmltools_0.3.5      quantreg_5.29       
## [31] tools_3.3.3          gtable_0.2.0         reshape2_1.4.2      
## [34] Rcpp_0.12.9          nlme_3.1-131         stargazer_5.2       
## [37] psych_1.6.12         lmtest_0.9-35        hermite_1.1.1       
## [40] rvest_0.3.2          polspline_1.1.12     zoo_1.7-14          
## [43] scales_0.4.1         miscTools_0.6-22     hms_0.3             
## [46] parallel_3.3.3       sandwich_2.3-4       expm_0.999-1        
## [49] RColorBrewer_1.1-2   yaml_2.1.14          BSDA_1.01           
## [52] gridExtra_2.2.1      EMT_1.1              rpart_4.1-10        
## [55] rmeta_2.16           RVAideMemoire_0.9-63 reshape_0.8.6       
## [58] latticeExtra_0.6-28  stringi_1.1.2        ucminf_1.1-4        
## [61] nortest_1.0-4        checkmate_1.8.2      boot_1.3-18         
## [64] lava_1.4.7           evaluate_0.10        survivalROC_1.0.3   
## [67] htmlwidgets_0.8      labeling_0.3         plyr_1.8.4          
## [70] magrittr_1.5         R6_2.2.0             DescTools_0.99.19   
## [73] multcompView_0.1-7   bootstrap_2017.2     DBI_0.5-1           
## [76] haven_1.0.0          foreign_0.8-67       mgcv_1.8-17         
## [79] nnet_7.3-12          car_2.1-4            WRS2_0.9-1          
## [82] KernSmooth_2.23-15   rmarkdown_1.3        maxLik_1.3-4        
## [85] grid_3.3.3           readxl_0.1.1         data.table_1.10.4   
## [88] forcats_0.2.0        digest_0.6.12        stats4_3.3.3        
## [91] munsell_0.4.3
```

# Appendix; Manuscript figures

The below was used to generate figures for the main text.

```
####This script is used to generate  figures for the Manuscript####
#It requires that everything from the rmd file is available through the environment,
#in practical terms, that the markdown script was run through the R console.

#### Fig1 ####
##generate a visual display of 
#a) raw data.
#b) AUDPC 
#c) Linear fit and 
#d) survival curve

library("grid")

Fig1a <- ggplot(data=di_long) +
                  geom_jitter(aes(x=DPI, y=DI, color=Strain, shape=Batch)) +
                  #geom_segment(aes(x=0, xend=max(DPI)+0.5,y=cutoff, yend=cutoff), linetype="dotted") +
                  labs(x = "Days post infection", y = "Disease Index", title="a) Scatterplot of all disease indices") +
                  coord_cartesian(xlim=c(2,11))+
  theme_bw()
                  
Fig1b <-   ggplot(data=filter(di_summary, Batch=="B-I")) +
                  aes(x=DPI,y=mean, color=Strain,fill=Strain) +
                  geom_area(alpha=0.10, position="identity") +
                  labs(x = "Days post infection", y = "Avg. Disease Index", title="b) Area under the disease progression curve (Batch B-I)")+
  coord_cartesian(xlim=c(2,11))+
  theme_bw() #+ theme(legend.position="none")


Fig1c <- 
  ggplot(data=filter(di_long), aes(x=DPI,y=DI)) +
  geom_smooth(aes(y=lmer.pred,color=Strain),method="lm", se= F) +
  coord_cartesian(ylim=c(-0.5,4.5),xlim=c(2,11)) +
  #theme(legend.position="none") +
  labs(x = "Days post infection", y = "Disease Index", 
       title="c) Estimated disease development")+ #Labels
  theme_bw() 

Fig1d <-  ggplot(data=filter(surv_DI_fit.df),aes(x=time, y=surv, color=Strain)) + 
  geom_step(aes(linetype=Batch),alpha=1) + geom_line(data=sreg_curves,aes(y=lnorm.sreg ,color=Strain), size=1.1, alpha=0.6) +
  labs(x="Days post infection", y="Estimated survival", title="d) Kaplan-Meier survival estimates and fits to lognormal distribution") +
  coord_cartesian(xlim=c(2,11)) +
  theme_bw()

dev.off()
pushViewport(viewport(layout= grid.layout(nrow=2, ncol=2)))
print(Fig1a, vp = viewport(layout.pos.row = 1, layout.pos.col = 1))
print(Fig1b, vp = viewport(layout.pos.row = 1, layout.pos.col = 2))
print(Fig1c, vp = viewport(layout.pos.row = 2, layout.pos.col = 1))
print(Fig1d, vp = viewport(layout.pos.row = 2, layout.pos.col = 2))


###Figure 1 done####

####clear device
dev.off()

#### Fig 2 #####
##Figure on AUDPC
###For the purpose of visualization calculate summaries across all replicates
di_summary.nobatch <- di_long %>% group_by(Plant, Strain, DPI) %>% summarise(mean(DI),sd(DI),sd(DI)/sqrt(length(DI)))
colnames(di_summary.nobatch) <- c("Plant", "Strain", "DPI", "mean", "sd", "se")
####Fig2a: Plot the actual areas per strain
Fig2a <- ggplot(di_summary.nobatch) +
  aes(x=DPI,y=mean,color=Strain) +
  geom_area(aes(fill=Strain), position="identity",alpha=0.15) +
  geom_errorbar(aes(x=DPI, ymax=mean+se,ymin=mean-se))+
  facet_wrap(~Strain) +
  labs(x = "Days post infection", y = "Avg. Disease Index") +
  theme(legend.position="none") +
  ggtitle("a) Disease progression curves per strain")+
  theme_bw()

###Some extra scripting to make the mean and CI plot.
auc_CI <- as.data.frame(tidy(confint(auc_lmer)))
auc_CI <- auc_CI[3:9,] ##Drop sig01, sigma
auc_CI$Strain <- levels(auc_df$Strain)
###Mean relative to Strain1 (except strain1 that one is absolute)

for (i in 1:nrow(auc_CI)){
  if (i==1){
    auc_CI$mean[i] <- mean(c(auc_CI$X2.5..[i],auc_CI$X97.5..[i]))
    auc_CI$upr[i] <- auc_CI$X97.5..[i]
    auc_CI$lwr[i] <- auc_CI$X2.5..[i]
  } else {
    auc_CI$mean[i] <- c(auc_CI$mean[1]+mean(c(auc_CI$X2.5..[i],auc_CI$X97.5..[i])))
    auc_CI$upr[i] <- c(auc_CI$mean[1]+auc_CI$X97.5..[i])
    auc_CI$lwr[i] <- c(auc_CI$mean[1]+auc_CI$X2.5..[i])
  }
}

#####Fig2b meanCI the areas, and add each individual area as jitter; symbols in jitter by batch
Fig2b <- ####Generate meanCI of AUDCP with significance letters
  ggplot(aes(x=Strain, y=AUC, color=Strain),data=auc_df) + ###Plot the auc_df
  geom_crossbar(data = auc_CI, aes(x = Strain, y = mean, ymin = lwr, ymax = upr,fill=Strain), alpha=0.3) +
  geom_jitter(aes(shape=Batch)) + ###with jitter overplotted, symbol shape defined by batch
  geom_text(aes(x=Strain, y=-3, label=Letter),color="black", data=auc_cld) + ###Get the letters from auc_cld 
  #and write those to position y=-3
  labs(y="AUDPC") + #Y-Axis label
  ggtitle("b) AUDPC raw values and estimated means with 95% CI")+
  theme_bw() #Title 

###Arrage figures via Viewports
pushViewport(viewport(layout= grid.layout(nrow=1, ncol=2)))
print(Fig2a, vp = viewport(layout.pos.row = 1, layout.pos.col = 1))
print(Fig2b, vp = viewport(layout.pos.row = 1, layout.pos.col = 2))

###End of Fig2####
####clear device
dev.off()

#### Fig 3 ####
###Use only 6 Strains to make the facetting more usable, its kind of not nice when the number of plots is a prime..

#Omit Strain4, it is not super interesting, its basically strain3 +1
Fig3Strains <- c("Strain1","Strain2","Strain3","Strain5","Strain6","Strain7")

#predict uncensored
di_long <- add_predictions(di_long, lmer(DI ~ Strain + Strain:DPI + (1 | subject) + (1 | Batch), data = di_long), var="lmer.pred.no.cens")

###3A: Linear fits
F.3A <- ggplot(data=filter(di_long, Strain %in% Fig3Strains), aes(x=DPI,y=DI)) +
  geom_jitter(aes(group=DPI),data=filter(di_long, Strain %in% Fig3Strains, Useful=="No", Strain %in% Fig3Strains),color="red",alpha=0.3) +
  geom_jitter(aes(group=DPI),data=filter(di_long, Strain %in% Fig3Strains, Useful=="Yes", Strain %in% Fig3Strains),color="darkgrey",alpha=0.5)+
  stat_summary(aes(y=lmer.pred),color="black", fun.y=mean , geom = "line" , alpha=0.6) +
  stat_summary(aes(y=lmer.pred.no.cens), fun.y=mean,color="red" , geom = "line" , alpha=0.6) +
  coord_cartesian(ylim=c(-0.5,4.5)) +
  facet_wrap(~Strain)+
  labs(x = "Days post infection", y = "Disease Index") + #Labels
  ggtitle("b) Raw data and linear fit for individual strains averaged across batches")+
  theme_bw()

###3B

F.3B <- ggplot(filter(di_summary, Strain %in% Fig3Strains), ###Use Only Batch A (too busy otherwise)
  aes(x=DPI,y=mean,color=Strain)) + ###Color by Strain, specify x and y.
  stat_summary(aes(fill=Strain), position="identity",alpha=0.15,fun.y=mean,geom="area") + ###Area plot, colored by Strain
  ##geom_errorbar(aes(x=DPI, ymax=mean+se, ymin=mean-se), size=0.25)+ ###This line adds SE. 
  ##geom_errorbar(aes(x=DPI, ymax=mean+sd, ymin=mean-sd), size=0.25)+ ###This line adds SD. 
  stat_summary(aes(y=lmer.pred, x=DPI), color="black", filter(di_long, Strain %in% Fig3Strains), fun.y = mean, geom="line") +
  stat_summary(aes(y=lmer.pred.no.cens, x=DPI), color="red", filter(di_long, Strain %in% Fig3Strains), fun.y = mean, geom="line") +
  facet_wrap(~Strain) +###One plot per strain 
  coord_cartesian(ylim=c(-0.5,4.5)) +
  labs(x = "Days post infection", y = "Disease Index") + #Labels
  ggtitle("a) Disease Areas and linear fits per strain averaged across batches")+ #Title+
  theme_bw() +
  theme(legend.position="none")


pushViewport(viewport(layout= grid.layout(nrow=1, ncol=2)))
print(F.3B, vp = viewport(layout.pos.row = 1, layout.pos.col = 1))
print(F.3A, vp = viewport(layout.pos.row = 1, layout.pos.col = 2))

###End of Fig3####
####clear device
dev.off()

#### Fig 4 ####

##Display MCP + CI from AUDPC and DISEASE_LMER
##Calculations are done in the Rmd and available through the global environment

gg_PWCI_AUDPC <- ggplot(pairwise_confint_AUDPC_lmer, aes(x = Comparison, y = Estimate, ymin = lwr, ymax = upr, color = abs(Estimate))) + ###Plot Comparison on x, estimate on y
  scale_x_discrete(limits = rev(levels(as.factor(pairwise_confint_AUDPC_lmer$Comparison)))) + ###Rescale x, so the order is inverted 
  geom_errorbar() + geom_point() + ###Draw data
  coord_flip() +  xlab("") +###Invert X and Y, hide xlab
  geom_hline(yintercept=0,linetype=2) + ###Horizontal because the plot is flipped...
  ggtitle("a) Difference in estimated means of the AUDPC")+ ##Add a title+
  theme_bw()+
  theme(legend.position="none") #No legend.

gg_icep_CI <- ggplot(confint_icep, aes(x = Strain, y = Estimate, ymin = lwr, ymax = upr, color = abs(Estimate))) + ###Plot Comparison on x, estimate on y
  scale_x_discrete(limits = rev(levels(as.factor(confint_icep$Strain)))) + ###Rescale x, so the order is inverted 
  geom_errorbar() + geom_point() + ###Draw data
  coord_flip()  + xlab("") +###Invert X and Y, hide legend
  ggtitle("b) Intercepts with 95%CI") + theme_bw()+
  theme(legend.position="none") #No legend.

gg_slopes_CI <- ggplot(confint_slopes, aes(x = Strain, y = Estimate, ymin = lwr, ymax = upr, color = abs(Estimate))) + ###Plot Comparison on x, estimate on y
  scale_x_discrete(limits = rev(levels(as.factor(confint_slopes$Strain)))) + ###Rescale x, so the order is inverted 
  geom_errorbar() + geom_point() + ###Draw data
  coord_flip() +  theme(legend.position="none") + xlab("") +###Invert X and Y, hide legend
  ggtitle("c) Slopes with 95%CI") + theme_bw()+
  theme(legend.position="none") #No legend.

pushViewport(viewport(layout= grid.layout(nrow=2, ncol=2)))
print(gg_PWCI_AUDPC, vp = viewport(layout.pos.row = c(1,2), layout.pos.col = 1))
print(gg_icep_CI, vp = viewport(layout.pos.row = 1, layout.pos.col = 2))
print(gg_slopes_CI, vp = viewport(layout.pos.row = 2, layout.pos.col = 2))
###End of Fig4####
####clear device
dev.off()


###Fig 5 ####

##Should display effects of bad cutoffing


###Because i need to make multiple survfit dataframes, i have converted the script used in the other sections into a function, hopefully this makes it a little easier to follow. 
###Working with multistrata survfits is a little annoying, and breaking the strata back into the predictors is not part of this fuction.
###I would hope that at some point after this was written, a proper survfit tidier has been included into broom.
survfit_to_df <- function(x) {
  strata_dummy <-NULL
  for(i in 1:length(x$strata)){
    # add vector for one strata according to number of rows of strata
    strata_dummy <- c(strata_dummy, rep(names(x$strata)[i], x$strata[i]))
  }
  #make x.df from x..
  x.df <- data.frame( 
    time = x$time, 
    n.risk = x$n.risk, 
    n.event = x$n.event, 
    surv = x$surv, 
    strata = strata_dummy, 
    upper = x$upper, 
    lower = x$lower 
  ) 
  zeros <- data.frame(time = 0, surv = 1, strata = names((x$strata)), 
                      upper = 1, lower = 1)
  
  x.df <- plyr::rbind.fill(zeros, x.df) 
  rm(strata_dummy)
  rm(zeros)
  return(x.df)
}


####Generate intermediate survival tables#####
surv_table1 <- data.frame(Subject=disease_index$subject,
                          Strain=disease_index$Strain,
                          Plant=disease_index$Plant,
                          Batch=disease_index$Batch)

###Fill survival table based on the di_long table. This generates warnings. These can be ignored and come from the min()
cutoff <- c(1)
for (i in 1:max(di_long$subject)) { #Go by subject
  dummy <- di_long[di_long$subject==i,] #generate dummy for the subject
  if (is.infinite(min(dummy$DPI[which(dummy$DI >= cutoff)]))) { #If none of the DI is greater than the cutoff (this is where warnings are generated, min on an empty object returns infinite!)
    surv_table1$End[i] <- max(dummy$DPI) #Generate a  observation, censoring at the maximum DPI recorded
    surv_table1$Death[i] <- 0 #Still alive, because it did not pass the cutoff
  } else { #If more than zero DI are greater than the cutoff
    surv_table1$End[i] <- min(dummy$DPI[which(dummy$DI >= cutoff)]) #Use the lowest DPI where condition is met
    surv_table1$Death[i] <- 1 #record as dead
  }
}

surv_table2 <- data.frame(Subject=disease_index$subject,
                         Strain=disease_index$Strain,
                         Plant=disease_index$Plant,
                         Batch=disease_index$Batch)
###Fill survival table based on the di_long table. This generates warnings. These can be ignored and come from the min()
cutoff <- c(2)
for (i in 1:max(di_long$subject)) { #Go by subject
  dummy <- di_long[di_long$subject==i,] #generate dummy for the subject
  if (is.infinite(min(dummy$DPI[which(dummy$DI >= cutoff)]))) { #If none of the DI is greater than the cutoff (this is where warnings are generated, min on an empty object returns infinite!)
    surv_table2$End[i] <- max(dummy$DPI) #Generate a  observation, censoring at the maximum DPI recorded
    surv_table2$Death[i] <- 0 #Still alive, because it did not pass the cutoff
  } else { #If more than zero DI are greater than the cutoff
    surv_table2$End[i] <- min(dummy$DPI[which(dummy$DI >= cutoff)]) #Use the lowest DPI where condition is met
    surv_table2$Death[i] <- 1 #record as dead
  }
}


surv_table3 <- data.frame(Subject=disease_index$subject,
                          Strain=disease_index$Strain,
                          Plant=disease_index$Plant,
                          Batch=disease_index$Batch)
###Fill survival table based on the di_long table. This generates warnings. These can be ignored and come from the min()
cutoff <- c(3)
for (i in 1:max(di_long$subject)) { #Go by subject
  dummy <- di_long[di_long$subject==i,] #generate dummy for the subject
  if (is.infinite(min(dummy$DPI[which(dummy$DI >= cutoff)]))) { #If none of the DI is greater than the cutoff (this is where warnings are generated, min on an empty object returns infinite!)
    surv_table3$End[i] <- max(dummy$DPI) #Generate a  observation, censoring at the maximum DPI recorded
    surv_table3$Death[i] <- 0 #Still alive, because it did not pass the cutoff
  } else { #If more than zero DI are greater than the cutoff
    surv_table3$End[i] <- min(dummy$DPI[which(dummy$DI >= cutoff)]) #Use the lowest DPI where condition is met
    surv_table3$Death[i] <- 1 #record as dead
  }
}


surv_table4 <- data.frame(Subject=disease_index$subject,
                          Strain=disease_index$Strain,
                          Plant=disease_index$Plant,
                          Batch=disease_index$Batch)
###Fill survival table based on the di_long table. This generates warnings. These can be ignored and come from the min()
cutoff <- c(4)
for (i in 1:max(di_long$subject)) { #Go by subject
  dummy <- di_long[di_long$subject==i,] #generate dummy for the subject
  if (is.infinite(min(dummy$DPI[which(dummy$DI >= cutoff)]))) { #If none of the DI is greater than the cutoff (this is where warnings are generated, min on an empty object returns infinite!)
    surv_table4$End[i] <- max(dummy$DPI) #Generate a  observation, censoring at the maximum DPI recorded
    surv_table4$Death[i] <- 0 #Still alive, because it did not pass the cutoff
  } else { #If more than zero DI are greater than the cutoff
    surv_table4$End[i] <- min(dummy$DPI[which(dummy$DI >= cutoff)]) #Use the lowest DPI where condition is met
    surv_table4$Death[i] <- 1 #record as dead
  }
}

###Make survfits with the four survival tables..
surv_DI_fit1 <- with(surv_table1, survfit(Surv(End, Death) ~Strain+Batch))
surv_DI_fit2 <- with(surv_table2, survfit(Surv(End, Death) ~Strain+Batch))
surv_DI_fit3 <- with(surv_table3, survfit(Surv(End, Death) ~Strain+Batch))
surv_DI_fit4 <- with(surv_table4, survfit(Surv(End, Death) ~Strain+Batch))
###Make survfits into surv_df
surv_DI_fit1.df <- survfit_to_df(surv_DI_fit1)
surv_DI_fit2.df <- survfit_to_df(surv_DI_fit2)
surv_DI_fit3.df <- survfit_to_df(surv_DI_fit3)
surv_DI_fit4.df <- survfit_to_df(surv_DI_fit4)

###However the strata field still needs to be split manually....

surv_DI_fit1.df$Batch <- as.factor( str_split_fixed(
  matrix( nrow=length(surv_DI_fit1.df$strata),ncol=2, unlist(strsplit(as.character(surv_DI_fit1.df$strata),", ")), byrow=T )[,2],"=",2)[,2])
surv_DI_fit1.df$Strain <- as.factor( str_split_fixed(
  matrix( nrow=length(surv_DI_fit1.df$strata),ncol=2, unlist(strsplit(as.character(surv_DI_fit1.df$strata),", ")), byrow=T )[,1],"=",2)[,2])
surv_DI_fit2.df$Batch <- as.factor( str_split_fixed(
  matrix( nrow=length(surv_DI_fit2.df$strata),ncol=2, unlist(strsplit(as.character(surv_DI_fit2.df$strata),", ")), byrow=T )[,2],"=",2)[,2])
surv_DI_fit2.df$Strain <- as.factor( str_split_fixed(
  matrix( nrow=length(surv_DI_fit2.df$strata),ncol=2, unlist(strsplit(as.character(surv_DI_fit2.df$strata),", ")), byrow=T )[,1],"=",2)[,2])
surv_DI_fit3.df$Batch <- as.factor( str_split_fixed(
  matrix( nrow=length(surv_DI_fit3.df$strata),ncol=2, unlist(strsplit(as.character(surv_DI_fit3.df$strata),", ")), byrow=T )[,2],"=",2)[,2])
surv_DI_fit3.df$Strain <- as.factor( str_split_fixed(
  matrix( nrow=length(surv_DI_fit3.df$strata),ncol=2, unlist(strsplit(as.character(surv_DI_fit3.df$strata),", ")), byrow=T )[,1],"=",2)[,2])
surv_DI_fit4.df$Batch <- as.factor( str_split_fixed(
  matrix( nrow=length(surv_DI_fit4.df$strata),ncol=2, unlist(strsplit(as.character(surv_DI_fit4.df$strata),", ")), byrow=T )[,2],"=",2)[,2])
surv_DI_fit4.df$Strain <- as.factor( str_split_fixed(
  matrix( nrow=length(surv_DI_fit4.df$strata),ncol=2, unlist(strsplit(as.character(surv_DI_fit4.df$strata),", ")), byrow=T )[,1],"=",2)[,2])

###End of intermediate table generation####

####PlotZone####
Fig5a <- ggplot(data=filter(surv_DI_fit1.df, Batch=="A   ", Strain %in% c("Strain1","Strain3", "Strain4") ), aes(time, surv, colour = Strain)) + 
  geom_step(aes(y = surv*100),alpha=0.75) +
  ggtitle("a) Event defined as DI >=1") +
  theme_bw() +
  labs(x = "Days post infection", y = "Percentage of plants alive") + 
  theme(legend.position="none")

Fig5b <- ggplot(data=filter(surv_DI_fit2.df, Batch=="A   ", Strain %in% c("Strain1","Strain3", "Strain4") ), aes(time, surv, colour = Strain)) + 
  geom_step(aes(y = surv*100),alpha=0.75) +
  ggtitle("b) Event defined as DI >= 2") +
  theme_bw() +
  labs(x = "Days post infection", y = "Percentage of plants alive") + 
  theme(legend.position="none")

Fig5c <- ggplot(data=filter(surv_DI_fit3.df, Batch=="A   ", Strain %in% c("Strain1","Strain3", "Strain4") ), aes(time, surv, colour = Strain)) + 
  geom_step(aes(y = surv*100),alpha=0.75) +
  ggtitle("c) Event defined as DI >= 3") +
  theme_bw() +
  labs(x = "Days post infection", y = "Percentage of plants alive") + 
  theme(legend.position="none")

Fig5d <- ggplot(data=filter(surv_DI_fit4.df, Batch=="A   ", Strain %in% c("Strain1","Strain3", "Strain4") ), aes(time, surv, colour = Strain)) + 
  geom_step(aes(y = surv*100),alpha=0.75) +
  ggtitle("d) Event defined as DI >= 4") +
  theme_bw() +
  labs(x = "Days post infection", y = "Percentage of plants alive") + 
  theme(legend.position="none")

pushViewport(viewport(layout= grid.layout(nrow=2, ncol=2)))
print(Fig5a, vp = viewport(layout.pos.row = 1, layout.pos.col = 1))
print(Fig5b, vp = viewport(layout.pos.row = 1, layout.pos.col = 2))
print(Fig5c, vp = viewport(layout.pos.row = 2, layout.pos.col = 1))
print(Fig5d, vp = viewport(layout.pos.row = 2, layout.pos.col = 2))

dev.off()
###End of Fig5####
####clear device
dev.off()


###Fig 6 - Survival Regression####


###Here come the Survregs
###Dont plot "Strain7" for space reasons
surv_strains <- c("Strain1","Strain2","Strain3","Strain5","Strain6","Strain4")

kmdata <- filter(surv_DI_fit.df, Strain %in% surv_strains)
curvdata <- filter(sreg_curves, Strain %in% surv_strains)
#Labels

ggwei <- ggplot(kmdata, aes(time, surv, colour = Strain)) +
  geom_step(aes(linetype=Batch)) +
  geom_line(data=curvdata,aes(y=wei.sreg),color="black") +
  facet_wrap(~Strain) + theme(legend.position="none")+
  ggtitle("c) Kaplan-Meier estimates and fit to Weibull distribution")+
  theme_bw()+
  labs(x = "Days post infection", y = "Fraction of plants alive") + 
  theme(legend.position="none")
###Plot of KM+Logistic
gglogis <- ggplot(kmdata, aes(time, surv, colour = Strain)) +
  geom_step(aes(linetype=Batch)) +
  geom_line(data=curvdata,aes(y=logis.sreg),color="black") +
  facet_wrap(~Strain) + theme(legend.position="none")+
  ggtitle("d) Kaplan-Meier estimates and fit to Logistic distribution")+
  theme_bw()+
  labs(x = "Days post infection", y = "Fraction of plants alive") + 
  theme(legend.position="none")
###Plot of KM+Gaussian  
gggaus <- ggplot(kmdata, aes(time, surv, colour = Strain)) +
  geom_step(aes(linetype=Batch)) +
  geom_line(data=curvdata,aes(y=gaus.sreg),color="black") +
  facet_wrap(~Strain) + theme(legend.position="none")+
  ggtitle("b) Kaplan-Meier estimates and fit to Gaussian distribution")+
  theme_bw()+
  labs(x = "Days post infection", y = "Fraction of plants alive") + 
  theme(legend.position="none")
###Plot of KM+Lognormal  
gglnorm <- ggplot(kmdata, aes(time, surv, colour = Strain)) +
  geom_step(aes(linetype=Batch)) +
  geom_line(data=curvdata,aes(y=lnorm.sreg),color="black") +
  facet_wrap(~Strain) + theme(legend.position="none")+
  ggtitle("a) Kaplan-Meier estimates and fit to Lognormal distribution")+
  theme_bw()+
  labs(x = "Days post infection", y = "Fraction of plants alive") + 
  theme(legend.position="none")

pushViewport(viewport(layout= grid.layout(nrow=2, ncol=2)))
print(gglnorm, vp = viewport(layout.pos.row = 1, layout.pos.col = 1))
print(gggaus, vp = viewport(layout.pos.row = 1, layout.pos.col = 2))
print(ggwei, vp = viewport(layout.pos.row = 2, layout.pos.col = 1))
print(gglogis, vp = viewport(layout.pos.row = 2, layout.pos.col = 2))
###End of Figure6###
##Clear device.
dev.off()

####MOdified Figure 6
lnorm.di <- cbind(as.character(sregparams$Strain[1:7]), 
                  round(as.numeric(sregparams$scale.lnorm[1:7]), digits = 4), 
                  round(as.numeric(sregparams$shape.lnorm[1:7]),  digits = 4),
                  round( as.numeric(exp(sregparams$shape.lnorm[1:7])), digits = 4))
colnames(lnorm.di) <- c("Strain","Scale","Shape","exp(Shape)")

gglnorm <- ggplot(kmdata, aes(time, surv)) +
  geom_step(aes(linetype=Batch)) +
  geom_line(data=curvdata,aes(y=lnorm.sreg),color="black") +
  facet_wrap(~Strain) + theme(legend.position="none")+
  ggtitle("Kaplan-Meier estimates and fit to Lognormal distribution")+
  theme_bw()+
  labs(x = "Days post infection", y = "Fraction of plants alive") + 
  theme(legend.position="none")

library(gridExtra)

# Set theme to allow for plotmath expressions
tt <- ttheme_default(colhead=list(fg_params = list(parse=TRUE)))
tbl <- tableGrob(lnorm.di, rows=NULL, theme=tt)
# Plot chart and table into one object
grid.arrange(tbl, gglnorm, 
             ncol=2,
             as.table=TRUE,
             heights=c(3,1))
```
