# Supplementary material for: A Practical Guide to Visualization and Statistical Analysis of R. solanacearum Infection Data Using R
Source: Front Plant Sci. 2017 Apr 24;8:623. doi: 10.3389/fpls.2017.00623 (PMC5401893; doi:10.3389/fpls.2017.00623)
Supplement: Supplementary file 2 [file Data_Sheet_2.ZIP › S2_Wang_dataset.html]

Approaches to analyze experimental R. solanacearum infections: Supplementary Material 2: Analysis of the Wang 2015 dataset


# Approaches to analyze experimental *R. solanacearum* infections: *Supplementary Material 2:* Analysis of the Wang 2015 dataset

#### *Niklas Schandry*

# General information on this document

This document is part of the supplementary material of “A practical guide to descriptive and statistical analysis of R. solanacearum infection data using R”. This document is identical to S1. Except the dataset that is used is that published by Wang, et al., 2015.

# Data Import, Formatting and preparation

## Specifying the data

Initially, data has to be read into R. Beforunte data can be read, the directory containing the data needs to be specified and set. In R this is called the working directory. Anything that is read into, or exported from R will use this directory unless pointed to a different directory.

```
###Install all required packages:
#install.packages(c("MESS","lme4","lmerTest","multcomp","survival","rms","coxme","stargazer","survcomp","tidyverse","rcompanion"))


###Define Working Directory and set it
###Note for the Markdown version: R-Markdown cannot set the working directory
###R markdown will always use the directory the .Rmd file is located in
###In the .Rmd file this code section is not actually evaluated and only serves illustratory purpose.
wd <- c("~/My_Data/DataDirectory/")
setwd(wd)
```

Next, the dataset is specified .

```
###Name of the file to be read
table <- c("S2_wang.csv")
```

## Reading data and formatting

The data can be read into *R* and stored in a data frame. I am calling that data frame disease\_index

```
disease_index <- as.data.frame(read.table(table, header=T,
                                          sep=";" , ###Sets the seperator of the csv file
                                          dec=","), ###Sets the decimal operator of the csv file
                                          stringsAsFactors=F) ###
```

Using str() one can see if the table was properly imported.

```
str(disease_index)
```

```
## 'data.frame':    476 obs. of  12 variables:
##  $ Strain: Factor w/ 7 levels "GMI1000","GRS138",..: 1 1 1 1 1 1 1 1 1 1 ...
##  $ Plant : Factor w/ 1 level "Medicago": 1 1 1 1 1 1 1 1 1 1 ...
##  $ X3    : Factor w/ 5 levels "0","0.00","1",..: 3 4 1 3 1 1 1 3 4 3 ...
##  $ X4    : Factor w/ 7 levels "0","0.00","1",..: 6 6 1 6 4 1 3 6 6 3 ...
##  $ X5    : Factor w/ 7 levels "0","0.00","1",..: 6 6 3 6 4 1 6 6 5 4 ...
##  $ X6    : Factor w/ 6 levels "0","1","2","3",..: 5 5 4 5 5 1 5 5 5 4 ...
##  $ X7    : Factor w/ 7 levels "0","1","2","2.5",..: 7 7 6 7 7 4 7 7 7 6 ...
##  $ X8    : logi  NA NA NA NA NA NA ...
##  $ X9    : logi  NA NA NA NA NA NA ...
##  $ X10   : Factor w/ 6 levels "0","1","2","3",..: 6 6 6 6 6 6 6 6 6 6 ...
##  $ X11   : Factor w/ 8 levels "0","0.00","1",..: NA NA NA NA NA NA NA NA NA NA ...
##  $ Batch : Factor w/ 5 levels "A","B-I","B-II",..: 1 1 1 1 1 1 1 1 1 1 ...
```

If the table was properly imported, “Xnumber” columns should be either numeric, or logical (if they were empty). If this is the case, generation of a “subject”" column helps further analysis, which assings a unique, numeric identifier to each individual.

```
disease_index$subject <- c(1:nrow(disease_index))
```

Now, the table of disease index recordings, needs to converted into long format for later analysis, conforming with a data structure often referred to as “tidy” (Wickham, 2008) . Long format means, that instead of having one column of each day, a new column is generated that indicates the day post infection (DPI).

```
library("tidyr")
```

```
di_long_na <- gather(disease_index, key=DPI, 
                     value=DI,
                     X3:X11, ####Specify here the days the plants were observed (this means 3 to 11)
                     na.rm=F)
```

```
## Warning: attributes are not identical across measure variables; they will
## be dropped
```

Which will generate a long table, containing missing observations coded as NA. The table structure is as follows:

```
str(di_long_na)
```

```
## 'data.frame':    4284 obs. of  6 variables:
##  $ Strain : Factor w/ 7 levels "GMI1000","GRS138",..: 1 1 1 1 1 1 1 1 1 1 ...
##  $ Plant  : Factor w/ 1 level "Medicago": 1 1 1 1 1 1 1 1 1 1 ...
##  $ Batch  : Factor w/ 5 levels "A","B-I","B-II",..: 1 1 1 1 1 1 1 1 1 1 ...
##  $ subject: int  1 2 3 4 5 6 7 8 9 10 ...
##  $ DPI    : chr  "X3" "X3" "X3" "X3" ...
##  $ DI     : chr  "1" "2" "0" "1" ...
```

Next, some modifications the table structure are neccessary to make sure that all variables (columns) are in the proper format.

```
di_long_na$DI <- as.numeric(di_long_na$DI) ###Turns DI into numeric
di_long <- na.omit(di_long_na) ###Generate a table that does not contain missing observations
di_long$DPI <- na.omit( as.numeric (unlist( strsplit( as.character( di_long$DPI ), "X" ) ) ) )  ###Discard the "X" and save the number as a number (instead of a factor), it is not elegant, but it works
```

## Defining contrasts

A crucial step that will influence the statistical analysis is setting the “contrasts”. Contrasts specify the reference for each of the variables. “Treatment” contrasts specify that the first alphabetical level will be used as a reference for all others (see Strain below), while a “sum” constrast means that the reference value is the mean across all levels of that variable (the grand mean).

```
####Specify what should be "appropriate" contrasts
contrasts(di_long$Strain) <- "contr.treatment"###First alphabetical strain will be the baseline!
#contrasts(di_long$Plant) <- "contr.Treatment"###First alphabetical plant will be the baseline!
contrasts(di_long$Batch) <- "contr.sum" ###Batches will be averaged to generate the baseline!
#contrasts(di_long$DI) <- "contr.poly" ###This is currently not useful because Di is only used as response variable
```

## Add a censoring variable

Now that the data has been read into R, and formatted properly, a new variable is added to the data. This variable is called “Useful” and is a binary yes/no variable. The purpose of this variable is marking observations, that are re-observations of a subject that has previously reached disease index 4. As disease index of 4 means 100% wilting, the plant died when it reached this degree of wilting. Since death is permanent, continuing to observe this plant is unlikely to provide new information.

```
###Interesting R-related observation, the below does not work when subsetting is done with filter(), 
###because filter does not retain rownames!
for (i in 1:max(di_long$subject)) { ###Go by subject
  dummy1 <- di_long[di_long$subject==i,] ##Create a first dummy object, that is a subset of the full data containing the current subject 
  if(min(dummy1$DI) == 0){
  #remove those observations that are before disease onset, except the one directly before disease onset.
  dummy1 <- dummy1[dummy1$DPI %in% (max(dummy1$DPI[dummy1$DI==0]):max(dummy1$DPI)),]
  } 
  if (max(dummy1$DI) == 4) { ###If this subject dies at some point
    dummy2 <- dummy1[dummy1$DI==4,] ###Make a new dummy object, that only contains those recordings where DI=4
    NEW <- di_long[di_long$subject==i & (di_long$DPI %in% min(dummy1$DPI):min(dummy2$DPI)),] ###Generate data subset "NEW", which contains those observations for a subject, that are between (including) the last recording where DI=0 and the first recording where disease index is 4.
  } else { ###If this dubject does not die
    NEW <- dummy1 ###New is the same as dummy1
  }
  di_long$Useful[rownames(di_long) %in% rownames(NEW)] <- c("Yes") ###All of those row(names) that are part of the "NEW" object are useful. Therefore these receive status "Yes" in column "Useful"
}
di_long$Useful[which(is.na(di_long$Useful))] <- c("No") ###Those that are not yes (and therefore are NA) become No
rm(dummy1,dummy2)
```

After this and the earlier code blocks have been run, the di\_long data frame should look similar to this:

```
library("broom")
```

```
str(di_long)
```

```
## 'data.frame':    2856 obs. of  7 variables:
##  $ Strain : Factor w/ 7 levels "GMI1000","GRS138",..: 1 1 1 1 1 1 1 1 1 1 ...
##   ..- attr(*, "contrasts")= chr "contr.treatment"
##  $ Plant  : Factor w/ 1 level "Medicago": 1 1 1 1 1 1 1 1 1 1 ...
##  $ Batch  : Factor w/ 5 levels "A","B-I","B-II",..: 1 1 1 1 1 1 1 1 1 1 ...
##   ..- attr(*, "contrasts")= chr "contr.sum"
##  $ subject: int  1 2 3 4 5 6 7 8 9 10 ...
##  $ DPI    : atomic  3 3 3 3 3 3 3 3 3 3 ...
##   ..- attr(*, "na.action")=Class 'omit'  int [1:2856] 1 3 5 7 9 11 13 15 17 19 ...
##  $ DI     : num  1 2 0 1 0 0 0 1 2 1 ...
##  $ Useful : chr  "Yes" "Yes" "No" "Yes" ...
##  - attr(*, "na.action")=Class 'omit'  Named int [1:1428] 1541 1542 1543 1544 1545 1546 1547 1548 1549 1550 ...
##   .. ..- attr(*, "names")= chr [1:1428] "1541" "1542" "1543" "1544" ...
```

```
tidy(di_long) ###This can be used to assess the descriptive statistics of the data. It may be adivsable to look at SKEW and KURTOSIS of the DI (response) to determined wether this is approximately normally distributed (both close to 0, if they are above "absolute 1" the data is probably really not normal).
```

```
## Warning: NAs introduced by coercion
```

```
## Warning in FUN(newX[, i], ...): no non-missing arguments to min; returning
## Inf
```

```
## Warning in FUN(newX[, i], ...): no non-missing arguments to max; returning
## -Inf
```

```
##    column    n       mean         sd median    trimmed      mad min  max
## 1 Strain* 2856   4.000000   2.000350    4.0   4.000000   2.9652   1    7
## 2  Plant* 2856   1.000000   0.000000    1.0   1.000000   0.0000   1    1
## 3  Batch* 2856   2.911765   1.442629    3.0   2.889764   1.4826   1    5
## 4 subject 2856 238.500000 137.433123  238.5 238.500000 176.4294   1  476
## 5     DPI 2856   6.470588   2.852076    5.5   6.338583   2.2239   3   11
## 6      DI 2856   1.480042   1.790993    0.0   1.350394   0.0000   0    4
## 7 Useful* 2856        NaN         NA     NA        NaN       NA Inf -Inf
##   range       skew  kurtosis         se
## 1     6 0.00000000 -1.251225 0.03743061
## 2     0        NaN       NaN 0.00000000
## 3     4 0.06609884 -1.342605 0.02699452
## 4   475 0.00000000 -1.201271 2.57165269
## 5     8 0.39572616 -1.327143 0.05336813
## 6     4 0.50867789 -1.611508 0.03351312
## 7  -Inf         NA        NA         NA
```

With this, the data has been imported into R, and formatted according to the needs of *most* of the subsequent analysis. When actively trying out different analysis, it may be advisable to generate a backup copy of the table.

```
di_long_full <- di_long
```

Alternatively, this table can also be exported.

```
write.csv(di_long, file = "My_Formatted_Data.csv")
```

# Analysis of the AUDPC

## Rationale and data preparation

Area under the disease progression curve (AUDPC) is a classical approach to analyze data of disease progression recordings. Here, the area under the curve is calculated, and the curve is drawn using Disease index (y) and time (x). To get an overall impression per experiment, one can calculate the average disease index per day, within one experimental group, using:

```
library("dplyr")
```

```
di_summary <- di_long %>% group_by(Plant, Strain, Batch, DPI) %>% 
  summarise(mean(DI),sd(DI),sd(DI)/sqrt(length(DI))) ##calculate within Batch mean, sd, and se for each Plant/Strain combination.
##Averages within each replicate. This summary table is mainly helpful for plotting, not used for analysis...
colnames(di_summary) <- c("Plant", "Strain", "Batch", "DPI", "mean", "sd", "se") ###Assign correct columnnames
```

Using these summaries, one can take a look at the averaged disease progression.

```
library("ggplot2")
```

```
ggplot(filter(di_summary, Batch=="A")) + ###Use Only Batch A (too busy otherwise)
  aes(x=DPI,y=mean,color=Strain) + ###Color by Strain, specify x and y.
  geom_area(aes(fill=Strain),position="identity",alpha=0.15) + ###Area plot, colored by Strain
  ##geom_errorbar(aes(x=DPI, ymax=mean+se, ymin=mean-se), size=0.25)+ ###This line adds SE. 
  ##geom_errorbar(aes(x=DPI, ymax=mean+sd, ymin=mean-sd), size=0.25)+ ###This line adds SD. Don't use both.
  facet_wrap(~Strain) + ###One plot per strain 
  labs(x = "Days post infection", y = "Avg. Disease Index") + #Labels
  ggtitle("Figure 1\nDisease Areas,\nper strain, for batch A") #Title
```

From this plot, one can see that in the example dataset the areas differ quite drastically between different strains. As can be seen here, all observations can be included in an AUDCP analysis, but one should take care that total observation times are similar, identical if possible. As the area increases with both, increased disease index and prolonged time, experiments of different length should not be compared using this approach. To calculated the actual AUDPC for each individual in the dataset a new data frame is created. As AUDCP is calculated from both disease index and DPI, this can not be stored in a reasonable way in the long data frame generated earlier.

```
library("MESS")
```

```
####Build a table of AUDCPs, per subject
auc_df <- data.frame() ###Make auc_df data frame
for (i in 1:max(di_long$subject)) { ##Go by subject
  temp <- di_long[di_long$subject==i,] ###Subset full table into the subject table
  temp <- droplevels(temp) ###Drop levels, so levels works properly below
  auc_df[i,1] <- i ###Subject number
  auc_df[i,2] <- levels(temp$Strain) ###Strain
  auc_df[i,3] <- levels(temp$Plant) ###Plant
  auc_df[i,4] <- levels(temp$Batch) ###Batch
  auc_df[i,5] <- auc(temp$DPI,temp$DI) ###AUC; i assume that trapezoid rule is fine here. 
  ### Additionally, auc calculation starts with the lowest x (DPI). I think this is sensible
  ### I assume that if one specifies "from=0", the curve is expanded by a triangle that covers the range from 
  ### 0 to whatever is the value at the first observation. I think the first observation should ideally be 0
  ### if data was recorded from the beginning..
}
colnames(auc_df) <- c("subject","Strain","Plant","Batch","AUC") ###Name columns in AUC datafarame
auc_df$Strain <- as.factor(auc_df$Strain) #refactor
auc_df$Plant <- as.factor(auc_df$Plant) #refactor
auc_df$Batch <- as.factor(auc_df$Batch) #refactor
str(auc_df)
```

```
## 'data.frame':    476 obs. of  5 variables:
##  $ subject: int  1 2 3 4 5 6 7 8 9 10 ...
##  $ Strain : Factor w/ 7 levels "GMI1000","GRS138",..: 1 1 1 1 1 1 1 1 1 1 ...
##  $ Plant  : Factor w/ 1 level "Medicago": 1 1 1 1 1 1 1 1 1 1 ...
##  $ Batch  : Factor w/ 5 levels "A","B-I","B-II",..: 1 1 1 1 1 1 1 1 1 1 ...
##  $ AUC    : num  24.5 25 17 24.5 21.5 11 22 24.5 24.5 19.5 ...
```

The auc data.frame contains one area under the curve per subject and all other subject specific variables as stored in the original table.

## Analysis of differences in area under the disease progression curve

An initial assessment of strain specific differences in AUDPC can be performed visually, for example by generating boxpots.

```
ggplot(auc_df) + geom_boxplot(
  aes(x=Strain, y=AUC, color=Strain),  #Plot boxplots of AUCs, by strains
  notch=F) +
  labs(y="AUDPC") +
  facet_wrap(~Batch) + ###Individual plots per batch (and plant if applicable)
  ggtitle("Figure 2\nArea Under the Disease Progression Curve per strain across batches")
```

Next, one can use the area under the disease progression curve, to build a linear model, or a linear mixed effects model.

```
library("lme4")
library("lmerTest")
```

```
summary(lm(AUC~Batch,data=auc_df)) ###Can be used to identify batch effects. If there are none, including batch as a random factor below is not necessary (but also not necessarily wrong).
```

```
## 
## Call:
## lm(formula = AUC ~ Batch, data = auc_df)
## 
## Residuals:
##     Min      1Q  Median      3Q     Max 
## -16.632 -12.821   4.978   9.591  15.429 
## 
## Coefficients:
##             Estimate Std. Error t value Pr(>|t|)    
## (Intercept) 12.91741    1.01962  12.669   <2e-16 ***
## BatchB-I    -0.09598    1.52288  -0.063   0.9498    
## BatchB-II    3.71446    1.52288   2.439   0.0151 *  
## BatchC-I     0.56611    1.52288   0.372   0.7103    
## BatchC-II   -0.39543    1.52288  -0.260   0.7952    
## ---
## Signif. codes:  0 '***' 0.001 '**' 0.01 '*' 0.05 '.' 0.1 ' ' 1
## 
## Residual standard error: 10.79 on 471 degrees of freedom
## Multiple R-squared:  0.01879,    Adjusted R-squared:  0.01045 
## F-statistic: 2.254 on 4 and 471 DF,  p-value: 0.06233
```

```
auc_lmer <- lmer(
  AUC ~ Strain + (1|Batch),   ### AUC modeled as a function of strain, random effects of batch 
    data=auc_df  ) #Linear mixed effects model.

auc_lm <- lm(AUC~Strain+Batch,data=auc_df) #Linear model.

AIC(auc_lm,auc_lmer) #Lower AIC, better fit, linear model is slightly better.
```

```
##          df      AIC
## auc_lm   12 2837.014
## auc_lmer  9 2840.157
```

```
ggplot(data=auc_df, aes(y=AUC, x=Strain)) +geom_boxplot(aes(colour=Batch)) + ggtitle("Boxplot of AUDCP per strain by experimental batch")
```

The model can be explored using various functions, such as summary.

```
summary(auc_lmer)  ### A model summary, containing information on the model.
```

```
## Linear mixed model fit by REML t-tests use Satterthwaite approximations
##   to degrees of freedom [lmerMod]
## Formula: AUC ~ Strain + (1 | Batch)
##    Data: auc_df
## 
## REML criterion at convergence: 2822.2
## 
## Scaled residuals: 
##     Min      1Q  Median      3Q     Max 
## -4.5888 -0.3924  0.0755  0.4577  3.0258 
## 
## Random effects:
##  Groups   Name        Variance Std.Dev.
##  Batch    (Intercept)  2.606   1.614   
##  Residual             22.092   4.700   
## Number of obs: 476, groups:  Batch, 5
## 
## Fixed effects:
##              Estimate Std. Error       df t value Pr(>|t|)    
## (Intercept)   23.5530     0.9199   8.8000  25.603 1.38e-09 ***
## StrainGRS138 -15.9816     0.8061 465.0000 -19.826  < 2e-16 ***
## StrainGRS447 -23.2059     0.8061 465.0000 -28.789  < 2e-16 ***
## StrainGRS460 -22.9412     0.8061 465.0000 -28.460  < 2e-16 ***
## StrainRs517   -3.1581     0.8061 465.0000  -3.918 0.000103 ***
## StrainRs531   -1.2059     0.8061 465.0000  -1.496 0.135337    
## StrainRs549   -2.6691     0.8061 465.0000  -3.311 0.001001 ** 
## ---
## Signif. codes:  0 '***' 0.001 '**' 0.01 '*' 0.05 '.' 0.1 ' ' 1
## 
## Correlation of Fixed Effects:
##             (Intr) SGRS13 SGRS44 SGRS46 StR517 StR531
## StranGRS138 -0.438                                   
## StranGRS447 -0.438  0.500                            
## StranGRS460 -0.438  0.500  0.500                     
## StrainRs517 -0.438  0.500  0.500  0.500              
## StrainRs531 -0.438  0.500  0.500  0.500  0.500       
## StrainRs549 -0.438  0.500  0.500  0.500  0.500  0.500
```

```
tidy(auc_lmer) ### A cleaner display using tidy.
```

```
##                      term   estimate std.error  statistic    group
## 1             (Intercept)  23.553039 0.9199479  25.602579    fixed
## 2            StrainGRS138 -15.981618 0.8060808 -19.826322    fixed
## 3            StrainGRS447 -23.205882 0.8060808 -28.788531    fixed
## 4            StrainGRS460 -22.941176 0.8060808 -28.460145    fixed
## 5             StrainRs517  -3.158088 0.8060808  -3.917831    fixed
## 6             StrainRs531  -1.205882 0.8060808  -1.495982    fixed
## 7             StrainRs549  -2.669118 0.8060808  -3.311228    fixed
## 8    sd_(Intercept).Batch   1.614192        NA         NA    Batch
## 9 sd_Observation.Residual   4.700218        NA         NA Residual
```

```
###The tidy output explained:
#Term: A discription: (Intercept) overall intercept. Intercept depends on the contrasts set initially. Here treatment contrasts are used, so Intercept = First alphabetical strain (Strain1).
#StrainStrain2: Difference in the estimate (slope), between Strain2 and the (Intercept)

##Estimate: The estimated slope
```

For example, we see the estimated slopes (Estimate) and standard errors, together with a t- and corresponding p-value in the output of the summary function. Note, that the above only contains information on differences between different levels and the “baseline”“, which is called (Intercept). The baseline is determined by the contrast settings that were specified earlier.

But, it may be quite relevant to know how individual strains compare to each other. This can be analyzed using a generalized linear hypothesis test, while ajusting for multiple comparisons using Tukey’s method.

```
library("multcomp")
library("rcompanion")
library("stringr")
```

```
tidy(summary(glht(auc_lmer, linfct=mcp(Strain="Tukey"))))
```

```
##                 lhs rhs    estimate std.error   statistic      p.value
## 1  GRS138 - GMI1000   0 -15.9816176 0.8060808 -19.8263221 0.000000e+00
## 2  GRS447 - GMI1000   0 -23.2058824 0.8060808 -28.7885312 0.000000e+00
## 3  GRS460 - GMI1000   0 -22.9411765 0.8060808 -28.4601449 0.000000e+00
## 4   Rs517 - GMI1000   0  -3.1580882 0.8060808  -3.9178308 1.720359e-03
## 5   Rs531 - GMI1000   0  -1.2058824 0.8060808  -1.4959820 7.474866e-01
## 6   Rs549 - GMI1000   0  -2.6691176 0.8060808  -3.3112284 1.633421e-02
## 7   GRS447 - GRS138   0  -7.2242647 0.8060808  -8.9622091 0.000000e+00
## 8   GRS460 - GRS138   0  -6.9595588 0.8060808  -8.6338228 1.110223e-16
## 9    Rs517 - GRS138   0  12.8235294 0.8060808  15.9084913 0.000000e+00
## 10   Rs531 - GRS138   0  14.7757353 0.8060808  18.3303401 0.000000e+00
## 11   Rs549 - GRS138   0  13.3125000 0.8060808  16.5150937 0.000000e+00
## 12  GRS460 - GRS447   0   0.2647059 0.8060808   0.3283863 9.999001e-01
## 13   Rs517 - GRS447   0  20.0477941 0.8060808  24.8707004 0.000000e+00
## 14   Rs531 - GRS447   0  22.0000000 0.8060808  27.2925492 0.000000e+00
## 15   Rs549 - GRS447   0  20.5367647 0.8060808  25.4773028 0.000000e+00
## 16   Rs517 - GRS460   0  19.7830882 0.8060808  24.5423141 0.000000e+00
## 17   Rs531 - GRS460   0  21.7352941 0.8060808  26.9641629 0.000000e+00
## 18   Rs549 - GRS460   0  20.2720588 0.8060808  25.1489165 0.000000e+00
## 19    Rs531 - Rs517   0   1.9522059 0.8060808   2.4218489 1.890481e-01
## 20    Rs549 - Rs517   0   0.4889706 0.8060808   0.6066024 9.966325e-01
## 21    Rs549 - Rs531   0  -1.4632353 0.8060808  -1.8152464 5.374807e-01
```

This information can, for example, be integrated into a boxplot of the individual disease areas. For example, using a compact letter display, in conbination with AUDPC, combines statistical and visual information. First, however, the grouping letters need to be calculated. Then, these letters are added to the boxplot generated earlier.

```
auc_cld <- cld(glht(auc_lmer, linfct=mcp(Strain="Tukey"))) ###Save letters
```

```
## Warning in RET$pfunction("adjusted", ...): Completion with error > abseps
```

```
auc_cld <- cbind(levels(auc_df$Strain),auc_cld$mcletters$Letters) ###bind letters to columns
colnames(auc_cld) <- c("Strain","Letter") ###Name columns
auc_cld <- as.data.frame(auc_cld) #Coerce to dataframe
###Integrate letters into auc_df##
auc_df <- left_join(auc_df,auc_cld,by="Strain",copy=T) ###Add letter information
###Some extra scripting to make the mean and CI plot.
auc_CI <- as.data.frame(tidy(confint(auc_lmer)))
```

```
## Computing profile confidence intervals ...
```

```
## Warning in optwrap(optimizer, par = thopt, fn = mkdevfun(rho, 0L), lower
## = fitted@lower): convergence code 3 from bobyqa: bobyqa -- a trust region
## step failed to reduce q
```

```
auc_CI <- auc_CI[3:nrow(auc_CI),] ##Drop sig01, sigma
auc_CI$Strain <- levels(auc_df$Strain)
###Mean relative to Strain1 (except strain1 that one is absolute)

for (i in 1:nrow(auc_CI)){
  if (i==1){
    auc_CI$mean[i] <- mean(c(auc_CI$X2.5..[i],auc_CI$X97.5..[i]))
    auc_CI$upr[i] <- auc_CI$X97.5..[i]
    auc_CI$lwr[i] <- auc_CI$X2.5..[i]
  } else {
    auc_CI$mean[i] <- c(auc_CI$mean[1]+mean(c(auc_CI$X2.5..[i],auc_CI$X97.5..[i])))
    auc_CI$upr[i] <- c(auc_CI$mean[1]+auc_CI$X97.5..[i])
    auc_CI$lwr[i] <- c(auc_CI$mean[1]+auc_CI$X2.5..[i])
  }
}
 
####Generate plot of meanCI of AUDCP with significance letters and raw data as jittered points
  ggplot(aes(x=Strain, y=AUC, color=Strain),data=auc_df) + ###Plot the auc_df
  geom_crossbar(data = auc_CI, aes(x = Strain, y = mean, ymin = lwr, ymax = upr,fill=Strain), alpha=0.3) +
  geom_jitter(aes(shape=Batch)) + ###with jitter overplotted, symbol shape defined by batch
  geom_text(aes(x=Strain, y=-3, label=Letter),color="black", data=auc_cld) + ###Get the letters from auc_cld 
  #and write those to position y=-3
  labs(y="AUDPC") + #Y-Axis label
  ggtitle("AUDPC raw values and mean from the LMM\nwith 95% CI per strain and grouping letters") #Title
```

Finally, one can plot the pairwise difference in AUDPC means with confidence intervals for the linear model and the linear mixed effects model.

```
pairwise_confint_AUDPC_lm <- as.data.frame(confint(glht(auc_lm, mcp(Strain = "Tukey")))$confint)
pairwise_confint_AUDPC_lm$Comparison <- rownames(pairwise_confint_AUDPC_lm)

pairwise_confint_AUDPC_lmer <- as.data.frame(confint(glht(auc_lmer, mcp(Strain = "Tukey")))$confint)
pairwise_confint_AUDPC_lmer$Comparison <- rownames(pairwise_confint_AUDPC_lmer)

###Plot the comparisons, below may not be the most straight-foward way to plot this the way I want it, but it works.
ggplot(pairwise_confint_AUDPC_lm, aes(x = Comparison, y = Estimate, ymin = lwr, ymax = upr, color = abs(Estimate))) + ###Plot Comparison on x, estimate on y
  scale_x_discrete(limits = rev(levels(as.factor(pairwise_confint_AUDPC_lm$Comparison)))) + ###Rescale x, so the order is inverted 
  geom_errorbar() + geom_point() + ###Draw data
  coord_flip() +  theme(legend.position="none") + xlab("") +###Invert X and Y, hide legend
  ggtitle("Difference in means of the AUDPC \nin the linear model with 95% confidence interval") ##Add a title
```

```
####Plot of the comparisons in the LMM. Here the bars are colored by their absolute value, instead of by their name.
ggplot(pairwise_confint_AUDPC_lmer, aes(x = Comparison, y = Estimate, ymin = lwr, ymax = upr, color = abs(Estimate))) + ###Plot Comparison on x, estimate on y
  scale_x_discrete(limits = rev(levels(as.factor(pairwise_confint_AUDPC_lmer$Comparison)))) + ###Rescale x, so the order is inverted 
  geom_errorbar() + geom_point() + ###Draw data
  coord_flip() +  theme(legend.position="none") + xlab("") +###Invert X and Y, hide legend
  ggtitle("Difference in means of the AUDPC \nin the LMM with 95% confidence interval") ##Add a title
```

Estimates of lm and lmm are very similar. As a rule of thumb, comparisons where “0” is not part of the 95% confidence interval are likely to produce a signficant p-value (assuming significance is denote by p<0.05).

# Analysis of Disease development

## Using a Repeated Measure ANOVA.

Repeated-measure ANOVA can be used to analyze DI and time. However, when using repeated measure ANOVA one should be aware that the arrow of time is not considered in this analysis. Here the variable denoting the measurements is put into the Error() term.

```
#Linear model of rep measure is as follows
rm_aov <- aov(DI~Strain + Error(DPI), data = di_long)
###The below Provides differences to Strain1. 
summary(rm_aov, split=list(Strain = list(Strain2=1,Strain3=2,Strain4=3,Strain5=4,Strain6=5,Strain7=6)))
```

```
## 
## Error: DPI
##           Df Sum Sq Mean Sq F value Pr(>F)
## Residuals  1   2491    2491               
## 
## Error: Within
##                     Df Sum Sq Mean Sq  F value   Pr(>F)    
## Strain               6   3083   513.8  408.207  < 2e-16 ***
##   Strain: Strain2    1    186   185.7  147.572  < 2e-16 ***
##   Strain: Strain3    1   1169  1168.6  928.510  < 2e-16 ***
##   Strain: Strain4    1   1693  1693.1 1345.302  < 2e-16 ***
##   Strain: Strain5    1     16    16.1   12.814 0.000350 ***
##   Strain: Strain6    1      1     0.5    0.432 0.510860    
##   Strain: Strain7    1     18    18.4   14.612 0.000135 ***
## Residuals         2848   3584     1.3                      
## ---
## Signif. codes:  0 '***' 0.001 '**' 0.01 '*' 0.05 '.' 0.1 ' ' 1
```

```
###I personally think that LMMs are nicer to investigate and offer greater flexibility.
```

## Linear mixed effects model.

### Rationale

In this section, the data is analyzed using a linear mixed effect model. While such models have been used already in the previous section, to test for a strain specific influence on the area under the disease progression curve, different data is used to build the model in this section. As mentioned, AUDPC summarizes disease incidence and time into a single variable, the area. However, in certain cases the AUDCP could be very similar, while the actual disease progression is different. Taking a look at Figure 1, we see that Strain2 and Strain3 display different disease progressions. For Strain 2, the Disease Index continually rises over time. For Strain3, the the disease appears to set on very quickly, in fact before the observations started, but from there it does not increase in severity. However, if we compare the impact of those two strains on the AUDCP (Fig2, 3), it becomes evident, that the AUDPCs for these strains are not statistically different, both strains belong to group “a”. Other methods may be more sensitive to such differences. Below a different approach using linear mixed effect models is taken.

### Model

A linear mixed effects model is generated. Here, the Disease index is modeled on the fixed effects “Day post infection” and “Strain”. Batch and subject are included as random effects, meaning they is not of direct interest, but assumed to introduce variation, specifically by affecting the intercept.

```
###Define contrasts for lmer
contrasts(di_long$Strain) <- "contr.treatment"###First alphabetical strain will be the baseline!
#contrasts(di_long$Plant) <- "contr.treatment"###First alphabetical plant will be the baseline!
contrasts(di_long$Batch) <- "contr.poly" ###Batches will be averaged
###Drop things that are not "Useful"
di_long_useful <- filter(di_long, Useful=="Yes")
str(di_long_useful)
```

```
## 'data.frame':    1248 obs. of  7 variables:
##  $ Strain : Factor w/ 7 levels "GMI1000","GRS138",..: 1 1 1 1 1 1 1 1 1 1 ...
##   ..- attr(*, "contrasts")= chr "contr.treatment"
##  $ Plant  : Factor w/ 1 level "Medicago": 1 1 1 1 1 1 1 1 1 1 ...
##  $ Batch  : Factor w/ 5 levels "A","B-I","B-II",..: 1 1 1 1 1 1 1 1 1 1 ...
##   ..- attr(*, "contrasts")= chr "contr.poly"
##  $ subject: int  1 2 4 5 7 8 9 10 11 12 ...
##  $ DPI    : atomic  3 3 3 3 3 3 3 3 3 3 ...
##   ..- attr(*, "na.action")=Class 'omit'  int [1:2856] 1 3 5 7 9 11 13 15 17 19 ...
##  $ DI     : num  1 2 1 0 0 1 2 1 0 2 ...
##  $ Useful : chr  "Yes" "Yes" "Yes" "Yes" ...
##  - attr(*, "na.action")=Class 'omit'  Named int [1:1428] 1541 1542 1543 1544 1545 1546 1547 1548 1549 1550 ...
##   .. ..- attr(*, "names")= chr [1:1428] "1541" "1542" "1543" "1544" ...
```

```
## Build linear mixed effect model(s) ####
disease_lmer <- lmer(DI ~ Strain + Strain:DPI + (1 | subject) + (1 | Batch), di_long_useful)
```

The model can be investigated using summary functions. The pairwise comparisons may be plotted with 95%CI to assess how different two strains are.

```
###Check model summary
summary(disease_lmer)
```

```
## Linear mixed model fit by REML t-tests use Satterthwaite approximations
##   to degrees of freedom [lmerMod]
## Formula: DI ~ Strain + Strain:DPI + (1 | subject) + (1 | Batch)
##    Data: di_long_useful
## 
## REML criterion at convergence: 3846.9
## 
## Scaled residuals: 
##     Min      1Q  Median      3Q     Max 
## -4.3707 -0.5894  0.0215  0.6755  2.5070 
## 
## Random effects:
##  Groups   Name        Variance Std.Dev.
##  subject  (Intercept) 0.7443   0.8627  
##  Batch    (Intercept) 0.1899   0.4357  
##  Residual             0.7897   0.8887  
## Number of obs: 1248, groups:  subject, 476; Batch, 5
## 
## Fixed effects:
##                     Estimate Std. Error         df t value Pr(>|t|)    
## (Intercept)         -2.04347    0.29924   19.60000  -6.829 1.39e-06 ***
## StrainGRS138        -0.46227    0.38765 1228.90000  -1.193 0.233295    
## StrainGRS447         0.22651    0.97943 1088.30000   0.231 0.817153    
## StrainGRS460        -0.89483    0.91072 1211.40000  -0.983 0.326025    
## StrainRs517         -0.90661    0.34267 1227.60000  -2.646 0.008255 ** 
## StrainRs531         -1.32951    0.34614 1199.20000  -3.841 0.000129 ***
## StrainRs549         -0.49194    0.34049 1223.50000  -1.445 0.148770    
## StrainGMI1000:DPI    0.82182    0.03645  593.60000  22.549  < 2e-16 ***
## StrainGRS138:DPI     0.44806    0.03356  977.50000  13.351  < 2e-16 ***
## StrainGRS447:DPI     0.18070    0.08933 1011.00000   2.023 0.043352 *  
## StrainGRS460:DPI     0.29456    0.08334 1165.20000   3.534 0.000425 ***
## StrainRs517:DPI      0.86857    0.03845  793.60000  22.590  < 2e-16 ***
## StrainRs531:DPI      1.02331    0.04310  620.40000  23.740  < 2e-16 ***
## StrainRs549:DPI      0.81817    0.03947  936.60000  20.727  < 2e-16 ***
## ---
## Signif. codes:  0 '***' 0.001 '**' 0.01 '*' 0.05 '.' 0.1 ' ' 1
```

```
## 
## Correlation matrix not shown by default, as p = 14 > 12.
## Use print(x, correlation=TRUE)  or
##   vcov(x)     if you need it
```

```
###E.g. plot confints
###Make pairwise confints and plot them, with a flipped coordinate system
pairwise_confint <- as.data.frame(confint(glht(disease_lmer, mcp(Strain = "Tukey", interaction_average=T)))$confint)
pairwise_confint$Comparison <- rownames(pairwise_confint)

ggplot(pairwise_confint, aes(x = Comparison, y = Estimate, ymin = lwr, ymax = upr, color = abs(Estimate))) + ###Plot Comparison on x, estimate on y
  scale_x_discrete(limits = rev(levels(as.factor(pairwise_confint$Comparison)))) + ###Rescale x, so the order is inverted 
  geom_errorbar() + geom_point() + ###Draw data
  coord_flip() +  theme(legend.position="none") + xlab("") +###Invert X and Y, hide legend
  ggtitle("Difference in means with 95% confidence interval \ncolored by absolute estimated difference") ##Add a title
```

```
confint_model <- as.data.frame(tidy(confint(disease_lmer)))
```

```
## Computing profile confidence intervals ...
```

```
confint_slopes <- confint_model[ (1 + 3 + nlevels( di_long$Strain ) ) : ( 3 + 2*nlevels( di_long$Strain ) ) , 2:3 ]

colnames(confint_slopes) <- c("lwr","upr")
confint_slopes$Estimate <- rowMeans(confint_slopes)
confint_slopes$Strain <- levels(di_long$Strain)

###Plot the estimates, below may not be the most straight-foward way to plot this the way I want it, but it works.
ggplot(confint_slopes, aes(x = Strain, y = Estimate, ymin = lwr, ymax = upr, color = abs(Estimate))) + ###Plot Comparison on x, estimate on y
  scale_x_discrete(limits = rev(levels(as.factor(confint_slopes$Strain)))) + ###Rescale x, so the order is inverted 
  geom_errorbar() + geom_point() + ###Draw data
  coord_flip() +  theme(legend.position="none") + xlab("") +###Invert X and Y, hide legend
  ggtitle("Absolute slopes with 95%CI") ##Add a title
```

```
#Intercepts are treatment contrasted
confint_icep <- confint_model[4:( 3 + nlevels( di_long$Strain )),]

confint_icep$Strain <- levels(di_long$Strain)

for (i in 1:nrow(confint_icep)){
  if (i==1){
    confint_icep$Estimate[i] <- mean(c(confint_icep$X2.5..[i],confint_icep$X97.5..[i]))
    confint_icep$upr[i] <- confint_icep$X97.5..[i]
    confint_icep$lwr[i] <- confint_icep$X2.5..[i]
  } else {
    confint_icep$Estimate[i] <- c(confint_icep$Estimate[1]+mean(c(confint_icep$X2.5..[i],confint_icep$X97.5..[i])))
    confint_icep$upr[i] <- c(confint_icep$Estimate[1]+confint_icep$X97.5..[i])
    confint_icep$lwr[i] <- c(confint_icep$Estimate[1]+confint_icep$X2.5..[i])
  }
}

ggplot(confint_icep, aes(x = Strain, y = Estimate, ymin = lwr, ymax = upr, color = abs(Estimate))) + ###Plot Comparison on x, estimate on y
  scale_x_discrete(limits = rev(levels(as.factor(confint_icep$Strain)))) + ###Rescale x, so the order is inverted 
  geom_errorbar() + geom_point() + ###Draw data
  coord_flip() +  theme(legend.position="none") + xlab("") +###Invert X and Y, hide legend
  ggtitle("Absolute intercept with 95%CI") ##Add a title
```

Essentially, all of the tools used to analyze the linear model for area under the disease progression curve, can be applied to the disease model:

```
###Test hypothesis that all strains are equal and do compact letter grouping
#Using multcomp glht
summary(glht(disease_lmer, linfct=mcp(Strain="Tukey",interaction_average = T)))
```

```
## Warning in RET$pfunction("adjusted", ...): Completion with error > abseps
```

```
## 
##   Simultaneous Tests for General Linear Hypotheses
## 
## Multiple Comparisons of Means: Tukey Contrasts
## 
## 
## Fit: lme4::lmer(formula = DI ~ Strain + Strain:DPI + (1 | subject) + 
##     (1 | Batch), data = di_long_useful)
## 
## Linear Hypotheses:
##                       Estimate Std. Error z value Pr(>|z|)   
## GRS138 - GMI1000 == 0 -0.46227    0.38765  -1.193  0.88121   
## GRS447 - GMI1000 == 0  0.22651    0.97943   0.231  0.99998   
## GRS460 - GMI1000 == 0 -0.89483    0.91072  -0.983  0.95018   
## Rs517 - GMI1000 == 0  -0.90661    0.34267  -2.646  0.09560 . 
## Rs531 - GMI1000 == 0  -1.32951    0.34614  -3.841  0.00195 **
## Rs549 - GMI1000 == 0  -0.49194    0.34049  -1.445  0.74909   
## GRS447 - GRS138 == 0   0.68878    1.00205   0.687  0.99192   
## GRS460 - GRS138 == 0  -0.43256    0.93434  -0.463  0.99910   
## Rs517 - GRS138 == 0   -0.44434    0.40606  -1.094  0.91824   
## Rs531 - GRS138 == 0   -0.86724    0.40835  -2.124  0.30290   
## Rs549 - GRS138 == 0   -0.02967    0.40307  -0.074  1.00000   
## GRS460 - GRS447 == 0  -1.12134    1.29577  -0.865  0.97324   
## Rs517 - GRS447 == 0   -1.13312    0.98691  -1.148  0.89891   
## Rs531 - GRS447 == 0   -1.55602    0.98778  -1.575  0.66500   
## Rs549 - GRS447 == 0   -0.71845    0.98534  -0.729  0.98893   
## Rs517 - GRS460 == 0   -0.01178    0.91869  -0.013  1.00000   
## Rs531 - GRS460 == 0   -0.43468    0.91942  -0.473  0.99899   
## Rs549 - GRS460 == 0    0.40289    0.91639   0.440  0.99933   
## Rs531 - Rs517 == 0    -0.42290    0.36641  -1.154  0.89658   
## Rs549 - Rs517 == 0     0.41467    0.36104   1.149  0.89875   
## Rs549 - Rs531 == 0     0.83757    0.36404   2.301  0.21379   
## ---
## Signif. codes:  0 '***' 0.001 '**' 0.01 '*' 0.05 '.' 0.1 ' ' 1
## (Adjusted p values reported -- single-step method)
```

```
cld(glht(disease_lmer, linfct=mcp(Strain="Tukey", interaction_average=T)))
```

```
## GMI1000  GRS138  GRS447  GRS460   Rs517   Rs531   Rs549 
##     "a"    "ab"    "ab"    "ab"    "ab"     "b"    "ab"
```

```
#Using lmerTest lsMeans
lmerlsm <- difflsmeans(disease_lmer)$diffs.lsmeans.table
lmerlsm
```

```
##                         Estimate Standard Error     DF t-value Lower CI
## Strain GMI1000 - GRS138   2.8570         0.1943  292.8   14.70   2.4746
## Strain GMI1000 - GRS447   3.8812         0.4167 1223.9    9.31   3.0636
## Strain GMI1000 - GRS460   4.2730         0.3876 1100.9   11.03   3.5126
## Strain GMI1000 - Rs517    0.6071         0.1764  225.7    3.44   0.2594
## Strain GMI1000 - Rs531    0.0386         0.1806  254.5    0.21  -0.3170
## Strain GMI1000 - Rs549    0.5153         0.1780  235.8    2.89   0.1646
## Strain GRS138 - GRS447    1.0242         0.4235 1221.1    2.42   0.1932
## Strain GRS138 - GRS460    1.4161         0.3946 1094.0    3.59   0.6418
## Strain GRS138 - Rs517    -2.2499         0.1926  280.1  -11.68  -2.6290
## Strain GRS138 - Rs531    -2.8184         0.1964  309.6  -14.35  -3.2048
## Strain GRS138 - Rs549    -2.3417         0.1941  291.0  -12.07  -2.7236
## Strain GRS447 - GRS460    0.3919         0.5396 1230.2    0.73  -0.6668
## Strain GRS447 - Rs517    -3.2741         0.4160 1223.1   -7.87  -4.0902
## Strain GRS447 - Rs531    -3.8426         0.4177 1225.0   -9.20  -4.6621
## Strain GRS447 - Rs549    -3.3659         0.4167 1223.9   -8.08  -4.1833
## Strain GRS460 - Rs517    -3.6660         0.3867 1096.8   -9.48  -4.4248
## Strain GRS460 - Rs531    -4.2345         0.3886 1106.8  -10.90  -4.9969
## Strain GRS460 - Rs549    -3.7577         0.3875 1101.5   -9.70  -4.5181
## Strain Rs517 - Rs531     -0.5685         0.1787  241.3   -3.18  -0.9205
## Strain Rs517 - Rs549     -0.0917         0.1761  223.1   -0.52  -0.4388
## Strain Rs531 - Rs549      0.4767         0.1802  251.8    2.65   0.1218
##                         Upper CI p-value
## Strain GMI1000 - GRS138   3.2394  0.0000
## Strain GMI1000 - GRS447   4.6988  0.0000
## Strain GMI1000 - GRS460   5.0335  0.0000
## Strain GMI1000 - Rs517    0.9547  0.0007
## Strain GMI1000 - Rs531    0.3942  0.8310
## Strain GMI1000 - Rs549    0.8660  0.0041
## Strain GRS138 - GRS447    1.8551  0.0157
## Strain GRS138 - GRS460    2.1903  0.0003
## Strain GRS138 - Rs517    -1.8708  0.0000
## Strain GRS138 - Rs531    -2.4320  0.0000
## Strain GRS138 - Rs549    -1.9597  0.0000
## Strain GRS447 - GRS460    1.4505  0.4678
## Strain GRS447 - Rs517    -2.4580  0.0000
## Strain GRS447 - Rs531    -3.0231  0.0000
## Strain GRS447 - Rs549    -2.5484  0.0000
## Strain GRS460 - Rs517    -2.9072  0.0000
## Strain GRS460 - Rs531    -3.4720  0.0000
## Strain GRS460 - Rs549    -2.9973  0.0000
## Strain Rs517 - Rs531     -0.2165  0.0017
## Strain Rs517 - Rs549      0.2553  0.6029
## Strain Rs531 - Rs549      0.8317  0.0087
```

```
Comparison = str_split_fixed(rownames(lmerlsm),"Strain ",2)[,2]

### Produce compact letter display

cldList(comparison = Comparison,
        p.value    = p.adjust(lmerlsm$'p-value',
                       method =  "bonferroni")   ,
        threshold = 0.05)
```

```
##    Group Letter MonoLetter
## 1   GMI1      a       a   
## 2 GRS138      b        b  
## 3 GRS447     bc        bc 
## 4  GRS46      c         c 
## 5  Rs517      d          d
## 6  Rs531      a       a   
## 7  Rs549     ad       a  d
```

Using the linear model and raw data, different displays can be plotted, for example, a boxplot of the “Useful” data-points combined with the predictions (extrapolations) of the linear model.

```
## 
## Attaching package: 'modelr'
```

```
## The following object is masked from 'package:broom':
## 
##     bootstrap
```

```
##Add predictions to full dataset
di_long <- add_predictions(di_long,disease_lmer,var="lmer.pred")
```

```
## Warning: contrasts dropped from factor Strain
```

```
ggplot(data=di_long, aes(x=DPI,y=DI))+
  geom_boxplot(aes(color=Strain,group=DPI),data=filter(di_long, Useful=="Yes"))+
  geom_smooth(aes(y=lmer.pred,color=Strain), method="lm", alpha=0.6) +
  facet_wrap(~Strain)+
  ggtitle("Boxplots and linear fit for individual strains")
```

Finally, of the methods discussed here, those relying on a linear model of raw observations over time, can be used for other kinds of observations, e.g. bacterial titers.

At least briefly, plot residuals of the lmm to assess if this appears normal (random)

```
di_long$resid[di_long$Useful=="Yes"] <- resid(disease_lmer)
ggplot(di_long[di_long$Useful=="Yes",], aes(x=DPI,y=resid)) + geom_jitter(aes(color=Strain))
```

# Survival Analysis

## Background

Survival analysis is different in several aspects from the earlier approaches. Survival analysis, also known that time-to-event analysis, builds on a different dataset, which can be generated from raw disease indices. Survival analysis provides a way to analyze the time-to-event recordings within one population. In the case of the data used here, what is of interest is an event that can be referred to as *death*. Correctly defining *death* in this context is crucial for the outcome of the analysis. *Death*, here, is defined as a subject reaching a certain disease index, from which it cannot recover. In this script the disease index that defines the threashold to *death* is called “cutoff”.

```
###Set the cutoff by assigning a number to the variable cutoff
cutoff <- c(2.5)
```

All of the observations that are above the dotted line in the plot below are *dead*, at the day they cross that line. Those that never cross the line are alive until the end of observations, and are “right censored”, meaning that their event was not observed during the time this subject was observed.

```
ggplot(data=di_long) +
                  geom_jitter(aes(x=DPI, y=DI,color=Strain,shape=Batch)) +
                  geom_segment(aes(x=0, xend=max(DPI)+0.5,y=cutoff, yend=cutoff), linetype="dotted") +
                  labs(x = "Days post infection", y = "Disease indices", title="Scatterplot of disease indices\n cutoff plotted as dotted line") +
                  coord_cartesian(xlim=c(-0.1,10))
```

## Generation of a survival table

A “survival table” can be generated using the following code. This code works on the long table, generated in the beginning, and the cutoff variable defined above.

```
###Generate survival table
surv_from_DI <- data.frame(Subject=disease_index$subject,
                         Strain=disease_index$Strain,
                         Plant=disease_index$Plant,
                         Batch=disease_index$Batch)
###Fill survival table based on the di_long table. This generates warnings. These can be ignored and come from the min()
for (i in 1:max(di_long$subject)) { #Go by subject
  dummy <- di_long[di_long$subject==i,] #generate dummy for the subject
  if (is.infinite(min(dummy$DPI[which(dummy$DI >= cutoff)]))) { #If none of the DI is greater than the cutoff (this is where warnings are generated, min on an empty object returns infinite and a warning!)
    surv_from_DI$End[i] <- max(dummy$DPI) #Generate a  observation, censoring at the maximum DPI recorded
    surv_from_DI$Death[i] <- 0 #Still alive, because it did not pass the cutoff
  } else { #If more than zero DI are greater than the cutoff
    surv_from_DI$End[i] <- min(dummy$DPI[which(dummy$DI >= cutoff)]) #Use the lowest DPI where condition is met
    surv_from_DI$Death[i] <- 1 #record as dead
  }
}
rm(dummy)
```

## Kaplan-Meier estimates of survival

Kaplan-Meier estimates of survival are the basic tool of survival analysis. These can be estimated using the survfit function from the “survival” package.

```
library("survival")
```

```
surv_DI_fit <- survfit(Surv(End, Death) ~Strain +strata(Batch), data=surv_from_DI)
```

The survminer package provides the ggsurvplot() function. This works nicely on datasets with few treatments. However, for the data presented here, I think it is easier to initially generate a data frame that contains the whole fit and plot with ggplot2

```
library("stringr")
###Strata dummy generation, modified from kmiddleton / rexamples 
strata_dummy <-NULL
for(i in 1:length(surv_DI_fit$strata)){
      # add vector for one strata according to number of rows of strata
      strata_dummy <- c(strata_dummy, rep(names(surv_DI_fit$strata)[i], surv_DI_fit$strata[i]))
}
###Data frame generation inspired by a post by Hadley Wickham to the ggplot2 Googlegroup
surv_DI_fit.df <- data.frame( 
  time = surv_DI_fit$time, 
  n.risk = surv_DI_fit$n.risk, 
  n.event = surv_DI_fit$n.event, 
  surv = surv_DI_fit$surv, 
  strata = strata_dummy, 
  upper = surv_DI_fit$upper, 
  lower = surv_DI_fit$lower 
) 
zeros <- data.frame(time = 0, surv = 1, strata = names((surv_DI_fit$strata)), 
  upper = 1, lower = 1)

surv_DI_fit.df <- plyr::rbind.fill(zeros, surv_DI_fit.df) ###I dont want to load plyr because i guess it will interfere with dplyr...
rm(strata_dummy)
rm(zeros)
###Some stuff to rename other stuff, this needs to be adapted if other variables are used.

surv_DI_fit.df$Batch <- as.factor( str_split_fixed(
  matrix( nrow=length(surv_DI_fit.df$strata),ncol=2, unlist(strsplit(as.character(surv_DI_fit.df$strata),", ")), byrow=T )[,2],"=",2)[,2])
surv_DI_fit.df$Strain <- as.factor( str_split_fixed(
    matrix( nrow=length(surv_DI_fit.df$strata),ncol=2, unlist(strsplit(as.character(surv_DI_fit.df$strata),", ")), byrow=T )[,1],"=",2)[,2])

###End of data frame generation
###Start plotting
ggplot(surv_DI_fit.df, aes(time, surv, colour = Strain)) + 
  geom_step(aes(y = surv*100,linetype=Batch)) +
  facet_wrap(~Strain) +
  ggtitle("Survival estimates for all Batches")
```

## Cox-Proportional hazards and hazard ratios

Different approaches to survival analysis, are based on analysing the hazards. The hazard is the probability of experiencing an event at a given timepoint. Many hazard based analysis assume that hazards are proportional between treatments, meaning that they differ by a fixed factor. Hazards were strongly influenced by Cox and a basic model is the cox proportional hazards model.

```
###Cox-Proportional hazards####
#Build model
srv_coxph <- coxph(Surv(End, Death) ~Strain + strata(Batch), data=surv_from_DI)
```

```
## Warning in fitter(X, Y, strats, offset, init, control, weights = weights, :
## Loglik converged before variable 2 ; beta may be infinite.
```

```
###Check porportionality of hazards
cox.zph(srv_coxph, transform = "log")
```

```
##                   rho    chisq       p
## StrainGRS138  0.19054 1.01e+01 0.00147
## StrainGRS447 -0.24174 2.45e-07 0.99961
## StrainGRS460  0.09105 2.41e+00 0.12022
## StrainRs517   0.11162 3.87e+00 0.04904
## StrainRs531   0.03187 3.17e-01 0.57322
## StrainRs549   0.00393 4.86e-03 0.94441
## GLOBAL             NA 1.48e+01 0.02202
```

This dataset is not suitable for this analysis. Beta is probably infinte because two strains have no events, hence no hazard.

```
library("survcomp")
library("rms")
library("coxme")
```

```
####Hazard ratio
haz_rats <- hazard.ratio(x= surv_from_DI$Strain, 
                         surv.time = surv_from_DI$End, 
                         surv.event = surv_from_DI$Death, 
                         strat = surv_from_DI$Batch, 
                         method.test = "wald" ) ###Overall hazard ratios
```

```
## Warning in fitter(X, Y, strats, offset, init, control, weights = weights, :
## Loglik converged before variable 2 ; beta may be infinite.
```

```
###Pairwise hazard ratios / modified from the pairwise chisq calculation
pw_hazrats <- matrix(0., nlevels(surv_from_DI$Strain),nlevels(surv_from_DI$Strain))
for (i in 1:nlevels(surv_from_DI$Strain)) {
  for (j in (1:nlevels(surv_from_DI$Strain))[-i]) {
    datasubset <- droplevels(subset( surv_from_DI,
      surv_from_DI$Strain %in% (unique(surv_from_DI$Strain))[c(i,j)]))
    temp <- hazard.ratio(
      x= datasubset$Strain, 
      surv.time = datasubset$End, 
      surv.event = datasubset$Death, 
      strat = datasubset$Batch, 
      method.test = "likelihood.ratio" ###Define test to determine p.
      )
    pw_hazrats[i,j] <- temp$p.value
    }
}
```

```
## Warning in fitter(X, Y, strats, offset, init, control, weights = weights, :
## Loglik converged before variable 1 ; beta may be infinite.
```

```
## Warning in fitter(X, Y, strats, offset, init, control, weights = weights, :
## Loglik converged before variable 1 ; beta may be infinite.

## Warning in fitter(X, Y, strats, offset, init, control, weights = weights, :
## Loglik converged before variable 1 ; beta may be infinite.

## Warning in fitter(X, Y, strats, offset, init, control, weights = weights, :
## Loglik converged before variable 1 ; beta may be infinite.

## Warning in fitter(X, Y, strats, offset, init, control, weights = weights, :
## Loglik converged before variable 1 ; beta may be infinite.

## Warning in fitter(X, Y, strats, offset, init, control, weights = weights, :
## Loglik converged before variable 1 ; beta may be infinite.

## Warning in fitter(X, Y, strats, offset, init, control, weights = weights, :
## Loglik converged before variable 1 ; beta may be infinite.

## Warning in fitter(X, Y, strats, offset, init, control, weights = weights, :
## Loglik converged before variable 1 ; beta may be infinite.

## Warning in fitter(X, Y, strats, offset, init, control, weights = weights, :
## Loglik converged before variable 1 ; beta may be infinite.

## Warning in fitter(X, Y, strats, offset, init, control, weights = weights, :
## Loglik converged before variable 1 ; beta may be infinite.

## Warning in fitter(X, Y, strats, offset, init, control, weights = weights, :
## Loglik converged before variable 1 ; beta may be infinite.

## Warning in fitter(X, Y, strats, offset, init, control, weights = weights, :
## Loglik converged before variable 1 ; beta may be infinite.

## Warning in fitter(X, Y, strats, offset, init, control, weights = weights, :
## Loglik converged before variable 1 ; beta may be infinite.

## Warning in fitter(X, Y, strats, offset, init, control, weights = weights, :
## Loglik converged before variable 1 ; beta may be infinite.

## Warning in fitter(X, Y, strats, offset, init, control, weights = weights, :
## Loglik converged before variable 1 ; beta may be infinite.

## Warning in fitter(X, Y, strats, offset, init, control, weights = weights, :
## Loglik converged before variable 1 ; beta may be infinite.

## Warning in fitter(X, Y, strats, offset, init, control, weights = weights, :
## Loglik converged before variable 1 ; beta may be infinite.

## Warning in fitter(X, Y, strats, offset, init, control, weights = weights, :
## Loglik converged before variable 1 ; beta may be infinite.
```

```
colnames(pw_hazrats) <- levels(surv_from_DI$Strain)
rownames(pw_hazrats) <- levels(surv_from_DI$Strain)
```

```
stargazer::stargazer(pw_hazrats,type="html",title="Pairwise hazard ratio pvalues")
```

**Pairwise hazard ratio pvalues**

|  | | | | | | | |
|  | GMI1000 | GRS138 | GRS447 | GRS460 | Rs517 | Rs531 | Rs549 |
|  | | | | | | | |
| GMI1000 | 0 | 0 | 0 | 0 | 0.103 | 0.0002 | 0.008 |
| GRS138 | 0 | 0 | 0 | 0 | 0 | 0 | 0 |
| GRS447 | 0 | 0 | 0 | 0.096 | 0 | 0 | 0 |
| GRS460 | 0 | 0 | 0.096 | 0 | 0 | 0 | 0 |
| Rs517 | 0.103 | 0 | 0 | 0 | 0 | 0.032 | 0.277 |
| Rs531 | 0.0002 | 0 | 0 | 0 | 0.032 | 0 | 0.370 |
| Rs549 | 0.008 | 0 | 0 | 0 | 0.277 | 0.370 | 0 |
|  | | | | | | | |

As stated above, these warnings confirm that there are non proportional hazards.

```
cme <-  coxme(Surv(End, Death) ~Strain + (1|Batch), data=surv_from_DI)
```

```
## Warning in coxfitfun(x, y, strata = strata, offset = offset, init =
## ifixed, : Loglik converged before variable 2 ; beta may be infinite.
```

```
anova(cme)
```

```
## Analysis of Deviance Table
##  Cox model: response is Surv(End, Death)
## Terms added sequentially (first to last)
## 
##         loglik  Chisq Df Pr(>|Chi|)    
## NULL   -1764.6                         
## Strain -1470.7 587.78  6  < 2.2e-16 ***
## ---
## Signif. codes:  0 '***' 0.001 '**' 0.01 '*' 0.05 '.' 0.1 ' ' 1
```

```
summary(glht(cme,linfct=mcp(Strain="Tukey")))
```

```
## 
##   Simultaneous Tests for General Linear Hypotheses
## 
## Multiple Comparisons of Means: Tukey Contrasts
## 
## 
## Fit: coxme(formula = Surv(End, Death) ~ Strain + (1 | Batch), data = surv_from_DI)
## 
## Linear Hypotheses:
##                        Estimate Std. Error z value Pr(>|z|)    
## GRS138 - GMI1000 == 0   -2.5808     0.2245 -11.498   <0.001 ***
## GRS447 - GMI1000 == 0  -24.9601  9796.2653  -0.003   1.0000    
## GRS460 - GMI1000 == 0   -5.8716     0.7269  -8.078   <0.001 ***
## Rs517 - GMI1000 == 0    -0.8147     0.1771  -4.600   <0.001 ***
## Rs531 - GMI1000 == 0    -0.3232     0.1735  -1.862   0.4304    
## Rs549 - GMI1000 == 0    -0.5413     0.1756  -3.083   0.0228 *  
## GRS447 - GRS138 == 0   -22.3793  9796.2653  -0.002   1.0000    
## GRS460 - GRS138 == 0    -3.2908     0.7259  -4.534   <0.001 ***
## Rs517 - GRS138 == 0      1.7661     0.2138   8.260   <0.001 ***
## Rs531 - GRS138 == 0      2.2577     0.2219  10.173   <0.001 ***
## Rs549 - GRS138 == 0      2.0395     0.2183   9.343   <0.001 ***
## GRS460 - GRS447 == 0    19.0885  9796.2653   0.002   1.0000    
## Rs517 - GRS447 == 0     24.1454  9796.2653   0.002   1.0000    
## Rs531 - GRS447 == 0     24.6369  9796.2653   0.003   1.0000    
## Rs549 - GRS447 == 0     24.4188  9796.2653   0.002   1.0000    
## Rs517 - GRS460 == 0      5.0569     0.7230   6.995   <0.001 ***
## Rs531 - GRS460 == 0      5.5485     0.7259   7.644   <0.001 ***
## Rs549 - GRS460 == 0      5.3303     0.7245   7.358   <0.001 ***
## Rs531 - Rs517 == 0       0.4915     0.1770   2.777   0.0571 .  
## Rs549 - Rs517 == 0       0.2734     0.1788   1.529   0.6633    
## Rs549 - Rs531 == 0      -0.2181     0.1761  -1.239   0.8407    
## ---
## Signif. codes:  0 '***' 0.001 '**' 0.01 '*' 0.05 '.' 0.1 ' ' 1
## (Adjusted p values reported -- single-step method)
```

## Analysis of survival curves and fits

Comparing the Kaplan-Meier survival estimates can be done in different ways.

### Logrank testing

The below produces all pairwise comparisons of the Kaplan Meier estimate of survival using a logrank test.

```
###Make a table of pairwise chisq pvalues, for the logrank test.
#Based on a post to the R Mailing list by T. Therneau
pw_logrank_test_type <- 0 ###0 for logrank, 1 for peto and peto
pw_logrank <- matrix(0., nlevels(surv_from_DI$Strain),nlevels(surv_from_DI$Strain))
for (i in 1:nlevels(surv_from_DI$Strain)) {
  for (j in (1:nlevels(surv_from_DI$Strain))[-i]) {
    datasubset <- droplevels(subset( surv_from_DI,
      surv_from_DI$Strain %in% (unique(surv_from_DI$Strain))[c(i,j)]))
    temp <- survdiff(Surv(End, Death)~Strain+strata(Batch), data=datasubset, rho=pw_logrank_test_type)
      
    pw_logrank[i,j] <- pchisq(temp$chisq, df=1, lower=F) ##df will always be 1 because this is pairwise
    }
}
colnames(pw_logrank) <- levels(surv_from_DI$Strain)
rownames(pw_logrank) <- levels(surv_from_DI$Strain)
#Make dummy adjustment table
pw_logrank_adjBon <- pw_logrank
#Fill adjusted pvalue table.
for (i in 1:ncol(pw_logrank)) {
  pw_logrank_adjBon[,i] <- cbind(p.adjust(pw_logrank[,i], method="bonferroni"))
}
```

```
stargazer::stargazer(pw_logrank_adjBon,type="html",title="Pairwise Chisq p-values (Bonferroni adjusted)")
```

**Pairwise Chisq p-values (Bonferroni adjusted)**

|  | | | | | | | |
|  | GMI1000 | GRS138 | GRS447 | GRS460 | Rs517 | Rs531 | Rs549 |
|  | | | | | | | |
| GMI1000 | 0 | 0 | 0 | 0 | 0.112 | 0.00005 | 0.012 |
| GRS138 | 0 | 0 | 0 | 0 | 0 | 0 | 0 |
| GRS447 | 0 | 0 | 0 | 1 | 0 | 0 | 0 |
| GRS460 | 0 | 0 | 1 | 0 | 0 | 0 | 0 |
| Rs517 | 0.112 | 0 | 0 | 0 | 0 | 0.106 | 1 |
| Rs531 | 0.00005 | 0 | 0 | 0 | 0.106 | 0 | 1 |
| Rs549 | 0.012 | 0 | 0 | 0 | 1 | 1 | 0 |
|  | | | | | | | |

### Regressions

Generally a survival regression does not assume proportionality of hazards. A survival regression is fit to a distribution, defined by dist=“”.

```
####Survival Regression###
###This is done using functions from rms.
###psm is a survival::survreg wrapper. but the output is more handle-able.
library("modelr")
ddist <- datadist(surv_from_DI)
options(datadist="ddist")
psurv_gaus <- psm(Surv(End, Death) ~Strain, data=surv_from_DI, dist="gaussian")
psurv_logistic <- psm(Surv(End, Death) ~Strain, data=surv_from_DI, dist="logistic")
psurv_lnorm <- psm(Surv(End, Death) ~Strain, data=surv_from_DI, dist="lognormal")
psurv_wei <- psm(Surv(End, Death) ~Strain, data=surv_from_DI, dist="weibull")

###Same with survreg()
s_reg_gaus <-     survreg(Surv(End, Death) ~Strain, data=surv_from_DI, dist="gaussian")
s_reg_logistic <- survreg(Surv(End, Death) ~Strain, data=surv_from_DI, dist="logistic")
s_reg_lnorm <-    survreg(Surv(End, Death) ~Strain, data=surv_from_DI, dist="lognormal")
s_reg_wei <-      survreg(Surv(End, Death) ~Strain, data=surv_from_DI, dist="weibull")  

aic.scores.psurv <- rbind(
  extractAIC(s_reg_wei),
  extractAIC(s_reg_gaus),
  extractAIC(s_reg_logistic),
  extractAIC(s_reg_lnorm))
###Make useable AIC table
rownames(aic.scores.psurv) <- c("Weibull", "Gaussian", "Logist", "Lognorm")
colnames(aic.scores.psurv) <- c("df", "AIC")
###Call table
```

```
stargazer::stargazer(aic.scores.psurv,type="html",title="AIC Scores")
```

**AIC Scores**

|  | | |
|  | df | AIC |
|  | | |
| Weibull | 8 | 1,304.774 |
| Gaussian | 8 | 1,309.638 |
| Logist | 8 | 1,297.576 |
| Lognorm | 8 | 1,240.430 |
|  | | |

From the table above, the model with the lowest AIC score can be chosen. For this analysis, this is the lognormal model, but this does not have to apply to other experiments. Then, one can inspect that model for significance.

```
summary(glht(psurv_lnorm,linfct=mcp(Strain="Tukey")))
```

```
## 
##   Simultaneous Tests for General Linear Hypotheses
## 
## Multiple Comparisons of Means: Tukey Contrasts
## 
## 
## Fit: psm(formula = Surv(End, Death) ~ Strain, data = surv_from_DI, 
##     dist = "lognormal")
## 
## Linear Hypotheses:
##                        Estimate Std. Error z value Pr(>|z|)    
## GRS138 - GMI1000 == 0   0.64838    0.04598  14.100  < 0.001 ***
## GRS447 - GMI1000 == 0   2.30074  307.30774   0.007  1.00000    
## GRS460 - GMI1000 == 0   1.18658    0.08717  13.612  < 0.001 ***
## Rs517 - GMI1000 == 0    0.17362    0.04403   3.944  0.00115 ** 
## Rs531 - GMI1000 == 0    0.07757    0.04404   1.761  0.51578    
## Rs549 - GMI1000 == 0    0.11539    0.04407   2.618  0.09444 .  
## GRS447 - GRS138 == 0    1.65236  307.30773   0.005  1.00000    
## GRS460 - GRS138 == 0    0.53820    0.08718   6.174  < 0.001 ***
## Rs517 - GRS138 == 0    -0.47476    0.04598 -10.324  < 0.001 ***
## Rs531 - GRS138 == 0    -0.57080    0.04599 -12.411  < 0.001 ***
## Rs549 - GRS138 == 0    -0.53299    0.04601 -11.585  < 0.001 ***
## GRS460 - GRS447 == 0   -1.11416  307.30774  -0.004  1.00000    
## Rs517 - GRS447 == 0    -2.12712  307.30774  -0.007  1.00000    
## Rs531 - GRS447 == 0    -2.22317  307.30774  -0.007  1.00000    
## Rs549 - GRS447 == 0    -2.18535  307.30774  -0.007  1.00000    
## Rs517 - GRS460 == 0    -1.01296    0.08717 -11.620  < 0.001 ***
## Rs531 - GRS460 == 0    -1.10901    0.08716 -12.724  < 0.001 ***
## Rs549 - GRS460 == 0    -1.07119    0.08714 -12.293  < 0.001 ***
## Rs531 - Rs517 == 0     -0.09605    0.04404  -2.181  0.25603    
## Rs549 - Rs517 == 0     -0.05823    0.04407  -1.321  0.80640    
## Rs549 - Rs531 == 0      0.03782    0.04409   0.858  0.97159    
## ---
## Signif. codes:  0 '***' 0.001 '**' 0.01 '*' 0.05 '.' 0.1 ' ' 1
## (Adjusted p values reported -- single-step method)
```

Again, one can then inspect the differences in the model, for example using pairwise comparisons of means.

```
pairwise_confint_sreg <- as.data.frame(confint(glht(psurv_lnorm, mcp(Strain = "Tukey")))$confint)
pairwise_confint_sreg$Comparison <- rownames(pairwise_confint_sreg)
###Plot the comparisons, below may not be the most straight-foward way to plot this the way I want it, but it works.
ggplot(pairwise_confint_sreg, aes(x = Comparison, y = Estimate, ymin = lwr, ymax = upr, color = Comparison)) + ###Plot Comparison on x, estimate on y
  scale_x_discrete(limits = rev(levels(as.factor(pairwise_confint$Comparison)))) + ###Rescale x, so the order is inverted 
  geom_errorbar() + geom_point() + ###Draw data
  coord_flip() +  theme(legend.position="none") + xlab("") +###Invert X and Y, hide legend
  ggtitle("Difference in means with 95% Confidence interval") ##Add a title
```

In this dataset, the confidence intervals are quite huge for certain comparisons. This is because some strains are avirulent. Including these in this kind of analysis does not make much sense, similar to the hazard analysis above.

### Plotting of parametric survival regression

It is possible, but not really easy, to plot the generated curves. These curves are the result of fitting the data to a distribution in the earlier section. Doing this in a manner that is compatible with ggplot2 is not straightforward. Below is code to generate plots of the KM estimates per batch and the generated regression. This is performed for the four distributions above, and can be adapted to different distributions if necessary.

```
###Step 1, extract the coefficients. These are relative to Strain1 because Strain is treatment contrasted.

for (i in 1:nlevels(surv_DI_fit.df$Strain)) { #For loop through strains
  if(i==1) { #Strain1 is relative to itself, so no change
  coef_wei <- list()
  coef_logistic <- list()
  coef_gaus <- list()
  coef_lnorm <- list()
  coef_wei[i] <- coef(s_reg_wei)[i]
  coef_logistic[i] <- coef(s_reg_logistic)[i]
  coef_gaus[i] <- coef(s_reg_gaus)[i]
  coef_lnorm[i] <- coef(s_reg_lnorm)[i]
  } else { ###Other strains are relative to 1
  coef_wei[i] <- coef(s_reg_wei)[1] + coef(s_reg_wei)[i]
  coef_logistic[i] <- coef(s_reg_logistic)[1] + coef(s_reg_logistic)[i]
  coef_gaus[i] <- coef(s_reg_gaus)[1] + coef(s_reg_gaus)[i]
  coef_lnorm[i] <- coef(s_reg_lnorm)[1] + coef(s_reg_lnorm)[i]
  }
}
##Step 2
####Store the coefficients and the scale in a new data frame, of parameters
### Keep in mind that survreg.distributions$weibull is different from rweibull, hence the difference in names.
sregparams <- data.frame(
  Strain = rep(levels(surv_from_DI$Strain),4 ), #Fill with strains
  scale.wei = exp(unlist(coef_wei)), #weibull fit scale parameters
  scale.logistic = rep(s_reg_logistic$scale, nlevels(surv_from_DI$Strain)), #fill with logis scales
  scale.gaus = rep(s_reg_gaus$scale, nlevels(surv_from_DI$Strain)), #fill with gaus scales
  scale.lnorm = rep(s_reg_lnorm$scale, nlevels(surv_from_DI$Strain)), #fill with lnorm scale
  shape.wei =  rep(1/s_reg_wei$scale, nlevels(surv_from_DI$Strain)), #shape for weibull
  shape.logistic = unlist(coef_logistic), #shape for logistic
  shape.gaus =  unlist(coef_gaus), #shape for gaus
  shape.lnorm =   unlist(coef_lnorm) #shape for lnorm
  )
##Step 3
###Calculate the "daily" value of each curve
for (i in 1:nlevels(surv_DI_fit.df$Strain)){
  if(i==1) {
    wei <- list()
    logis <- list()
    gaus <- list()
    lnorm <- list()
  }
  x <- levels(surv_DI_fit.df$Strain)[i]
  n <- c(1:max(surv_from_DI$End))
  data <- filter(sregparams, Strain==x)
  time <- n
  wei <- cbind(wei, pweibull(
    q=n,
    scale=data$scale.wei,
    shape=data$shape.wei,
    lower.tail=FALSE))
  logis <- cbind(logis,plogis(
    q=n,
    scale=data$scale.logistic,
    location=data$shape.logistic,
    lower.tail=FALSE  ))
  gaus <- cbind(gaus,pnorm(
   q=n,
   sd=data$scale.gaus,
   mean=data$shape.gaus,
   lower.tail = F))
  lnorm <- cbind(lnorm, plnorm(
    q=n,
    sd=data$scale.lnorm,
    mean=data$shape.lnorm,
    lower.tail=F))
}

##Step 4
###Put all the curves into a data.frame that contains information on "time" and also "Strain", for compatibility with other data.frames
sreg_curves <- data.frame(
  wei.sreg = cbind(unlist(wei)),
  logis.sreg = cbind(unlist(logis)),
  gaus.sreg = cbind(unlist(gaus)),
  lnorm.sreg = cbind(unlist(lnorm)),
  Strain = rep(unlist(levels(surv_DI_fit.df$Strain)),each=max(surv_from_DI$End)),
  time = rep(c(1:max(surv_from_DI$End)), nlevels(surv_DI_fit.df$Strain))
)
##Step 5
###Turn that data.frame into a long data.frame (not used here but for other figures.)
sreg_long <- sreg_curves %>% gather(., key="Distribution",values = c(lnorm.sreg, wei.sreg,gaus.sreg,logis.sreg) )
sreg_long$Distribution <- as.factor(sreg_long$Distribution)
##Levels: gaus.sreg lnorm.sreg logis.sreg wei.sreg
levels(sreg_long$Distribution) <- c("Gaussian","Lognormal","Loglogistic","Weibull")
```

Now, these can be plotted and inspected visually.

```
###Plot of KM+Weibull
  ggplot(surv_DI_fit.df, aes(time, surv, colour = Strain)) +
  geom_step(aes(linetype=Batch)) +
  geom_line(data=sreg_curves,aes(y=wei.sreg),color="black") +
  facet_wrap(~Strain) + 
  ggtitle("Kaplan-Meier estimates and fit to\nWeibull distribution")
```

```
###Plot of KM+Logistic
  ggplot(surv_DI_fit.df, aes(time, surv, colour = Strain)) +
    geom_step(aes(linetype=Batch)) +
    geom_line(data=sreg_curves,aes(y=logis.sreg),color="black") +
    facet_wrap(~Strain) + 
    ggtitle("Kaplan-Meier estimates and fit to\n Logistic distribution")
```

```
###Plot of KM+Gaussian  
  ggplot(surv_DI_fit.df, aes(time, surv, colour = Strain)) +
    geom_step(aes(linetype=Batch)) +
    geom_line(data=sreg_curves,aes(y=gaus.sreg),color="black") +
    facet_wrap(~Strain) + 
    ggtitle("Kaplan-Meier estimates and fit to\nGaussian distribution")
```

```
###Plot of KM+Lognormal  
  ggplot(surv_DI_fit.df, aes(time, surv, colour = Strain)) +
    geom_step(aes(linetype=Batch)) +
    geom_line(data=sreg_curves,aes(y=lnorm.sreg),color="black") +
    facet_wrap(~Strain) + 
    ggtitle("Kaplan-Meier estimates and fit to\nLognormal distribution")
```

# Comparison of analyis method results.

An inherent question when analyzing data, is which analysis produced which result and why. Below, the outputs from the three major analysis performed are printed, so they can be compared.

```
summary(auc_lmer)
```

```
## Linear mixed model fit by REML t-tests use Satterthwaite approximations
##   to degrees of freedom [lmerMod]
## Formula: AUC ~ Strain + (1 | Batch)
##    Data: auc_df
## 
## REML criterion at convergence: 2822.2
## 
## Scaled residuals: 
##     Min      1Q  Median      3Q     Max 
## -4.5888 -0.3924  0.0755  0.4577  3.0258 
## 
## Random effects:
##  Groups   Name        Variance Std.Dev.
##  Batch    (Intercept)  2.606   1.614   
##  Residual             22.092   4.700   
## Number of obs: 476, groups:  Batch, 5
## 
## Fixed effects:
##              Estimate Std. Error       df t value Pr(>|t|)    
## (Intercept)   23.5530     0.9199   8.8000  25.603 1.38e-09 ***
## StrainGRS138 -15.9816     0.8061 465.0000 -19.826  < 2e-16 ***
## StrainGRS447 -23.2059     0.8061 465.0000 -28.789  < 2e-16 ***
## StrainGRS460 -22.9412     0.8061 465.0000 -28.460  < 2e-16 ***
## StrainRs517   -3.1581     0.8061 465.0000  -3.918 0.000103 ***
## StrainRs531   -1.2059     0.8061 465.0000  -1.496 0.135337    
## StrainRs549   -2.6691     0.8061 465.0000  -3.311 0.001001 ** 
## ---
## Signif. codes:  0 '***' 0.001 '**' 0.01 '*' 0.05 '.' 0.1 ' ' 1
## 
## Correlation of Fixed Effects:
##             (Intr) SGRS13 SGRS44 SGRS46 StR517 StR531
## StranGRS138 -0.438                                   
## StranGRS447 -0.438  0.500                            
## StranGRS460 -0.438  0.500  0.500                     
## StrainRs517 -0.438  0.500  0.500  0.500              
## StrainRs531 -0.438  0.500  0.500  0.500  0.500       
## StrainRs549 -0.438  0.500  0.500  0.500  0.500  0.500
```

```
summary(disease_lmer)
```

```
## Linear mixed model fit by REML t-tests use Satterthwaite approximations
##   to degrees of freedom [lmerMod]
## Formula: DI ~ Strain + Strain:DPI + (1 | subject) + (1 | Batch)
##    Data: di_long_useful
## 
## REML criterion at convergence: 3846.9
## 
## Scaled residuals: 
##     Min      1Q  Median      3Q     Max 
## -4.3707 -0.5894  0.0215  0.6755  2.5070 
## 
## Random effects:
##  Groups   Name        Variance Std.Dev.
##  subject  (Intercept) 0.7443   0.8627  
##  Batch    (Intercept) 0.1899   0.4357  
##  Residual             0.7897   0.8887  
## Number of obs: 1248, groups:  subject, 476; Batch, 5
## 
## Fixed effects:
##                     Estimate Std. Error         df t value Pr(>|t|)    
## (Intercept)         -2.04347    0.29924   19.60000  -6.829 1.39e-06 ***
## StrainGRS138        -0.46227    0.38765 1228.90000  -1.193 0.233295    
## StrainGRS447         0.22651    0.97943 1088.30000   0.231 0.817153    
## StrainGRS460        -0.89483    0.91072 1211.40000  -0.983 0.326025    
## StrainRs517         -0.90661    0.34267 1227.60000  -2.646 0.008255 ** 
## StrainRs531         -1.32951    0.34614 1199.20000  -3.841 0.000129 ***
## StrainRs549         -0.49194    0.34049 1223.50000  -1.445 0.148770    
## StrainGMI1000:DPI    0.82182    0.03645  593.60000  22.549  < 2e-16 ***
## StrainGRS138:DPI     0.44806    0.03356  977.50000  13.351  < 2e-16 ***
## StrainGRS447:DPI     0.18070    0.08933 1011.00000   2.023 0.043352 *  
## StrainGRS460:DPI     0.29456    0.08334 1165.20000   3.534 0.000425 ***
## StrainRs517:DPI      0.86857    0.03845  793.60000  22.590  < 2e-16 ***
## StrainRs531:DPI      1.02331    0.04310  620.40000  23.740  < 2e-16 ***
## StrainRs549:DPI      0.81817    0.03947  936.60000  20.727  < 2e-16 ***
## ---
## Signif. codes:  0 '***' 0.001 '**' 0.01 '*' 0.05 '.' 0.1 ' ' 1
```

```
## 
## Correlation matrix not shown by default, as p = 14 > 12.
## Use print(x, correlation=TRUE)  or
##   vcov(x)     if you need it
```

```
summary(psurv_lnorm)
```

```
##              Effects              Response : Surv(End, Death) 
## 
##  Factor                  Low High Diff. Effect   S.E.       Lower 0.95  
##  Strain - GRS138:GMI1000 1   2    NA    0.648380   0.045985   5.5801e-01
##   Survival Time Ratio    1   2    NA    1.912400         NA   1.7472e+00
##  Strain - GRS447:GMI1000 1   3    NA    2.300700 307.310000  -6.0157e+02
##   Survival Time Ratio    1   3    NA    9.981500         NA  5.4972e-262
##  Strain - GRS460:GMI1000 1   4    NA    1.186600   0.087173   1.0153e+00
##   Survival Time Ratio    1   4    NA    3.275900         NA   2.7601e+00
##  Strain - Rs517:GMI1000  1   5    NA    0.173620   0.044026   8.7106e-02
##   Survival Time Ratio    1   5    NA    1.189600         NA   1.0910e+00
##  Strain - Rs531:GMI1000  1   6    NA    0.077572   0.044040  -8.9696e-03
##   Survival Time Ratio    1   6    NA    1.080700         NA   9.9107e-01
##  Strain - Rs549:GMI1000  1   7    NA    0.115390   0.044073   2.8783e-02
##   Survival Time Ratio    1   7    NA    1.122300         NA   1.0292e+00
##  Upper 0.95 
##   7.3874e-01
##   2.0933e+00
##   6.0617e+02
##  1.8124e+263
##   1.3579e+00
##   3.8879e+00
##   2.6013e-01
##   1.2971e+00
##   1.6411e-01
##   1.1783e+00
##   2.0200e-01
##   1.2238e+00
```

An easier comparisons might be accomplished with the compact letter display.

```
cld(glht(auc_lmer, linfct=mcp(Strain="Tukey")))
```

```
## GMI1000  GRS138  GRS447  GRS460   Rs517   Rs531   Rs549 
##     "d"     "b"     "a"     "a"     "c"    "cd"     "c"
```

```
#Compact letters for lmerTest objects are a little tricky. This solution comes from the rcompanion.
### Extract lsmeans table

lmerlsm <- difflsmeans(disease_lmer)$diffs.lsmeans.table

Comparison = str_split_fixed(rownames(lmerlsm),"Strain ",2)[,2]

### Produce compact letter display

library(rcompanion)

cldList(comparison = Comparison,
        p.value    = p.adjust(lmerlsm$'p-value',
                       method =  "bonferroni")   ,
        threshold = 0.05)
```

```
##    Group Letter MonoLetter
## 1   GMI1      a       a   
## 2 GRS138      b        b  
## 3 GRS447     bc        bc 
## 4  GRS46      c         c 
## 5  Rs517      d          d
## 6  Rs531      a       a   
## 7  Rs549     ad       a  d
```

```
cld(glht(srv_coxph,linfct=mcp(Strain="Tukey")))
```

```
## GMI1000  GRS138  GRS447  GRS460   Rs517   Rs531   Rs549 
##     "a"     "c"  "abcd"     "d"     "b"    "ab"     "b"
```

```
cld(glht(psurv_lnorm,linfct=mcp(Strain="Tukey")))
```

```
## GMI1000  GRS138  GRS447  GRS460   Rs517   Rs531   Rs549 
##     "a"     "c"  "abcd"     "d"     "b"    "ab"    "ab"
```

```
cld(glht(cme,linfct=mcp(Strain="Tukey")))
```

```
## GMI1000  GRS138  GRS447  GRS460   Rs517   Rs531   Rs549 
##     "a"     "c"  "abcd"     "d"     "b"    "ab"     "b"
```

# Session Info

```
sessionInfo()
```

```
## R version 3.3.3 (2017-03-06)
## Platform: x86_64-w64-mingw32/x64 (64-bit)
## Running under: Windows 10 x64 (build 14393)
## 
## locale:
## [1] LC_COLLATE=English_United States.1252 
## [2] LC_CTYPE=English_United States.1252   
## [3] LC_MONETARY=English_United States.1252
## [4] LC_NUMERIC=C                          
## [5] LC_TIME=English_United States.1252    
## 
## attached base packages:
## [1] stats     graphics  grDevices utils     datasets  methods   base     
## 
## other attached packages:
##  [1] coxme_2.2-5      bdsmatrix_1.3-2  rms_5.1-0        SparseM_1.74    
##  [5] Hmisc_4.0-2      Formula_1.2-1    lattice_0.20-34  survcomp_1.24.0 
##  [9] prodlim_1.5.9    modelr_0.1.0     stringr_1.2.0    rcompanion_1.5.0
## [13] multcomp_1.4-6   TH.data_1.0-8    MASS_7.3-45      survival_2.40-1 
## [17] mvtnorm_1.0-5    lmerTest_2.0-33  lme4_1.1-12      Matrix_1.2-8    
## [21] MESS_0.4-3       geepack_1.2-1    ggplot2_2.2.1    dplyr_0.5.0     
## [25] broom_0.4.2      tidyr_0.6.1     
## 
## loaded via a namespace (and not attached):
##  [1] survivalROC_1.0.3    nlme_3.1-131         pbkrtest_0.4-7      
##  [4] ordinal_2015.6-28    RColorBrewer_1.1-2   rprojroot_1.2       
##  [7] tools_3.3.3          backports_1.0.5      R6_2.2.0            
## [10] KernSmooth_2.23-15   rpart_4.1-10         rmeta_2.16          
## [13] nortest_1.0-4        DBI_0.5-1            lazyeval_0.2.0      
## [16] mgcv_1.8-17          colorspace_1.3-2     ade4_1.7-5          
## [19] nnet_7.3-12          gridExtra_2.2.1      mnormt_1.5-5        
## [22] quantreg_5.29        htmlTable_1.9        hermite_1.1.1       
## [25] expm_0.999-1         sandwich_2.3-4       labeling_0.3        
## [28] scales_0.4.1         checkmate_1.8.2      polspline_1.1.12    
## [31] lmtest_0.9-35        psych_1.6.12         mc2d_0.1-18         
## [34] multcompView_0.1-7   digest_0.6.12        foreign_0.8-67      
## [37] minqa_1.2.4          rmarkdown_1.3        base64enc_0.1-3     
## [40] WRS2_0.9-1           htmltools_0.3.5      manipulate_1.0.1    
## [43] htmlwidgets_0.8      SuppDists_1.1-9.4    zoo_1.7-14          
## [46] acepack_1.4.1        car_2.1-4            magrittr_1.5        
## [49] modeltools_0.2-21    Rcpp_0.12.9          DescTools_0.99.19   
## [52] munsell_0.4.3        ucminf_1.1-4         stringi_1.1.2       
## [55] yaml_2.1.14          plyr_1.8.4           grid_3.3.3          
## [58] parallel_3.3.3       stargazer_5.2        splines_3.3.3       
## [61] knitr_1.15.1         EMT_1.1              boot_1.3-18         
## [64] reshape2_1.4.2       codetools_0.2-15     stats4_3.3.3        
## [67] evaluate_0.10        latticeExtra_0.6-28  data.table_1.10.4   
## [70] nloptr_1.0.4         bootstrap_2017.2     miscTools_0.6-22    
## [73] MatrixModels_0.4-1   gtable_0.2.0         purrr_0.2.2         
## [76] reshape_0.8.6        assertthat_0.1       coin_1.1-3          
## [79] BSDA_1.01            tibble_1.2           lava_1.4.7          
## [82] cluster_2.0.5        maxLik_1.3-4         RVAideMemoire_0.9-63
```

```
