# Supplementary material for: A Practical Guide to Visualization and Statistical Analysis of R. solanacearum Infection Data Using R
Source: Front Plant Sci. 2017 Apr 24;8:623. doi: 10.3389/fpls.2017.00623 (PMC5401893; doi:10.3389/fpls.2017.00623)
Supplement: Supplementary file 3 [file Data_Sheet_3.ZIP › S3_Allen_Lab_Section_I.html]

Approaches to analyze experimental R. solanacearum infections: Supplementary Material 3: Datasets from the Allen Lab Part I: Data import, and formatting


# Approaches to analyze experimental *R. solanacearum* infections: *Supplementary Material 3:* Datasets from the Allen Lab Part I: Data import, and formatting

#### *Niklas Schandry*

# General information on this document

This document is part of the supplementary material of “A practical guide to descriptive and statistical analysis of R. solanacearum infection data using R”. In this document, datasets from Lowe, et al., MPMI, 2015 and Lowe-Power, et al., mBio, 2016 are prepared for analysis. Each dataset is analyzed seperately in part II and III, respectively.

# Data Type

The disease index score is based the severity of symptoms. A symptom read-out (usually “% wilted leaves”) is turned into a numeric score. This score ranges from 0-4, and each number is used to reflect a certain interval of disease severity. The value of the disease index can never be below 0, or above 4. Therefore, the disease index is not a continuous response variable, but is ordinal.

# Data Import

## Specifying the data

Specify directory (if running from console..)

```
###Install all required packages:
#install.packages(c("tidyr","dplyr","ggplot2","MESS","lme4","lmerTest","multcomp","survival","psm","coxme","stargazer","survcomp","rms","modelr","party","stringr"))


###Define Working Directory and set it
###Note for the Markdown version: R-Markdown cannot set the working directory
###R markdown will always use the directory the .Rmd file is located in
###In the .Rmd file this code section is not actually evaluated and only serves illustratory purpose.
wd <- c("~/My_Data/DataDirectory/")
setwd(wd)
```

Specify tables.

```
###Name of the file to be read
mpmi2015 <- c("S3_LOWE_MPMI_2015.csv") 
mBio2016 <- c("S3_Lowe-Power_mBio_2016.csv")
```

These datasets from the research group of Caitilyn Allen at University of Wisconsin have previously been published.

## Reading data and formatting

The data can be read into *R* and stored in a data frame. I am calling that data frame disease.index

```
di_mpmi <- as.data.frame(read.table(mpmi2015, header=T,
                                          sep=";" , ###Sets the seperator of the csv file
                                          dec=","), ###Sets the decimal operator of the csv file
                                          stringsAsFactors=F) ###
di_mbio <- as.data.frame(read.table(mBio2016, header=T,
                                          sep=";" , ###Sets the seperator of the csv file
                                          dec=","), ###Sets the decimal operator of the csv file
                                          stringsAsFactors=F) ###
```

Using str() one can see if the tables were properly imported.

```
str(di_mpmi)
```

```
## 'data.frame':    164 obs. of  19 variables:
##  $ DPI   : int  1 2 3 4 5 6 7 8 9 10 ...
##  $ A     : int  0 0 0 0 0 0 1 2 3 4 ...
##  $ B     : int  0 0 0 0 0 2 4 4 4 4 ...
##  $ C     : int  0 0 0 0 2 4 4 4 4 4 ...
##  $ D     : int  0 0 0 0 0 0 0 0 0 0 ...
##  $ E     : int  0 0 0 0 0 0 2 4 3 4 ...
##  $ F     : int  0 0 0 0 0 1 1 4 4 4 ...
##  $ G     : int  0 0 0 0 0 4 4 4 4 4 ...
##  $ H     : int  0 0 0 0 0 0 0 0 1 2 ...
##  $ I     : int  0 0 0 0 0 3 4 4 4 4 ...
##  $ J     : int  0 0 0 2 3 4 4 4 4 4 ...
##  $ K     : int  0 0 0 0 0 0 0 0 2 3 ...
##  $ L     : int  0 0 0 0 0 0 4 4 4 4 ...
##  $ M     : int  0 0 0 0 1 3 4 4 4 4 ...
##  $ N     : int  0 0 0 0 0 0 1 3 3 4 ...
##  $ O     : int  NA NA NA NA NA NA NA NA NA NA ...
##  $ P     : int  NA NA NA NA NA NA NA NA NA NA ...
##  $ Strain: Factor w/ 3 levels "","GMI1000","GMI1000_fcsmut": 2 2 2 2 2 2 2 2 2 2 ...
##  $ Batch : int  1 1 1 1 1 1 1 1 1 1 ...
```

```
str(di_mbio)
```

```
## 'data.frame':    280 obs. of  13 variables:
##  $ DPI   : int  1 2 3 4 5 6 7 8 9 10 ...
##  $ A     : int  0 0 0 0 0 0 0 0 0 0 ...
##  $ B     : int  0 0 0 0 0 0 0 1 3 4 ...
##  $ C     : int  0 0 0 0 0 1 2 4 4 4 ...
##  $ D     : int  0 0 0 0 0 1 1 4 4 4 ...
##  $ E     : int  0 0 0 0 0 0 0 0 0 0 ...
##  $ F     : int  0 0 0 0 0 0 0 0 0 0 ...
##  $ G     : int  0 0 0 0 0 0 0 0 0 0 ...
##  $ H     : int  0 0 0 0 0 0 0 0 0 0 ...
##  $ I     : int  0 0 0 3 4 4 4 4 4 4 ...
##  $ J     : int  0 0 0 0 0 0 0 0 0 0 ...
##  $ Strain: Factor w/ 5 levels "comp_nagGH","comp_nagOP",..: 5 5 5 5 5 5 5 5 5 5 ...
##  $ Batch : int  1 1 1 1 1 1 1 1 1 1 ...
```

These tables need to be made long.

```
library("tidyr")
```

```
mpmi2015_long <- gather(di_mpmi, key=Plant, 
                     value=DI,
                     c(A, B, C, D, E, F ,G ,H ,I ,J, K, L, M ,N, O, P), 
                     na.rm=F)
mbio2016_long <- gather(di_mbio, key=Plant, 
                     value=DI,
                     c(A, B, C, D, E, F ,G ,H ,I ,J), 
                     na.rm=F)
```

Now, it is time to generate the “subject” variable, unique for each subject. A unique identifier can be generated by combining information from Strain, Plant and Batch.

```
mpmi2015_long$subject <- interaction(mpmi2015_long$Strain,
                                     mpmi2015_long$Plant,
                                     mpmi2015_long$Batch)
mbio2016_long$subject <- interaction(mbio2016_long$Strain,
                                     mbio2016_long$Plant,
                                     mbio2016_long$Batch)
###Factorize batch information
mpmi2015_long$Batch <- as.factor(mpmi2015_long$Batch)
mbio2016_long$Batch <- as.factor(mbio2016_long$Batch)
```

## Defining the contrasts

A crucial step that will influence all outputs of the statistical analysis is setting the “contrasts”. Contrasts specifies the “baseline” for each of the variables. “Treatment” contrasts specify that the first alphabetical level will be used as a reference for all others (see Strain below), while a “sum” constrast means that the reference value is the mean across all levels of that variable.

```
####Specify what should be "appropriate" contrasts
contrasts(mpmi2015_long$Strain) <- "contr.Treatment"###First alphabetical strain will be the baseline
contrasts(mbio2016_long$Strain) <- "contr.Treatment"###First alphabetical strain will be the baseline

contrasts(mpmi2015_long$Batch) <- "contr.sum" ###Batches will be averaged to generate the baseline!
contrasts(mbio2016_long$Batch) <- "contr.sum"
```

## Adding a censoring variable

The below adds the variable that defines observations that are relevant for disease development.

```
##Censoring for both datasets.

di_long <- na.omit(mpmi2015_long)
###Slightly modified with as.numeric
oldw <- getOption("warn")
options(warn = -1)
###Interesting R-related observation, the below does not work when subsetting is done with filter(), 
###because filter does not retain rownames!
for (i in 1:max(as.numeric(di_long$subject))) { ###Go by subject
  dummy1 <- di_long[as.numeric(di_long$subject)==i,] ##Create a first dummy object, that is a subset of the full data containing the current subject 
  if(min(dummy1$DI) == 0){
    #remove those observations that are before disease onset, except the one directly before disease onset.
    dummy1 <- dummy1[dummy1$DPI %in% (max(dummy1$DPI[dummy1$DI==0]):max(dummy1$DPI)),]
  } 
  if (max(dummy1$DI) == 4) { ###If this subject dies at some point
    dummy2 <- dummy1[dummy1$DI==4,] ###Make a new dummy object, that only contains those recordings where DI=4
    NEW <- di_long[as.numeric(di_long$subject)==i & (di_long$DPI %in% min(dummy1$DPI):min(dummy2$DPI)),] ###Generate data subset "NEW", which contains those observations for a subject, that are between (including) the last recording where DI=0 and the first recording where disease index is 4.
  } else { ###If this dubject does not die
    NEW <- dummy1 ###New is the same as dummy1
  }
  di_long$Useful[rownames(di_long) %in% rownames(NEW)] <- c("Yes") ###All of those row(names) that are part of the "NEW" object are useful. Therefore these receive status "Yes" in column "Useful"
}
di_long$Useful[which(is.na(di_long$Useful))] <- c("No") ###Those that are not yes (and therefore are NA) become No
rm(dummy1,dummy2)

mpmi2015_long <- di_long


##Same for mBio dataset
di_long <- na.omit(mbio2016_long)

###Interesting R-related observation, the below does not work when subsetting is done with filter(), 
###because filter does not retain rownames!
for (i in 1:max(as.numeric(di_long$subject))) { ###Go by subject
  dummy1 <- di_long[as.numeric(di_long$subject)==i,] ##Create a first dummy object, that is a subset of the full data containing the current subject 
  if(min(dummy1$DI) == 0){
    #remove those observations that are before disease onset, except the one directly before disease onset.
    dummy1 <- dummy1[dummy1$DPI %in% (max(dummy1$DPI[dummy1$DI==0]):max(dummy1$DPI)),]
  } 
  if (max(dummy1$DI) == 4) { ###If this subject dies at some point
    dummy2 <- dummy1[dummy1$DI==4,] ###Make a new dummy object, that only contains those recordings where DI=4
    NEW <- di_long[as.numeric(di_long$subject)==i & (di_long$DPI %in% min(dummy1$DPI):min(dummy2$DPI)),] ###Generate data subset "NEW", which contains those observations for a subject, that are between (including) the last recording where DI=0 and the first recording where disease index is 4.
  } else { ###If this dubject does not die
    NEW <- dummy1 ###New is the same as dummy1
  }
  di_long$Useful[rownames(di_long) %in% rownames(NEW)] <- c("Yes") ###All of those row(names) that are part of the "NEW" object are useful. Therefore these receive status "Yes" in column "Useful"
}
di_long$Useful[which(is.na(di_long$Useful))] <- c("No") ###Those that are not yes (and therefore are NA) become No
rm(dummy1,dummy2)
mbio2016_long <- di_long
options(warn = oldw)
```

## Table quality check

```
library("broom")
```

```
str(mpmi2015_long)
```

```
## 'data.frame':    2216 obs. of  7 variables:
##  $ DPI    : int  1 2 3 4 5 6 7 8 9 10 ...
##  $ Strain : Factor w/ 3 levels "","GMI1000","GMI1000_fcsmut": 2 2 2 2 2 2 2 2 2 2 ...
##   ..- attr(*, "contrasts")= chr "contr.Treatment"
##  $ Batch  : Factor w/ 6 levels "1","2","3","4",..: 1 1 1 1 1 1 1 1 1 1 ...
##   ..- attr(*, "contrasts")= chr "contr.sum"
##  $ Plant  : chr  "A" "A" "A" "A" ...
##  $ DI     : int  0 0 0 0 0 0 1 2 3 4 ...
##  $ subject: Factor w/ 288 levels ".A.1","GMI1000.A.1",..: 2 2 2 2 2 2 2 2 2 2 ...
##  $ Useful : chr  "No" "No" "No" "No" ...
##  - attr(*, "na.action")=Class 'omit'  Named int [1:408] 161 162 163 164 325 326 327 328 489 490 ...
##   .. ..- attr(*, "names")= chr [1:408] "161" "162" "163" "164" ...
```

```
tidy(mpmi2015_long) ###This can be used to assess the descriptive statistics of the data.
```

```
## Warning: NAs introduced by coercion

## Warning: NAs introduced by coercion
```

```
## Warning in FUN(newX[, i], ...): no non-missing arguments to min; returning
## Inf

## Warning in FUN(newX[, i], ...): no non-missing arguments to min; returning
## Inf
```

```
## Warning in FUN(newX[, i], ...): no non-missing arguments to max; returning
## -Inf

## Warning in FUN(newX[, i], ...): no non-missing arguments to max; returning
## -Inf
```

```
##     column    n       mean         sd median    trimmed      mad min  max
## 1      DPI 2216   7.283394  3.9354859      7   7.251973   4.4478   1   14
## 2  Strain* 2216   2.490975  0.5000314      2   2.488726   0.0000   2    3
## 3   Batch* 2216   3.469314  1.6069703      4   3.461669   1.4826   1    6
## 4   Plant* 2216        NaN         NA     NA        NaN       NA Inf -Inf
## 5       DI 2216   1.965704  1.9246991      2   1.957159   2.9652   0    4
## 6 subject* 2216 141.037906 78.3927509    148 142.549605 100.8168   2  270
## 7  Useful* 2216        NaN         NA     NA        NaN       NA Inf -Inf
##   range        skew  kurtosis         se
## 1    13  0.03461304 -1.174779 0.08360139
## 2     1  0.03608253 -1.999600 0.01062215
## 3     5 -0.12466164 -1.101610 0.03413681
## 4  -Inf          NA        NA         NA
## 5     4  0.03913449 -1.934082 0.04088631
## 6   268 -0.18866940 -1.149750 1.66529442
## 7  -Inf          NA        NA         NA
```

```
str(mbio2016_long)
```

```
## 'data.frame':    2800 obs. of  7 variables:
##  $ DPI    : int  1 2 3 4 5 6 7 8 9 10 ...
##  $ Strain : Factor w/ 5 levels "comp_nagGH","comp_nagOP",..: 5 5 5 5 5 5 5 5 5 5 ...
##   ..- attr(*, "contrasts")= chr "contr.Treatment"
##  $ Batch  : Factor w/ 4 levels "1","2","3","4": 1 1 1 1 1 1 1 1 1 1 ...
##   ..- attr(*, "contrasts")= chr "contr.sum"
##  $ Plant  : chr  "A" "A" "A" "A" ...
##  $ DI     : int  0 0 0 0 0 0 0 0 0 0 ...
##  $ subject: Factor w/ 200 levels "comp_nagGH.A.1",..: 5 5 5 5 5 5 5 5 5 5 ...
##  $ Useful : chr  "No" "No" "No" "No" ...
```

```
tidy(mbio2016_long) ###This can be used to assess the descriptive statistics of the data.
```

```
## Warning: NAs introduced by coercion
```

```
## Warning: NAs introduced by coercion
```

```
## Warning in FUN(newX[, i], ...): no non-missing arguments to min; returning
## Inf

## Warning in FUN(newX[, i], ...): no non-missing arguments to min; returning
## Inf
```

```
## Warning in FUN(newX[, i], ...): no non-missing arguments to max; returning
## -Inf

## Warning in FUN(newX[, i], ...): no non-missing arguments to max; returning
## -Inf
```

```
##     column    n       mean        sd median    trimmed     mad min  max
## 1      DPI 2800   7.500000  4.031849    7.5   7.500000  5.1891   1   14
## 2  Strain* 2800   3.000000  1.414466    3.0   3.000000  1.4826   1    5
## 3   Batch* 2800   2.500000  1.118234    2.5   2.500000  1.4826   1    4
## 4   Plant* 2800        NaN        NA     NA        NaN      NA Inf -Inf
## 5       DI 2800   1.556786  1.877546    0.0   1.445982  0.0000   0    4
## 6 subject* 2800 100.500000 57.744618  100.5 100.500000 74.1300   1  200
## 7  Useful* 2800        NaN        NA     NA        NaN      NA Inf -Inf
##   range      skew  kurtosis         se
## 1    13 0.0000000 -1.213584 0.07619478
## 2     4 0.0000000 -1.301214 0.02673090
## 3     3 0.0000000 -1.361171 0.02113263
## 4  -Inf        NA        NA         NA
## 5     4 0.4611584 -1.718249 0.03548228
## 6   199 0.0000000 -1.201345 1.09127070
## 7  -Inf        NA        NA         NA
```

# Table export

```
write.csv(mbio2016_long, file = "S3_mBio2016_long.csv")
write.csv(mpmi2015_long, file = "S3_mpmi2015_long.csv")
```

# Session Info

```
sessionInfo()
```

```
## R version 3.3.2 (2016-10-31)
## Platform: x86_64-w64-mingw32/x64 (64-bit)
## Running under: Windows 10 x64 (build 14393)
## 
## locale:
## [1] LC_COLLATE=English_United States.1252 
## [2] LC_CTYPE=English_United States.1252   
## [3] LC_MONETARY=English_United States.1252
## [4] LC_NUMERIC=C                          
## [5] LC_TIME=English_United States.1252    
## 
## attached base packages:
## [1] stats     graphics  grDevices utils     datasets  methods   base     
## 
## other attached packages:
## [1] broom_0.4.2 tidyr_0.6.1
## 
## loaded via a namespace (and not attached):
##  [1] Rcpp_0.12.9     knitr_1.15.1    magrittr_1.5    mnormt_1.5-5   
##  [5] lattice_0.20-34 R6_2.2.0        stringr_1.2.0   plyr_1.8.4     
##  [9] dplyr_0.5.0     tools_3.3.2     parallel_3.3.2  grid_3.3.2     
## [13] nlme_3.1-131    psych_1.6.12    DBI_0.5-1       htmltools_0.3.5
## [17] yaml_2.1.14     lazyeval_0.2.0  assertthat_0.1  rprojroot_1.2  
## [21] digest_0.6.12   tibble_1.2      reshape2_1.4.2  evaluate_0.10  
## [25] rmarkdown_1.3   stringi_1.1.2   backports_1.0.5 foreign_0.8-67
```
