# Supplementary material for: A Practical Guide to Visualization and Statistical Analysis of R. solanacearum Infection Data Using R
Source: Front Plant Sci. 2017 Apr 24;8:623. doi: 10.3389/fpls.2017.00623 (PMC5401893; doi:10.3389/fpls.2017.00623)
Supplement: Supplementary file 3 [file Data_Sheet_3.ZIP › S3_Allen_Lab_Section_II.html]

Approaches to analyze experimental R. solanacearum infections: Supplementary Material 3: Datasets from the Allen Lab Part II: Analysis of Lowe\_MPMI\_2015


# Approaches to analyze experimental *R. solanacearum* infections: *Supplementary Material 3:* Datasets from the Allen Lab Part II: Analysis of Lowe\_MPMI\_2015

#### *Niklas Schandry*

# General information on this document

This document is part of the supplementary material of “A practical guide to descriptive and statistical analysis of R. solanacearum infection data using R”. In this document, the analysis of the Lowe\_MPMI\_2015 dataset is performed, based on the formatted file generated in Part I.

# Data Import

## Specifying the data

The below is retained so packages can be installed if necessary.

```
###Install all required packages:
#install.packages(c("MESS","lme4","lmerTest","multcomp","survival","rms","coxme","stargazer","survcomp","tidyverse","rcompanion"))


###Define Working Directory and set it
###Note for the Markdown version: R-Markdown cannot set the working directory
###R markdown will always use the directory the .Rmd file is located in
###In the .Rmd file this code section is not actually evaluated and only serves illustratory purpose.
wd <- c("~/My_Data/DataDirectory/")
setwd(wd)
```

Import the mpmi2015 long table. This was generated in section I of the S3 material.

```
###Name of the file to be read
di_long <- read.table("S3_mpmi2015_long.csv", sep=",", header=T)
di_long$DPI <- as.numeric(di_long$DPI)
###Redifine the variables
di_long$Batch <- as.factor(di_long$Batch)
contrasts(di_long$Strain) <- "contr.treatment"###First alphabetical strain will be the baseline!
contrasts(di_long$Batch) <- "contr.sum" ###Batches will be averaged to generate the baseline!
```

# Analysis of AUDPC

## Rationale and data preparation

Everything from here on out is very similar to the analysis performed on sim\_data.csv. *Change* in below code: Removed “Plant” from group\_by, since Plant here has a different meaning than in sim\_data.csv (here it is a batch specific subject, in the other dataset it is plant genotype)

```
library("dplyr")
```

```
di_summary <- di_long %>% group_by(Strain, Batch, DPI) %>% 
  summarise(mean(DI),sd(DI),sd(DI)/sqrt(length(DI))) ##calculate within Batch mean, sd, and se for each Plant/Strain combination.
##Averages within each replicate. This summary table is mainly helpful for plotting, not used for analysis...
colnames(di_summary) <- c("Strain", "Batch", "DPI", "mean", "sd", "se") ###Assign correct columnnames
```

Using these summaries, one can take a look at the averaged disease progression, using

```
library("ggplot2")
```

```
ggplot(filter(di_summary, Batch==di_summary$Batch[[1]])) + ###Use Only Batch A (too busy otherwise)
  aes(x=DPI,y=mean,color=Strain) + ###Color by Strain, specify x and y.
  geom_area(aes(fill=Strain),position="identity",alpha=0.15) + ###Area plot, colored by Strain
  ##geom_errorbar(aes(x=DPI, ymax=mean+se, ymin=mean-se), size=0.25)+ ###This line adds SE. 
  ##geom_errorbar(aes(x=DPI, ymax=mean+sd, ymin=mean-sd), size=0.25)+ ###This line adds SD. Don't use both.
  facet_wrap(~Strain) + ###One plot per strain 
  labs(x = "Days post infection", y = "Avg. Disease Index") + #Labels
  ggtitle("Figure 1\nDisease Areas,\nper strain, for batch A") #Title
```

From this plot, one can see that in the example dataset the areas differ quite drastically between different strains. As can be seen here, all observations can be included in an AUDCP analysis, but one should take care that total observation times are similar, identical if possible. As the area increases with both, increased disease index and prolonged time, experiments of different length should not be compared using this approach.

To calculated the actual AUDPC for each individual in the dataset a new data frame is created. As AUDCP is calculated from both disease index and DPI, this can not be stored in a reasonable way in the long data frame generated earlier.

*Change*: Modified subject selection to include as.numeric(). Here, the subjects are based on interaction, hence have character names. Each level of this corresponds to a number, so this can be used for iteration in the loop below.

```
library("MESS")
```

```
####Build a table of AUDCPs, per subject
auc_df <- data.frame() ###Make auc_df data frame
for (i in 1:max(as.numeric(di_long$subject))) { ##Go by subject
  temp <- di_long[as.numeric(di_long$subject)==i,] ###Subset full table into the subject table
  temp <- droplevels(temp) ###Drop levels, so levels works properly below
  auc_df[i,1] <- i ###Subject number
  auc_df[i,2] <- levels(temp$Strain) ###Strain
  auc_df[i,3] <- levels(temp$Plant) ###Plant
  auc_df[i,4] <- levels(temp$Batch) ###Batch
  auc_df[i,5] <- auc(temp$DPI,temp$DI) ###AUC; i assume that trapezoid rule is fine here. 
  ### Additionally, auc calculation starts with the lowest x (DPI). I think this is sensible
  ### I assume that if one specifies "from=0", the curve is expanded by a triangle that covers the range from 
  ### 0 to whatever is the value at the first observation. I think the first observation should ideally be 0
  ### if data was recorded from the beginning..
}
colnames(auc_df) <- c("subject","Strain","Plant","Batch","AUC") ###Name columns in AUC datafarame
auc_df$Strain <- as.factor(auc_df$Strain) #refactor
auc_df$Plant <- as.factor(auc_df$Plant) #refactor
auc_df$Batch <- as.factor(auc_df$Batch) #refactor
str(auc_df)
```

```
## 'data.frame':    164 obs. of  5 variables:
##  $ subject: int  1 2 3 4 5 6 7 8 9 10 ...
##  $ Strain : Factor w/ 2 levels "GMI1000","GMI1000_fcsmut": 1 1 1 1 1 1 1 1 1 1 ...
##  $ Plant  : Factor w/ 16 levels "A","B","C","D",..: 1 1 1 1 1 1 2 2 2 2 ...
##  $ Batch  : Factor w/ 6 levels "1","2","3","4",..: 1 2 3 4 5 6 1 2 3 4 ...
##  $ AUC    : num  24 24 30 35 18 24 32 18 35 39 ...
```

The auc data.frame contains one area under the curve per subject and all other subject specific variables as stored in the original table.

## Analysis of differences in area under the disease progression curve

An initial assessment of strain specific differences in AUDPC can be performed visually, for example by generating boxpots.

```
ggplot(auc_df) + geom_boxplot(
  aes(x=Strain, y=AUC, color=Strain),  #Plot boxplots of AUCs, by strains
  notch=F) +
  labs(y="AUDPC") +
  facet_wrap(~Batch) + ###Individual plots per batch (and plant if applicable)
  ggtitle("Figure 2\nArea Under the Disease Progression Curve per strain across batches")
```

Next, one can use the area under the disease progression curve, to build a linear model, or a linear mixed effects model.

```
library("lme4")
library("lmerTest")
```

```
summary(lm(AUC~Batch,data=auc_df)) ###Can be used to identify batch effects. If there are none, including batch as a random factor below is not necessary (but also not necessarily wrong).
```

```
## 
## Call:
## lm(formula = AUC ~ Batch, data = auc_df)
## 
## Residuals:
##     Min      1Q  Median      3Q     Max 
## -28.812  -5.278   1.900   7.074  15.900 
## 
## Coefficients:
##             Estimate Std. Error t value Pr(>|t|)    
## (Intercept)   25.893      1.876  13.804  < 2e-16 ***
## Batch2        -9.793      2.906  -3.370 0.000945 ***
## Batch3         2.107      2.568   0.820 0.413231    
## Batch4         2.920      2.568   1.137 0.257375    
## Batch5         1.045      2.568   0.407 0.684766    
## Batch6        -9.793      2.906  -3.370 0.000945 ***
## ---
## Signif. codes:  0 '***' 0.001 '**' 0.01 '*' 0.05 '.' 0.1 ' ' 1
## 
## Residual standard error: 9.926 on 158 degrees of freedom
## Multiple R-squared:  0.2064, Adjusted R-squared:  0.1813 
## F-statistic: 8.218 on 5 and 158 DF,  p-value: 6.38e-07
```

```
auc_lmer <- lmer(
  AUC ~ Strain + (1|Batch),   ### AUC modeled as a function of strain, random effects of batch 
    data=auc_df  ) #Linear mixed effects model.

auc_lm <- lm(AUC~Strain+Batch,data=auc_df) #Linear model.

AIC(auc_lm,auc_lmer) #Lower AIC, better fit, linear model is slightly better.
```

```
##          df      AIC
## auc_lm    8 1224.508
## auc_lmer  4 1228.903
```

```
ggplot(data=auc_df, aes(y=AUC, x=Strain)) +geom_boxplot(aes(colour=Batch)) + ggtitle("Boxplot of AUDCP per strain by experimental batch")
```

Also in this dataset, effects from Replication are observed. Batches 4 and 6 performed poorly.

The model can be explored using various functions, such as summary.

```
library("broom")
summary(auc_lmer)  ### A model summary, containing information on the model.
```

```
## Linear mixed model fit by REML t-tests use Satterthwaite approximations
##   to degrees of freedom [lmerMod]
## Formula: AUC ~ Strain + (1 | Batch)
##    Data: auc_df
## 
## REML criterion at convergence: 1220.9
## 
## Scaled residuals: 
##     Min      1Q  Median      3Q     Max 
## -3.0237 -0.6276  0.1797  0.7031  1.6521 
## 
## Random effects:
##  Groups   Name        Variance Std.Dev.
##  Batch    (Intercept) 30.33    5.507   
##  Residual             97.05    9.851   
## Number of obs: 164, groups:  Batch, 6
## 
## Fixed effects:
##                      Estimate Std. Error      df t value Pr(>|t|)    
## (Intercept)            25.201      2.502   5.900  10.071 6.17e-05 ***
## StrainGMI1000_fcsmut   -2.866      1.539 156.840  -1.863   0.0644 .  
## ---
## Signif. codes:  0 '***' 0.001 '**' 0.01 '*' 0.05 '.' 0.1 ' ' 1
## 
## Correlation of Fixed Effects:
##             (Intr)
## StrGMI1000_ -0.307
```

```
tidy(auc_lmer) ### A cleaner display using tidy.
```

```
##                      term  estimate std.error statistic    group
## 1             (Intercept) 25.201072  2.502316 10.071101    fixed
## 2    StrainGMI1000_fcsmut -2.865854  1.538501 -1.862757    fixed
## 3    sd_(Intercept).Batch  5.507005        NA        NA    Batch
## 4 sd_Observation.Residual  9.851212        NA        NA Residual
```

```
###The tidy output explained:
#Term: A discription: (Intercept) overall intercept. Intercept depends on the contrasts set initially. Here treatment contrasts are used, so Intercept = First alphabetical strain (Strain1).
#StrainStrain2: Difference in the estimate (slope), between Strain2 and the (Intercept)

##Estimate: The estimated slope
```

For example, we see the estimated slopes (Estimate) and standard errors, together with a t- and corresponding p-value in the output of the summary function. Note, that the above only contains information on differences between different levels and the “baseline”“, which is called (Intercept). The baseline is determined by the contrast settings that were specified earlier.

But, it may be quite relevant to know how individual strains compare to each other (and not just how each strain compares to Strain1). This can be analyzed using a generalized linear hypothesis test, while ajusting for multiple comparisons using Tukey’s method.

```
library("multcomp")
library("rcompanion")
library("stringr")
```

```
tidy(summary(glht(auc_lmer, linfct=mcp(Strain="Tukey"))))
```

```
##                        lhs rhs  estimate std.error statistic    p.value
## 1 GMI1000_fcsmut - GMI1000   0 -2.865854  1.538501 -1.862757 0.06249641
```

This information can, for example, be integrated into a boxplot of the individual disease areas. Using a compact letter display, in conbination with AUDPC, combines statistical and visual information.

```
auc_cld <- cld(glht(auc_lmer, linfct=mcp(Strain="Tukey"))) ###Save letters
auc_cld <- cbind(levels(auc_df$Strain),auc_cld$mcletters$Letters) ###bind letters to columns
colnames(auc_cld) <- c("Strain","Letter") ###Name columns
auc_cld <- as.data.frame(auc_cld) #Coerce to dataframe
###Integrate letters into auc_df##
auc_df <- left_join(auc_df,auc_cld,by="Strain",copy=T) ###Add letter information
###Some extra scripting to make the mean and CI plot.
auc_CI <- as.data.frame(tidy(confint(auc_lmer)))
```

```
## Computing profile confidence intervals ...
```

```
auc_CI <- auc_CI[3:nrow(auc_CI),] ##Drop sig01, sigma
auc_CI$Strain <- levels(auc_df$Strain)
###Mean relative to Strain1 (except strain1 that one is absolute)

for (i in 1:nrow(auc_CI)){
  if (i==1){
    auc_CI$mean[i] <- mean(c(auc_CI$X2.5..[i],auc_CI$X97.5..[i]))
    auc_CI$upr[i] <- auc_CI$X97.5..[i]
    auc_CI$lwr[i] <- auc_CI$X2.5..[i]
  } else {
    auc_CI$mean[i] <- c(auc_CI$mean[1]+mean(c(auc_CI$X2.5..[i],auc_CI$X97.5..[i])))
    auc_CI$upr[i] <- c(auc_CI$mean[1]+auc_CI$X97.5..[i])
    auc_CI$lwr[i] <- c(auc_CI$mean[1]+auc_CI$X2.5..[i])
  }
}
 
 
####Generate plot of meanCI of AUDCP with significance letters and raw data as jittered points
  ggplot(aes(x=Strain, y=AUC, color=Strain),data=auc_df) + ###Plot the auc_df
  geom_crossbar(data = auc_CI, aes(x = Strain, y = mean, ymin = lwr, ymax = upr,fill=Strain), alpha=0.3) +
  geom_jitter(aes(shape=Batch)) + ###with jitter overplotted, symbol shape defined by batch
  geom_text(aes(x=Strain, y=-3, label=Letter),color="black", data=auc_cld) + ###Get the letters from auc_cld 
  #and write those to position y=-3
  labs(y="AUDPC") + #Y-Axis label
  ggtitle("AUDPC raw values and mean from the LMM\nwith 95% CI per strain and grouping letters") #Title
```

One can plot the pairwise difference in AUDPC means with confidence intervals for the linear model and the linear mixed effects model.

```
pairwise_confint_AUDPC_lm <- as.data.frame(confint(glht(auc_lm, mcp(Strain = "Tukey")))$confint)
pairwise_confint_AUDPC_lm$Comparison <- rownames(pairwise_confint_AUDPC_lm)

pairwise_confint_AUDPC_lmer <- as.data.frame(confint(glht(auc_lmer, mcp(Strain = "Tukey")))$confint)
pairwise_confint_AUDPC_lmer$Comparison <- rownames(pairwise_confint_AUDPC_lmer)

###Plot the comparisons, below may not be the most straight-foward way to plot this the way I want it, but it works.
ggplot(pairwise_confint_AUDPC_lm, aes(x = Comparison, y = Estimate, ymin = lwr, ymax = upr, color = abs(Estimate))) + ###Plot Comparison on x, estimate on y
  scale_x_discrete(limits = rev(levels(as.factor(pairwise_confint_AUDPC_lm$Comparison)))) + ###Rescale x, so the order is inverted 
  geom_errorbar() + geom_point() + ###Draw data
  coord_flip() +  theme(legend.position="none") + xlab("") +###Invert X and Y, hide legend
  ggtitle("Difference in means of the AUDPC \nin the linear model with 95% confidence interval") ##Add a title
```

```
####Plot of the comparisons in the LMM. 
ggplot(pairwise_confint_AUDPC_lmer, aes(x = Comparison, y = Estimate, ymin = lwr, ymax = upr, color = abs(Estimate))) + ###Plot Comparison on x, estimate on y
  scale_x_discrete(limits = rev(levels(as.factor(pairwise_confint_AUDPC_lmer$Comparison)))) + ###Rescale x, so the order is inverted 
  geom_errorbar() + geom_point() + ###Draw data
  coord_flip() +  theme(legend.position="none") + xlab("") +###Invert X and Y, hide legend
  ggtitle("Difference in means of the AUDPC \nin the LMM with 95% confidence interval") ##Add a title
```

Estimates of lm and lmm are very similar. As a rule of thumb, comparisons where “0” is not part of the 95% confidence interval are likely to produce a signficant p-value (assuming significance is denoted by p<0.05).

# Analysis of Disease development

## Using a Repeated Measure ANOVA.

Repeated-measure ANOVA can be used to analyze DI and time. However, when using repeated measure ANOVA one should be aware that the arrow of time is not considered in this analysis. The way aov seems to implement RM anova, the variable denoting the measurements is put into the Error() term.

```
#Change contrasts, see explanation in the main text. Contr.sum corresponds to  effect coding.
contrasts(di_long$Strain) <- "contr.sum"
rm_aov <- aov(DI~Strain + Error(DPI), data = di_long)
summary(rm_aov)
```

```
## 
## Error: DPI
##        Df Sum Sq Mean Sq
## Strain  1   4797    4797
## 
## Error: Within
##             Df Sum Sq Mean Sq F value   Pr(>F)    
## Strain       1     30  29.576   19.37 1.13e-05 ***
## Residuals 2213   3379   1.527                     
## ---
## Signif. codes:  0 '***' 0.001 '**' 0.01 '*' 0.05 '.' 0.1 ' ' 1
```

```
###I personally think that LMMs are nicer to investigate and offer greater flexibility.
```

## Using a linear mixed effect model.

### Rationale

In this section, the data is analyzed using a linear mixed effect model. While such models have been used already in the previous section, to test for a strain specific influence on the area under the disease progression curve, different data is used to build the model in this section. As mentioned, AUDPC summarizes disease incidence and time into a single variable, the area. However, in certain cases the AUDCP could be very similar, while the actual disease progression is different. Other methods may be more sensitive to such differences. Below a different approach using linear mixed effect models is taken.

### Model

The main difference between this analysis and the one based on the AUDPC is the response variable. In the AUDPC analysis the area was the response variable. Here in this section, the response variable is the Disease Index, and time is included as predictor (covariate) in the model. A linear mixed effects model is generated. Here, the Disease index is modeled on the fixed effects “Day post infection” and “Strain”.

```
###Define contrasts for lmer
contrasts(di_long$Strain) <- "contr.treatment"###First alphabetical strain will be the baseline!
#contrasts(di_long$Plant) <- "contr.treatment"###First alphabetical plant will be the baseline!
contrasts(di_long$Batch) <- "contr.poly" ###Batches will be averaged
###Drop things that are not "Useful"
di_long.useful <- filter(di_long, Useful=="Yes")
str(di_long.useful)
```

```
## 'data.frame':    515 obs. of  8 variables:
##  $ X      : int  6 7 8 9 10 21 22 23 33 34 ...
##  $ DPI    : num  6 7 8 9 10 7 8 9 5 6 ...
##  $ Strain : Factor w/ 2 levels "GMI1000","GMI1000_fcsmut": 1 1 1 1 1 2 2 2 1 1 ...
##   ..- attr(*, "contrasts")= chr "contr.treatment"
##  $ Batch  : Factor w/ 6 levels "1","2","3","4",..: 1 1 1 1 1 1 1 1 2 2 ...
##   ..- attr(*, "contrasts")= chr "contr.poly"
##  $ Plant  : Factor w/ 16 levels "A","B","C","D",..: 1 1 1 1 1 1 1 1 1 1 ...
##  $ DI     : int  0 1 2 3 4 0 3 4 0 2 ...
##  $ subject: Factor w/ 164 levels "GMI1000.A.1",..: 1 1 1 1 1 83 83 83 2 2 ...
##  $ Useful : Factor w/ 2 levels "No","Yes": 2 2 2 2 2 2 2 2 2 2 ...
```

```
## Build linear mixed effect model

disease_lmer <- lmer(DI ~ Strain + Strain:DPI + (1| subject) + (1 | Batch), di_long.useful)
```

The model can be investigated using summary functions. The pairwise comparisons may be plotted with 95%CI to assess how different two strains are.

```
###Check model summary
summary(disease_lmer)
```

```
## Linear mixed model fit by REML t-tests use Satterthwaite approximations
##   to degrees of freedom [lmerMod]
## Formula: DI ~ Strain + Strain:DPI + (1 | subject) + (1 | Batch)
##    Data: di_long.useful
## 
## REML criterion at convergence: 1940.1
## 
## Scaled residuals: 
##     Min      1Q  Median      3Q     Max 
## -2.5325 -0.8837 -0.2013  0.9371  1.8528 
## 
## Random effects:
##  Groups   Name        Variance Std.Dev.
##  subject  (Intercept) 0.000    0.000   
##  Batch    (Intercept) 0.000    0.000   
##  Residual             2.481    1.575   
## Number of obs: 515, groups:  subject, 164; Batch, 6
## 
## Fixed effects:
##                           Estimate Std. Error        df t value Pr(>|t|)
## (Intercept)               -0.08096    0.32367 511.00000  -0.250 0.802576
## StrainGMI1000_fcsmut       0.94421    0.44261 511.00000   2.133 0.033378
## StrainGMI1000:DPI          0.29069    0.04444 511.00000   6.542 1.48e-10
## StrainGMI1000_fcsmut:DPI   0.13217    0.03764 511.00000   3.512 0.000485
##                             
## (Intercept)                 
## StrainGMI1000_fcsmut     *  
## StrainGMI1000:DPI        ***
## StrainGMI1000_fcsmut:DPI ***
## ---
## Signif. codes:  0 '***' 0.001 '**' 0.01 '*' 0.05 '.' 0.1 ' ' 1
## 
## Correlation of Fixed Effects:
##             (Intr) StGMI1000_ SGMI1000:
## StrGMI1000_ -0.731                     
## SGMI1000:DP -0.954  0.698              
## SGMI1000_:D  0.000 -0.644      0.000
```

```
###E.g. plot confints
###Make pairwise confints and plot them, with a flipped coordinate system
pairwise_confint <- as.data.frame(confint(glht(disease_lmer, mcp(Strain = "Tukey", interaction_average=T)))$confint)
pairwise_confint$Comparison <- rownames(pairwise_confint)

ggplot(pairwise_confint, aes(x = Comparison, y = Estimate, ymin = lwr, ymax = upr, color = abs(Estimate))) + ###Plot Comparison on x, estimate on y
  scale_x_discrete(limits = rev(levels(as.factor(pairwise_confint$Comparison)))) + ###Rescale x, so the order is inverted 
  geom_errorbar() + geom_point() + ###Draw data
  coord_flip() +  theme(legend.position="none") + xlab("") +###Invert X and Y, hide legend
  ggtitle("Difference in means with 95% confidence interval \ncolored by absolute estimated difference") ##Add a title
```

```
confint_model <- as.data.frame(tidy(confint(disease_lmer)))
```

```
## Computing profile confidence intervals ...
```

```
## Warning in optwrap(optimizer, par = start, fn = function(x)
## dd(mkpar(npar1, : convergence code 3 from bobyqa: bobyqa -- a trust region
## step failed to reduce q
```

```
## Warning in optwrap(optimizer, par = thopt, fn = mkdevfun(rho, 0L), lower
## = fitted@lower): convergence code 3 from bobyqa: bobyqa -- a trust region
## step failed to reduce q

## Warning in optwrap(optimizer, par = thopt, fn = mkdevfun(rho, 0L), lower
## = fitted@lower): convergence code 3 from bobyqa: bobyqa -- a trust region
## step failed to reduce q

## Warning in optwrap(optimizer, par = thopt, fn = mkdevfun(rho, 0L), lower
## = fitted@lower): convergence code 3 from bobyqa: bobyqa -- a trust region
## step failed to reduce q

## Warning in optwrap(optimizer, par = thopt, fn = mkdevfun(rho, 0L), lower
## = fitted@lower): convergence code 3 from bobyqa: bobyqa -- a trust region
## step failed to reduce q

## Warning in optwrap(optimizer, par = thopt, fn = mkdevfun(rho, 0L), lower
## = fitted@lower): convergence code 3 from bobyqa: bobyqa -- a trust region
## step failed to reduce q

## Warning in optwrap(optimizer, par = thopt, fn = mkdevfun(rho, 0L), lower
## = fitted@lower): convergence code 3 from bobyqa: bobyqa -- a trust region
## step failed to reduce q

## Warning in optwrap(optimizer, par = thopt, fn = mkdevfun(rho, 0L), lower
## = fitted@lower): convergence code 3 from bobyqa: bobyqa -- a trust region
## step failed to reduce q

## Warning in optwrap(optimizer, par = thopt, fn = mkdevfun(rho, 0L), lower
## = fitted@lower): convergence code 3 from bobyqa: bobyqa -- a trust region
## step failed to reduce q

## Warning in optwrap(optimizer, par = thopt, fn = mkdevfun(rho, 0L), lower
## = fitted@lower): convergence code 3 from bobyqa: bobyqa -- a trust region
## step failed to reduce q

## Warning in optwrap(optimizer, par = thopt, fn = mkdevfun(rho, 0L), lower
## = fitted@lower): convergence code 3 from bobyqa: bobyqa -- a trust region
## step failed to reduce q

## Warning in optwrap(optimizer, par = thopt, fn = mkdevfun(rho, 0L), lower
## = fitted@lower): convergence code 3 from bobyqa: bobyqa -- a trust region
## step failed to reduce q

## Warning in optwrap(optimizer, par = thopt, fn = mkdevfun(rho, 0L), lower
## = fitted@lower): convergence code 3 from bobyqa: bobyqa -- a trust region
## step failed to reduce q
```

```
confint_slopes <- confint_model[ (1 + 3 + nlevels( di_long$Strain ) ) : ( 3 + 2*nlevels( di_long$Strain ) ) , 2:3 ]

colnames(confint_slopes) <- c("lwr","upr")
confint_slopes$Estimate <- rowMeans(confint_slopes)
confint_slopes$Strain <- levels(di_long$Strain)

###Plot the estimates, below may not be the most straight-foward way to plot this the way I want it, but it works.
ggplot(confint_slopes, aes(x = Strain, y = Estimate, ymin = lwr, ymax = upr, color = abs(Estimate))) + ###Plot Comparison on x, estimate on y
  scale_x_discrete(limits = rev(levels(as.factor(confint_slopes$Strain)))) + ###Rescale x, so the order is inverted 
  geom_errorbar() + geom_point() + ###Draw data
  coord_flip() +  theme(legend.position="none") + xlab("") +###Invert X and Y, hide legend
  ggtitle("Absolute slopes with 95%CI") ##Add a title
```

```
#Intercepts are treatment contrasted
confint_icep <- confint_model[4:( 3 + nlevels( di_long$Strain )),]

confint_icep$Strain <- levels(di_long$Strain)

for (i in 1:nrow(confint_icep)){
  if (i==1){
    confint_icep$Estimate[i] <- mean(c(confint_icep$X2.5..[i],confint_icep$X97.5..[i]))
    confint_icep$upr[i] <- confint_icep$X97.5..[i]
    confint_icep$lwr[i] <- confint_icep$X2.5..[i]
  } else {
    confint_icep$Estimate[i] <- c(confint_icep$Estimate[1]+mean(c(confint_icep$X2.5..[i],confint_icep$X97.5..[i])))
    confint_icep$upr[i] <- c(confint_icep$X97.5..[1]+confint_icep$X97.5..[i])
    confint_icep$lwr[i] <- c(confint_icep$X2.5..[1]+confint_icep$X2.5..[i])
  }
}

ggplot(confint_icep, aes(x = Strain, y = Estimate, ymin = lwr, ymax = upr, color = abs(Estimate))) + ###Plot Comparison on x, estimate on y
  scale_x_discrete(limits = rev(levels(as.factor(confint_icep$Strain)))) + ###Rescale x, so the order is inverted 
  geom_errorbar() + geom_point() + ###Draw data
  coord_flip() +  theme(legend.position="none") + xlab("") +###Invert X and Y, hide legend
  ggtitle("Absolute intercept with 95%CI") ##Add a title
```

Also here, generalized linear hypothesis testing with an adjustment for multiple comparisons is probably relevant.

```
###Test hypothesis that all strains are equal and do compact letter grouping
#Using multcomp glht
summary(glht(disease_lmer, linfct=mcp(Strain="Tukey",interaction_average = T)))
```

```
## 
##   Simultaneous Tests for General Linear Hypotheses
## 
## Multiple Comparisons of Means: Tukey Contrasts
## 
## 
## Fit: lme4::lmer(formula = DI ~ Strain + Strain:DPI + (1 | subject) + 
##     (1 | Batch), data = di_long.useful)
## 
## Linear Hypotheses:
##                               Estimate Std. Error z value Pr(>|z|)  
## GMI1000_fcsmut - GMI1000 == 0   0.9442     0.4426   2.133   0.0329 *
## ---
## Signif. codes:  0 '***' 0.001 '**' 0.01 '*' 0.05 '.' 0.1 ' ' 1
## (Adjusted p values reported -- single-step method)
```

```
cld(glht(disease_lmer, linfct=mcp(Strain="Tukey", interaction_average=T)))
```

```
##        GMI1000 GMI1000_fcsmut 
##            "b"            "a"
```

```
#Using lmerTest lsMeans
lmerlsm <- difflsmeans(disease_lmer)$diffs.lsmeans.table
lmerlsm
```

```
##                                 Estimate Standard Error  DF t-value
## Strain GMI1000 - GMI1000_fcsmut   0.2055           0.14 511    1.47
##                                 Lower CI Upper CI p-value
## Strain GMI1000 - GMI1000_fcsmut  -0.0696   0.4806  0.1428
```

```
Comparison = str_split_fixed(rownames(lmerlsm),"Strain ",2)[,2]

### Produce compact letter display
#Errors as there are no significant differences.
# cldList(comparison = Comparison,
#         p.value    = p.adjust(lmerlsm$'p-value',
#                        method =  "bonferroni")   ,
#         threshold = 0.05)
```

Using the linear model and raw data, different displays can be plotted, for example, a boxplot of the “Useful” data-points combined with the predictions of the linear model.

```
##Add predictions to full dataset
library("modelr")
```

```
## 
## Attaching package: 'modelr'
```

```
## The following object is masked from 'package:broom':
## 
##     bootstrap
```

```
##Add predictions to full dataset
di_long <- add_predictions(di_long,disease_lmer,var="lmer.pred")
```

```
## Warning: contrasts dropped from factor Strain
```

```
###
ggplot(data=di_long, aes(x=DPI,y=DI))+
  geom_boxplot(aes(color=Strain,group=DPI),data=filter(di_long, Useful=="Yes"))+
  geom_smooth(aes(y=lmer.pred,color=Strain), method="lm", alpha=0.6) +
  facet_wrap(~Strain)+
  ggtitle("Boxplots and linear fit for individual strains")
```

Finally, of the methods discussed here, those relying on a linear model of raw observations over time, can be used for other kinds of observations, e.g. bacterial titers.

Plot residuals of the lmm to assess if this appears normal (random)

```
di_long$resid[di_long$Useful=="Yes"] <- resid(disease_lmer)
ggplot(di_long[di_long$Useful=="Yes",], aes(x=DPI,y=resid)) + geom_jitter(aes(color=Strain))
```

# Survival Analysis

Note, that in this analysis, we are looking at symptom onset. As soon as a subject reaches a disease index of 1, it is considered symptomatic. The actual survival analysis is done the same way as before, but the generation of the survival table is performed using a different cutoff.

## Generation of a survival table

```
###Set the cutoff by assigning a number to the variable cutoff
cutoff <- c(1)
```

All of the observations that are above the dotted line in the plot below are *dead*, at the day they cross that line. Those that never cross the line are alive until the end of observations, and are “right censored”, meaning that their event was not observed during the time this subject was observed.

```
ggplot(data=di_long) +
                  geom_jitter(aes(x=DPI, y=DI,color=Strain,shape=Batch)) +
                  geom_segment(aes(x=0, xend=max(DPI)+0.5,y=cutoff, yend=cutoff), linetype="dotted") +
                  labs(x = "Days post infection", y = "Disease indices", title="Scatterplot of disease indices\n cutoff plotted as dotted line") +
                  coord_cartesian(xlim=c(-0.1,10))
```

A “survival table” can be generated using the following code. This code works on the long table, generated in the beginning, and the cutoff variable defined above.

*Changed* Generation of the survival table. As the wide table used for the sim\_data.csv is not available here, the variables are regenerated from the subject identifiers.

```
###Generate survival table
###str_split_fixed does use a regex, dot needs to be escaped
library("stringr")

surv_from_DI <- data.frame(Subject=levels(di_long$subject),
                         Strain=str_split_fixed(levels(di_long$subject), "\\.",3)[,1],
                         Plant=str_split_fixed(levels(di_long$subject), "\\.",3)[,2],
                         Batch=str_split_fixed(levels(di_long$subject), "\\.",3)[,3])

###Fill survival table based on the di_long table. This generates warnings. These can be ignored and come from the min()
for (i in 1:max(as.numeric(di_long$subject))) { #Go by subject
  dummy <- di_long[as.numeric(di_long$subject)==i,] #generate dummy for the subject
  if (is.infinite(min(dummy$DPI[which(dummy$DI >= cutoff)]))) { #If none of the DI is greater than the cutoff (this is where warnings are generated, min on an empty object returns infinite and a warning!)
    surv_from_DI$End[i] <- max(dummy$DPI) #Generate a  observation, censoring at the maximum DPI recorded
    surv_from_DI$Death[i] <- 0 #Still alive, because it did not pass the cutoff
  } else { #If more than zero DI are greater than the cutoff
    surv_from_DI$End[i] <- min(dummy$DPI[which(dummy$DI >= cutoff)]) #Use the lowest DPI where condition is met
    surv_from_DI$Death[i] <- 1 #record as dead
  }
}
rm(dummy)
```

## Kaplan-Meier estimates of survival

Kaplan-Meier estimates of survival are the basic tool of survival analysis. These can be estimated using the survfit function from the “survival” package.c

```
library("survival")
```

```
###Ignore batch information, as it was ignored in the MPMI publication
surv_DI_fit <- survfit(Surv(End, Death) ~Strain, data=surv_from_DI)
```

The survminer package provides the ggsurvplot() function. This works nicely on datasets with few treatments. However, for larger datasets, I think it is easier to initially generate a data frame that contains the whole fit and plot with “normal” ggplot2

```
###Strata dummy generation, modified from kmiddleton / rexamples 
#Function to make KM dataframe 
survfit_to_df <- function(x) {
  oldw <- getOption("warn") #Supress warnings
  options(warn = -1)
  
  strata_dummy <-NULL
  for(i in 1:length(x$strata)){
    # add vector for one strata according to number of rows of strata
    strata_dummy <- c(strata_dummy, rep(names(x$strata)[i], x$strata[i]))
  }
  #make x.df from x..
  x.df <- data.frame( 
    time = x$time, 
    n.risk = x$n.risk, 
    n.event = x$n.event, 
    surv = x$surv, 
    strata = strata_dummy, 
    upper = x$upper, 
    lower = x$lower 
  ) 
  zeros <- data.frame(time = 0, surv = 1, strata = names((x$strata)), 
                      upper = 1, lower = 1)
  
  x.df <- plyr::rbind.fill(zeros, x.df) 
  rm(strata_dummy)
  rm(zeros)
  return(x.df)
  options(warn = oldw)
}

surv_DI_fit.df <- survfit_to_df(surv_DI_fit)
###Some stuff to rename other stuff, this needs to be adapted if other variables are used.

surv_DI_fit.df$Strain <- as.factor( 
  str_split_fixed(
    matrix( 
      nrow=length(surv_DI_fit.df$strata),ncol=1, unlist(strsplit(as.character(surv_DI_fit.df$strata),", ")), byrow=T )[,1]
    ,"=",2)[,2])

###End of data frame generation
###Start plotting
ggplot(surv_DI_fit.df, aes(time, surv, colour = Strain)) + 
  geom_step(aes(y = surv*100)) +
  facet_wrap(~Strain) +
  ggtitle("Survival estimates")
```

## Analysis of survival curves and fits

Comparing the Kaplan-Meier survival estimates can be done in different ways.

### Logrank testing

The below produces all pairwise comparisons of the Kaplan Meier estimate of survival using a logrank test.

```
###Make a table of pairwise chisq pvalues, for the logrank test.
#Based on a post to the R Mailing list by T. Therneau
pw_logrank_test_type <- 0 ###0 for logrank, 1 for peto and peto
pw_logrank <- matrix(0., nlevels(surv_from_DI$Strain),nlevels(surv_from_DI$Strain))
for (i in 1:nlevels(surv_from_DI$Strain)) {
  for (j in (1:nlevels(surv_from_DI$Strain))[-i]) {
    datasubset <- droplevels(subset( surv_from_DI,
      surv_from_DI$Strain %in% (unique(surv_from_DI$Strain))[c(i,j)]))
    temp <- survdiff(Surv(End, Death)~Strain+strata(Batch), data=datasubset, rho=pw_logrank_test_type)
      
    pw_logrank[i,j] <- pchisq(temp$chisq, df=1, lower=F) ##df will always be 1 because this is pairwise
    }
}
colnames(pw_logrank) <- levels(surv_from_DI$Strain)
rownames(pw_logrank) <- levels(surv_from_DI$Strain)
#Make dummy adjustment table
pw_logrank_adjBon <- pw_logrank
#Fill adjusted pvalue table.
for (i in 1:ncol(pw_logrank)) {
  pw_logrank_adjBon[,i] <- cbind(p.adjust(pw_logrank[,i], method="bonferroni"))
}
```

```
stargazer::stargazer(pw_logrank_adjBon,type="html",title="Pairwise Chisq p-values (Bonferroni adjusted)")
```

**Pairwise Chisq p-values (Bonferroni adjusted)**

|  | | |
|  | GMI1000 | GMI1000\_fcsmut |
|  | | |
| GMI1000 | 0 | 0.017 |
| GMI1000\_fcsmut | 0.017 | 0 |
|  | | |

```
library("survcomp")
library("rms")
```

### Regressions

Generally a survival regression does not assume proportionality of hazards. A survival regression is fit to a distribution, defined by dist=“”.

```
####Survival Regression###
###This is done using functions from rms.
###psm is a survival::survreg wrapper. but the output is more handle-able.
library("modelr")
ddist <- datadist(surv_from_DI)
options(datadist="ddist")
psurv_gaus <- psm(Surv(End, Death) ~Strain, data=surv_from_DI, dist="gaussian")
psurv_logistic <- psm(Surv(End, Death) ~Strain, data=surv_from_DI, dist="logistic")
psurv_lnorm <- psm(Surv(End, Death) ~Strain, data=surv_from_DI, dist="lognormal")
psurv_wei <- psm(Surv(End, Death) ~Strain, data=surv_from_DI, dist="weibull")

###Same with survreg()
s_reg_gaus <- survreg(Surv(End, Death) ~Strain, data=surv_from_DI, dist="gaussian")
s_reg_logistic <- survreg(Surv(End, Death) ~Strain, data=surv_from_DI, dist="logistic")
s_reg_lnorm <- survreg(Surv(End, Death) ~Strain, data=surv_from_DI, dist="lognormal")
s_reg_wei <- survreg(Surv(End, Death) ~Strain, data=surv_from_DI, dist="weibull")  

aic.scores.psurv <- rbind(
  extractAIC(psurv_wei),
  extractAIC(psurv_gaus),
  extractAIC(psurv_logistic),
  extractAIC(psurv_lnorm))
###Make useable AIC table
rownames(aic.scores.psurv) <- c("Weibull", "Gaussian", "Logist", "Lognorm")
colnames(aic.scores.psurv) <- c("df", "AIC")
###Call table
```

```
stargazer::stargazer(aic.scores.psurv,type="html",title="AIC Scores")
```

**AIC Scores**

|  | | |
|  | df | AIC |
|  | | |
| Weibull | 3 | 747.821 |
| Gaussian | 3 | 753.646 |
| Logist | 3 | 746.442 |
| Lognorm | 3 | 708.112 |
|  | | |

From the table above, the model with the lowest AIC score can be chosen. For this analysis, this is the lognormal model, but this does not have to apply to other experiments. Then, one can inspect that model for significance

```
summary(glht(psurv_lnorm,linfct=mcp(Strain="Tukey")))
```

```
## 
##   Simultaneous Tests for General Linear Hypotheses
## 
## Multiple Comparisons of Means: Tukey Contrasts
## 
## 
## Fit: psm(formula = Surv(End, Death) ~ Strain, data = surv_from_DI, 
##     dist = "lognormal")
## 
## Linear Hypotheses:
##                               Estimate Std. Error z value Pr(>|z|)  
## GMI1000_fcsmut - GMI1000 == 0  0.10127    0.05151   1.966   0.0493 *
## ---
## Signif. codes:  0 '***' 0.001 '**' 0.01 '*' 0.05 '.' 0.1 ' ' 1
## (Adjusted p values reported -- single-step method)
```

Again, one can then inspect the differences in the model, for example using pairwise comparisons of means.

```
pairwise_confint_sreg <- as.data.frame(confint(glht(psurv_lnorm, mcp(Strain = "Tukey")))$confint)
pairwise_confint_sreg$Comparison <- rownames(pairwise_confint_sreg)
###Plot the comparisons, below may not be the most straight-foward way to plot this the way I want it, but it works.
ggplot(pairwise_confint_sreg, aes(x = Comparison, y = Estimate, ymin = lwr, ymax = upr, color = Comparison)) + ###Plot Comparison on x, estimate on y
  scale_x_discrete(limits = rev(levels(as.factor(pairwise_confint$Comparison)))) + ###Rescale x, so the order is inverted 
  geom_errorbar() + geom_point() + ###Draw data
  coord_flip() +  theme(legend.position="none") + xlab("") +###Invert X and Y, hide legend
  ggtitle("Difference in means with 95% Confidence interval") ##Add a title
```

### Plotting of parametric survival regression

It is possible, but not really easy, to plot the generated curves. These curves are the result of fitting the data to a distribution in the earlier section. Doing this in a manner that is compatible with ggplot2 is not straightforward. Below is code to generate plots of the KM estimates per batch and the generated regression. This is performed for the four distributions above, and can be adapted to different distributions if necessary.

```
library("tidyr")
```

```
## 
## Attaching package: 'tidyr'
```

```
## The following object is masked from 'package:Matrix':
## 
##     expand
```

```
###Step 1, extract the coefficients. These are relative to Strain1 because Strain is treatment contrasted.

for (i in 1:nlevels(surv_DI_fit.df$Strain)) { #For loop through strains
  if(i==1) { #Strain1 is relative to itself, so no change
  coef_wei <- list()
  coef_logistic <- list()
  coef_gaus <- list()
  coef_lnorm <- list()
  coef_wei[i] <- coef(s_reg_wei)[i]
  coef_logistic[i] <- coef(s_reg_logistic)[i]
  coef_gaus[i] <- coef(s_reg_gaus)[i]
  coef_lnorm[i] <- coef(s_reg_lnorm)[i]
  } else { ###Other strains are relative to 1
  coef_wei[i] <- coef(s_reg_wei)[1] + coef(s_reg_wei)[i]
  coef_logistic[i] <- coef(s_reg_logistic)[1] + coef(s_reg_logistic)[i]
  coef_gaus[i] <- coef(s_reg_gaus)[1] + coef(s_reg_gaus)[i]
  coef_lnorm[i] <- coef(s_reg_lnorm)[1] + coef(s_reg_lnorm)[i]
  }
}
##Step 2
####Store the coefficients and the scale in a new data frame, of parameters
### Keep in mind that survreg.distributions$weibull is different from rweibull, hence the difference in names.
sregparams <- data.frame(
  Strain = rep(levels(surv_from_DI$Strain),4 ), #Fill with strains
  scale.wei = exp(unlist(coef_wei)), #weibull fit scale parameters
  scale.logistic = rep(s_reg_logistic$scale, nlevels(surv_from_DI$Strain)), #fill with logis scales
  scale.gaus = rep(s_reg_gaus$scale, nlevels(surv_from_DI$Strain)), #fill with gaus scales
  scale.lnorm = rep(s_reg_lnorm$scale, nlevels(surv_from_DI$Strain)), #fill with lnorm scale
  shape.wei =  rep(1/s_reg_wei$scale, nlevels(surv_from_DI$Strain)), #shape for weibull
  shape.logistic = unlist(coef_logistic), #shape for logistic
  shape.gaus =  unlist(coef_gaus), #shape for gaus
  shape.lnorm =   unlist(coef_lnorm) #shape for lnorm
  )
##Step 3
###Calculate the "daily" value of each curve
for (i in 1:nlevels(surv_DI_fit.df$Strain)){
  if(i==1) {
    wei <- list()
    logis <- list()
    gaus <- list()
    lnorm <- list()
  }
  x <- levels(surv_DI_fit.df$Strain)[i]
  n <- c(1:max(surv_from_DI$End))
  data <- filter(sregparams, Strain==x)
  time <- n
  wei <- cbind(wei, pweibull(
    q=n,
    scale=data$scale.wei,
    shape=data$shape.wei,
    lower.tail=FALSE))
  logis <- cbind(logis,plogis(
    q=n,
    scale=data$scale.logistic,
    location=data$shape.logistic,
    lower.tail=FALSE  ))
  gaus <- cbind(gaus,pnorm(
   q=n,
   sd=data$scale.gaus,
   mean=data$shape.gaus,
   lower.tail = F))
  lnorm <- cbind(lnorm, plnorm(
    q=n,
    sd=data$scale.lnorm,
    mean=data$shape.lnorm,
    lower.tail=F))
}

##Step 4
###Put all the curves into a data.frame that contains information on "time" and also "Strain", for compatibility with other data.frames
sreg_curves <- data.frame(
  wei.sreg = cbind(unlist(wei)),
  logis.sreg = cbind(unlist(logis)),
  gaus.sreg = cbind(unlist(gaus)),
  lnorm.sreg = cbind(unlist(lnorm)),
  Strain = rep(unlist(levels(surv_DI_fit.df$Strain)),each=max(surv_from_DI$End)),
  time = rep(c(1:max(surv_from_DI$End)), nlevels(surv_DI_fit.df$Strain))
)
##Step 5
###Turn that data.frame into a long data.frame (not used here but for other figures.)
sreg_long <- sreg_curves %>% gather(., key="Distribution",values = c(lnorm.sreg, wei.sreg,gaus.sreg,logis.sreg) )
sreg_long$Distribution <- as.factor(sreg_long$Distribution)
##Levels: gaus.sreg lnorm.sreg logis.sreg wei.sreg
levels(sreg_long$Distribution) <- c("Gaussian","Lognormal","Loglogistic","Weibull")
```

Now, these can be plotted and inspected visually..

```
###Plot of KM+Weibull
  ggplot(surv_DI_fit.df, aes(time, surv, colour = Strain)) +
  geom_step() +
  geom_line(data=sreg_curves,aes(y=wei.sreg),color="black") +
  facet_wrap(~Strain) + 
  ggtitle("Kaplan-Meier estimates and fit to\nWeibull distribution")
```

```
###Plot of KM+Logistic
  ggplot(surv_DI_fit.df, aes(time, surv, colour = Strain)) +
    geom_step() +
    geom_line(data=sreg_curves,aes(y=logis.sreg),color="black") +
    facet_wrap(~Strain) + 
    ggtitle("Kaplan-Meier estimates and fit to\n Logistic distribution")
```

```
###Plot of KM+Gaussian  
  ggplot(surv_DI_fit.df, aes(time, surv, colour = Strain)) +
    geom_step() +
    geom_line(data=sreg_curves,aes(y=gaus.sreg),color="black") +
    facet_wrap(~Strain) + 
    ggtitle("Kaplan-Meier estimates and fit to\nGaussian distribution")
```

```
###Plot of KM+Lognormal  
  ggplot(surv_DI_fit.df, aes(time, surv, colour = Strain)) +
    geom_step() +
    geom_line(data=sreg_curves,aes(y=lnorm.sreg),color="black") +
    facet_wrap(~Strain) + 
    ggtitle("Kaplan-Meier estimates and fit to\nLognormal distribution")
```

## Cox-Proportional hazards and hazard ratios

Different approaches to survival analysis, are based on analysing the hazards. The hazard is the probability of experiencing an event at a given timepoint. Many hazard based analysis assume that hazards are proportional between treatments, meaning that their relationship can be described by a constant.

```
###Cox-Proportional hazards####
#Build model
srv_coxph <- coxph(Surv(End, Death) ~Strain + strata(Batch), data=surv_from_DI) 
###Check porportionality of hazards
cox.zph(srv_coxph, transform = "log")
```

```
##                          rho chisq     p
## StrainGMI1000_fcsmut -0.0729 0.853 0.356
```

Here the hazards are proportional, and can be analyzed using hazard ratios.

```
####Hazard ratio
haz_rats <- hazard.ratio(x= surv_from_DI$Strain, 
                         surv.time = surv_from_DI$End, 
                         surv.event = surv_from_DI$Death, 
                         strat = surv_from_DI$Batch, 
                         method.test = "wald" ) ###Overall hazard ratios

###Pairwise hazard ratios / modified from the pairwise chisq calculation
pw_hazrats <- matrix(0., nlevels(surv_from_DI$Strain),nlevels(surv_from_DI$Strain))
for (i in 1:nlevels(surv_from_DI$Strain)) {
  for (j in (1:nlevels(surv_from_DI$Strain))[-i]) {
    datasubset <- droplevels(subset( surv_from_DI,
      surv_from_DI$Strain %in% (unique(surv_from_DI$Strain))[c(i,j)]))
    temp <- hazard.ratio(
      x= datasubset$Strain, 
      surv.time = datasubset$End, 
      surv.event = datasubset$Death, 
      strat = datasubset$Batch, 
      method.test = "likelihood.ratio" ###Define test to determine p.
      )
    pw_hazrats[i,j] <- temp$p.value
    }
}
colnames(pw_hazrats) <- levels(surv_from_DI$Strain)
rownames(pw_hazrats) <- levels(surv_from_DI$Strain)
```

```
stargazer::stargazer(pw_hazrats,type="html",title="Pairwise hazard ratio pvalues")
```

**Pairwise hazard ratio pvalues**

|  | | |
|  | GMI1000 | GMI1000\_fcsmut |
|  | | |
| GMI1000 | 0 | 0.008 |
| GMI1000\_fcsmut | 0.008 | 0 |
|  | | |

If the hazards are found to be non-proportional, it might be a good idea to perform survival regression analysis, or pairwise log-rank testing (see earlier) instead of hazard ratio tests. Alternatives may also come from the use of Cox mixed-effects model, similar to linear mixed-effects model, but with a different type of response variable.

```
library("coxme")
```

```
## Loading required package: bdsmatrix
```

```
## 
## Attaching package: 'bdsmatrix'
```

```
## The following object is masked from 'package:SparseM':
## 
##     backsolve
```

```
## The following object is masked from 'package:base':
## 
##     backsolve
```

```
cme <-  coxme(Surv(End, Death) ~Strain + (1|Batch), data=surv_from_DI)
anova(cme)
```

```
## Analysis of Deviance Table
##  Cox model: response is Surv(End, Death)
## Terms added sequentially (first to last)
## 
##         loglik Chisq Df Pr(>|Chi|)  
## NULL   -663.05                      
## Strain -659.91 6.278  1    0.01222 *
## ---
## Signif. codes:  0 '***' 0.001 '**' 0.01 '*' 0.05 '.' 0.1 ' ' 1
```

```
summary(glht(cme,linfct=mcp(Strain="Tukey")))
```

```
## 
##   Simultaneous Tests for General Linear Hypotheses
## 
## Multiple Comparisons of Means: Tukey Contrasts
## 
## 
## Fit: coxme(formula = Surv(End, Death) ~ Strain + (1 | Batch), data = surv_from_DI)
## 
## Linear Hypotheses:
##                               Estimate Std. Error z value Pr(>|z|)  
## GMI1000_fcsmut - GMI1000 == 0  -0.4004     0.1644  -2.435   0.0149 *
## ---
## Signif. codes:  0 '***' 0.001 '**' 0.01 '*' 0.05 '.' 0.1 ' ' 1
## (Adjusted p values reported -- single-step method)
```

# Comparison of analyis method results.

An inherent question when analyzing data, is which analysis produced which result and why. Below, the outputs from the three major analysis performed are printed, so they can be compared.

```
summary(auc_lmer)
```

```
## Linear mixed model fit by REML t-tests use Satterthwaite approximations
##   to degrees of freedom [lmerMod]
## Formula: AUC ~ Strain + (1 | Batch)
##    Data: auc_df
## 
## REML criterion at convergence: 1220.9
## 
## Scaled residuals: 
##     Min      1Q  Median      3Q     Max 
## -3.0237 -0.6276  0.1797  0.7031  1.6521 
## 
## Random effects:
##  Groups   Name        Variance Std.Dev.
##  Batch    (Intercept) 30.33    5.507   
##  Residual             97.05    9.851   
## Number of obs: 164, groups:  Batch, 6
## 
## Fixed effects:
##                      Estimate Std. Error      df t value Pr(>|t|)    
## (Intercept)            25.201      2.502   5.900  10.071 6.17e-05 ***
## StrainGMI1000_fcsmut   -2.866      1.539 156.840  -1.863   0.0644 .  
## ---
## Signif. codes:  0 '***' 0.001 '**' 0.01 '*' 0.05 '.' 0.1 ' ' 1
## 
## Correlation of Fixed Effects:
##             (Intr)
## StrGMI1000_ -0.307
```

```
summary(disease_lmer)
```

```
## Linear mixed model fit by REML t-tests use Satterthwaite approximations
##   to degrees of freedom [lmerMod]
## Formula: DI ~ Strain + Strain:DPI + (1 | subject) + (1 | Batch)
##    Data: di_long.useful
## 
## REML criterion at convergence: 1940.1
## 
## Scaled residuals: 
##     Min      1Q  Median      3Q     Max 
## -2.5325 -0.8837 -0.2013  0.9371  1.8528 
## 
## Random effects:
##  Groups   Name        Variance Std.Dev.
##  subject  (Intercept) 0.000    0.000   
##  Batch    (Intercept) 0.000    0.000   
##  Residual             2.481    1.575   
## Number of obs: 515, groups:  subject, 164; Batch, 6
## 
## Fixed effects:
##                           Estimate Std. Error        df t value Pr(>|t|)
## (Intercept)               -0.08096    0.32367 511.00000  -0.250 0.802576
## StrainGMI1000_fcsmut       0.94421    0.44261 511.00000   2.133 0.033378
## StrainGMI1000:DPI          0.29069    0.04444 511.00000   6.542 1.48e-10
## StrainGMI1000_fcsmut:DPI   0.13217    0.03764 511.00000   3.512 0.000485
##                             
## (Intercept)                 
## StrainGMI1000_fcsmut     *  
## StrainGMI1000:DPI        ***
## StrainGMI1000_fcsmut:DPI ***
## ---
## Signif. codes:  0 '***' 0.001 '**' 0.01 '*' 0.05 '.' 0.1 ' ' 1
## 
## Correlation of Fixed Effects:
##             (Intr) StGMI1000_ SGMI1000:
## StrGMI1000_ -0.731                     
## SGMI1000:DP -0.954  0.698              
## SGMI1000_:D  0.000 -0.644      0.000
```

```
summary(psurv_lnorm)
```

```
##              Effects              Response : Surv(End, Death) 
## 
##  Factor                          Low High Diff. Effect  S.E.    
##  Strain - GMI1000_fcsmut:GMI1000 1   2    NA    0.10127 0.051506
##   Survival Time Ratio            1   2    NA    1.10660       NA
##  Lower 0.95  Upper 0.95
##  -0.00044455 0.20299   
##   0.99956000 1.22510
```

An easier comparisons might be accomplished with the compact letter display.

```
cld(glht(auc_lmer, linfct=mcp(Strain="Tukey")))
```

```
##        GMI1000 GMI1000_fcsmut 
##            "a"            "a"
```

```
#Compact letters for lmerTest objects are a little tricky. This solution comes from the rcompanion.
### Extract lsmeans table

lmerlsm <- difflsmeans(disease_lmer)$diffs.lsmeans.table

Comparison = str_split_fixed(rownames(lmerlsm),"Strain ",2)[,2]

### Produce compact letter display

library(rcompanion)

#cldList(comparison = Comparison,
        #p.value    = p.adjust(lmerlsm$'p-value',
       #                method =  "bonferroni")   ,
        #threshold = 0.05)


cld(glht(srv_coxph,linfct=mcp(Strain="Tukey")))
```

```
##        GMI1000 GMI1000_fcsmut 
##            "a"            "b"
```

```
cld(glht(psurv_lnorm,linfct=mcp(Strain="Tukey")))
```

```
##        GMI1000 GMI1000_fcsmut 
##            "a"            "b"
```

```
cld(glht(cme,linfct=mcp(Strain="Tukey")))
```

```
##        GMI1000 GMI1000_fcsmut 
##            "a"            "b"
```

# Session Info

```
sessionInfo()
```

```
## R version 3.3.3 (2017-03-06)
## Platform: x86_64-w64-mingw32/x64 (64-bit)
## Running under: Windows 10 x64 (build 14393)
## 
## locale:
## [1] LC_COLLATE=English_United States.1252 
## [2] LC_CTYPE=English_United States.1252   
## [3] LC_MONETARY=English_United States.1252
## [4] LC_NUMERIC=C                          
## [5] LC_TIME=English_United States.1252    
## 
## attached base packages:
## [1] stats     graphics  grDevices utils     datasets  methods   base     
## 
## other attached packages:
##  [1] coxme_2.2-5      bdsmatrix_1.3-2  tidyr_0.6.1      rms_5.1-0       
##  [5] SparseM_1.74     Hmisc_4.0-2      Formula_1.2-1    lattice_0.20-34 
##  [9] survcomp_1.24.0  prodlim_1.5.9    modelr_0.1.0     stringr_1.2.0   
## [13] rcompanion_1.5.0 multcomp_1.4-6   TH.data_1.0-8    MASS_7.3-45     
## [17] survival_2.40-1  mvtnorm_1.0-5    broom_0.4.2      lmerTest_2.0-33 
## [21] lme4_1.1-12      Matrix_1.2-8     MESS_0.4-3       geepack_1.2-1   
## [25] ggplot2_2.2.1    dplyr_0.5.0     
## 
## loaded via a namespace (and not attached):
##  [1] survivalROC_1.0.3    nlme_3.1-131         pbkrtest_0.4-7      
##  [4] ordinal_2015.6-28    RColorBrewer_1.1-2   rprojroot_1.2       
##  [7] tools_3.3.3          backports_1.0.5      R6_2.2.0            
## [10] KernSmooth_2.23-15   rpart_4.1-10         rmeta_2.16          
## [13] nortest_1.0-4        DBI_0.5-1            lazyeval_0.2.0      
## [16] mgcv_1.8-17          colorspace_1.3-2     ade4_1.7-5          
## [19] nnet_7.3-12          gridExtra_2.2.1      mnormt_1.5-5        
## [22] quantreg_5.29        htmlTable_1.9        hermite_1.1.1       
## [25] expm_0.999-1         sandwich_2.3-4       labeling_0.3        
## [28] scales_0.4.1         checkmate_1.8.2      polspline_1.1.12    
## [31] lmtest_0.9-35        psych_1.6.12         mc2d_0.1-18         
## [34] multcompView_0.1-7   digest_0.6.12        foreign_0.8-67      
## [37] minqa_1.2.4          rmarkdown_1.3        base64enc_0.1-3     
## [40] WRS2_0.9-1           htmltools_0.3.5      manipulate_1.0.1    
## [43] htmlwidgets_0.8      SuppDists_1.1-9.4    zoo_1.7-14          
## [46] acepack_1.4.1        car_2.1-4            magrittr_1.5        
## [49] modeltools_0.2-21    Rcpp_0.12.9          DescTools_0.99.19   
## [52] munsell_0.4.3        ucminf_1.1-4         stringi_1.1.2       
## [55] yaml_2.1.14          plyr_1.8.4           grid_3.3.3          
## [58] parallel_3.3.3       stargazer_5.2        splines_3.3.3       
## [61] knitr_1.15.1         EMT_1.1              boot_1.3-18         
## [64] reshape2_1.4.2       codetools_0.2-15     stats4_3.3.3        
## [67] evaluate_0.10        latticeExtra_0.6-28  data.table_1.10.4   
## [70] nloptr_1.0.4         bootstrap_2017.2     miscTools_0.6-22    
## [73] MatrixModels_0.4-1   gtable_0.2.0         purrr_0.2.2         
## [76] reshape_0.8.6        assertthat_0.1       coin_1.1-3          
## [79] BSDA_1.01            tibble_1.2           lava_1.4.7          
## [82] cluster_2.0.5        maxLik_1.3-4         RVAideMemoire_0.9-63
```
