# Supplementary material for: A Practical Guide to Visualization and Statistical Analysis of R. solanacearum Infection Data Using R
Source: Front Plant Sci. 2017 Apr 24;8:623. doi: 10.3389/fpls.2017.00623 (PMC5401893; doi:10.3389/fpls.2017.00623)
Supplement: Supplementary file 4 [file Data_Sheet_4.ZIP › S4_Ravelomanantsoa_Prior.html]

Approaches to analyze experimental R. solanacearum infections: Supplementary Material 4: Datasets from the Prior Lab Survival Data


# Approaches to analyze experimental *R. solanacearum* infections: *Supplementary Material 4:* Datasets from the Prior Lab Survival Data

#### *Niklas Schandry*

# General information on this file

This document is part of the supplementary material of “A practical guide to descriptive and statistical analysis of R. solanacearum infection data using R”. This file explores more complex survival analysis, that contain multiple levels of Plants (genotype) and Strains. This unpublished work by Santatra Ravelomanantsoa and Philippe Prior, both with CIRAD / INRA UMR PVBMT (Réunion Island), has been pseudonymized.

# Data Import

## Specifying the data

Initially, data needs to be read into R.

```
###Name of the file to be read
table <- c("S4_Ravelomanantsoa_Prior.csv")
```

## Reading data and formatting

```
disease_index <- as.data.frame(read.table(table, header=T,
                                          sep=";" , ###Sets the seperator of the csv file
                                          dec=","), ###Sets the decimal operatore of the csv file
                                          stringsAsFactors=T) ###
```

Using str() one can see if the table was properly imported.

```
str(disease_index)
```

```
## 'data.frame':    3920 obs. of  12 variables:
##  $ Strain  : Factor w/ 14 levels "RS1","RS10","RS11",..: 1 1 1 1 1 1 1 1 1 1 ...
##  $ Plant   : Factor w/ 8 levels "A","B","C","D",..: 1 1 1 1 1 1 1 2 2 2 ...
##  $ X1      : int  0 0 0 0 0 0 0 0 0 0 ...
##  $ X8      : int  0 0 0 0 0 0 0 0 0 0 ...
##  $ X11     : int  0 0 0 0 0 0 0 0 0 0 ...
##  $ X15     : int  0 0 0 0 0 0 0 0 0 0 ...
##  $ X18     : int  0 0 0 0 0 0 0 0 0 0 ...
##  $ X22     : int  0 0 0 0 0 0 0 0 0 0 ...
##  $ X25     : int  0 0 0 1 1 0 1 0 1 1 ...
##  $ X32     : int  0 0 0 1 1 0 1 0 1 1 ...
##  $ X43     : Factor w/ 3 levels "0","1","na": 1 1 1 2 2 1 2 1 2 2 ...
##  $ ROTOPLAN: int  1 1 1 1 1 1 1 1 1 1 ...
```

This table is a survival table already, go directly into surv\_table.

```
library("tidyr")
```

```
surv_table<- gather(disease_index, key=DPI, 
                     value=Diseased,
                     c(X1,X8,X11,X15,X18,X22,X25,X32, X43),
                     na.rm=T)
```

```
## Warning: attributes are not identical across measure variables; they will
## be dropped
```

```
str(surv_table)
```

```
## 'data.frame':    34186 obs. of  5 variables:
##  $ Strain  : Factor w/ 14 levels "RS1","RS10","RS11",..: 1 1 1 1 1 1 1 1 1 1 ...
##  $ Plant   : Factor w/ 8 levels "A","B","C","D",..: 1 1 1 1 1 1 1 2 2 2 ...
##  $ ROTOPLAN: int  1 1 1 1 1 1 1 1 1 1 ...
##  $ DPI     : chr  "X1" "X1" "X1" "X1" ...
##  $ Diseased: chr  "0" "0" "0" "0" ...
```

```
###Do stuff to surv_table that makes it more useable
surv_table$DPI <- as.numeric(
  na.omit(
    as.numeric(
      unlist(
        strsplit(
          as.character( 
            surv_table$DPI
          ),
          "X" )
      )
    )
  )
)
surv_table <- surv_table[(surv_table$Diseased %in% c(0,1)),] ##Drop Disease recordings that are not binary
surv_table$Strain <- as.factor(surv_table$Strain)
surv_table$Plant <- as.factor(surv_table$Plant)
surv_table$ROTOPLAN <- as.factor(surv_table$ROTOPLAN)
surv_table$StrainPlant <- interaction(surv_table$Strain,surv_table$Plant)
surv_table$StrainRoto <- interaction(surv_table$Strain, surv_table$ROTOPLAN)
surv_table$PlantRoto <- interaction(surv_table$Plant, surv_table$ROTOPLAN)
contrasts(surv_table$Strain) <- "contr.treatment"
contrasts(surv_table$Plant) <- "contr.treatment"
contrasts(surv_table$ROTOPLAN) <- "contr.sum"
contrasts(surv_table$StrainPlant) <- "contr.treatment"
```

In this dataset, Strain is the bacterial strain, Plant is the plant genotype that was infected, and ROTOPLAN, is the rotoplan (a kind of greenhouse) that this infection was carried out in. In other words, ROTOPLAN is an indicator that descriminates replicates.

Above, i am generating interaction columns. In the R notation interactions are denoted using \* in a formula. Strain\*Plant in a formula, is equivalent to interaction(Strain,Plant). For me, predifining the interaction terms, makes it easier to tangle them apart for visualization.I also find it easier to interpret.

# Survival Analysis

Initially, one may enjoy generating Kaplan-Meier estimates of the survival data. This is done using the survfit function. Here, the rotoplan is not included as a predictor.

```
library("survival")
surv_fit <- survfit(Surv(DPI, as.numeric(Diseased), type="right") ~Strain + Plant, data=surv_table)

###Make Dataframe for plotting
strata_dummy <-NULL
for(i in 1:length(surv_fit$strata)){
      # add vector for one strata according to number of rows of strata
      strata_dummy <- c(strata_dummy, rep(names(surv_fit$strata)[i], surv_fit$strata[i]))
}
###Data frame generation inspired by a post by Hadley Wickham to the ggplot2 Googlegroup
surv_fit.df <- data.frame( 
  time = surv_fit$time, 
  n.risk = surv_fit$n.risk, 
  n.event = surv_fit$n.event, 
  surv = surv_fit$surv, 
  strata = strata_dummy, 
  upper = surv_fit$upper, 
  lower = surv_fit$lower 
) 
zeros <- data.frame(time = 0, surv = 1, strata = names((surv_fit$strata)), 
  upper = 1, lower = 1)

surv_fit.df <- plyr::rbind.fill(zeros, surv_fit.df) ###I dont want to load plyr because i guess it will interfere with dplyr...
rm(strata_dummy)
rm(zeros)
stratas <- matrix( nrow=length(surv_fit.df$strata),ncol=2, unlist(strsplit(as.character(surv_fit.df$strata),", ")), byrow=T )
surv_fit.df$Plant <- as.factor(matrix(nrow=length(surv_fit.df$strata), ncol=2,unlist(strsplit(stratas[,2],"=")), byrow=T )[,2])
surv_fit.df$Strain <- as.factor(matrix(nrow=length(surv_fit.df$strata), ncol=2,unlist(strsplit(stratas[,1],"=")), byrow=T )[,2])

###End of data frame generation
library("ggplot2")
###Start plotting
ggplot(surv_fit.df,aes(time, surv, colour = Strain)) + 
  facet_grid(Strain~Plant) +
  stat_summary(aes(y = surv), fun.y=mean, geom="step") +
  ggtitle("All KM survival estimates")
```

## Pairwise logrank testing

If one is interested between all pairwise differences of ALL StrainPlant combinations (6216 pairs), run the below. This will perform a logrank test for every possible combination of strain and plant and test against all others. This is stratified by ROTOPLAN. This is not evaluated in the rmarkdown file because the output is huge and not particularily helpful. The below does not correct for multiple comparisons.

```
###Pairwise--- http://stackoverflow.com/questions/17338774/r-formula-how-to-constrain-calculations-to-two-groups-using-formula
library("gtools")

levels<-(unique(surv_table$StrainPlant))

groups<-combinations(length(levels),2,levels)

pairWise_StrainPlant <- plyr::alply(groups, 1, 
    function(pair) {
        survdiff(Surv(DPI, as.numeric(Diseased), type="right")~StrainPlant  + frailty(ROTOPLAN), surv_table , subset=surv_table$StrainPlant %in% c(pair[[1]], pair[[2]]) )
    })
names(pairWise_StrainPlant) <- plyr::alply(groups, 1, 
    function(pair) {
        c(pair[[1]], pair[[2]]) 
    })
```

## Hazards

```
library("survcomp")
```

```
## Loading required package: prodlim
```

```
###Cox-Proportional hazards####
#Build model
srv_coxph <- coxph(Surv(DPI, as.numeric(Diseased),type="right") ~Strain+Plant+ROTOPLAN, data=surv_table) 
###Check porportionality of hazards
cox.zph(srv_coxph, transform = "log")
```

```
## Warning: contrasts dropped from factor Strain
```

```
## Warning: contrasts dropped from factor Plant
```

```
## Warning: contrasts dropped from factor ROTOPLAN
```

```
##                 rho    chisq        p
## StrainRS10 -0.19033 3.72e+02 0.00e+00
## StrainRS11 -0.17451 3.12e+02 0.00e+00
## StrainRS12 -0.06034 3.73e+01 1.04e-09
## StrainRS13 -0.01777 3.23e+00 7.21e-02
## StrainRS14  0.00438 1.98e-01 6.57e-01
## StrainRS2   0.00809 6.71e-01 4.13e-01
## StrainRS3   0.01729 3.06e+00 8.03e-02
## StrainRS4   0.02189 4.91e+00 2.67e-02
## StrainRS5   0.01829 3.43e+00 6.41e-02
## StrainRS6  -0.01722 3.04e+00 8.12e-02
## StrainRS7   0.01656 2.81e+00 9.37e-02
## StrainRS8  -0.00281 8.09e-02 7.76e-01
## StrainRS9  -0.01052 1.13e+00 2.87e-01
## PlantB      0.01789 3.28e+00 7.01e-02
## PlantC     -0.01067 1.17e+00 2.80e-01
## PlantD     -0.00924 8.75e-01 3.50e-01
## PlantE     -0.00789 6.41e-01 4.23e-01
## PlantF     -0.01989 4.05e+00 4.42e-02
## PlantG     -0.01084 1.20e+00 2.73e-01
## PlantH     -0.04584 2.15e+01 3.50e-06
## ROTOPLAN1  -0.05891 3.63e+01 1.72e-09
## ROTOPLAN2   0.01473 2.23e+00 1.35e-01
## ROTOPLAN3   0.01926 3.84e+00 5.01e-02
## ROTOPLAN4  -0.06416 4.28e+01 6.07e-11
## GLOBAL           NA 1.78e+03 0.00e+00
```

```
###Hazard ration analysis, not appropriate if cox.zph above returns a low pvalue
hazrat <- hazard.ratio(surv_table$Strain, surv_table$DPI, surv.event = as.numeric(surv_table$Diseased))
```

The proportional hazards assumption is violated.

## Survival Regression

As the hazards are non-proportional, and generating a ton of pairwise tests as done above without correcting for multiple comparisons is a really bad idea, survival regression could be handy.

```
library("rms")
```

```
psm_gaus <- psm(Surv(DPI, as.numeric(Diseased), type="right") ~ Strain * Plant + ROTOPLAN, surv_table, dist = "gaussian")
psm_logistic <- psm(Surv(DPI, as.numeric(Diseased), type="right") ~ Strain * Plant + ROTOPLAN, surv_table, dist = "logistic")
psm_lnorm <- psm(Surv(DPI, as.numeric(Diseased), type="right") ~ Strain * Plant + ROTOPLAN, surv_table, dist = "lognormal")
psm_wei <- psm(Surv(DPI, as.numeric(Diseased), type="right") ~ Strain * Plant + ROTOPLAN, surv_table, dist = "weibull")
```

```
## Warning in survreg.fit(X, Y, weights, offset, init = init, controlvals =
## control, : Ran out of iterations and did not converge
```

```
aic.scores.psm <- rbind(
  extractAIC(psm_wei),
  extractAIC(psm_gaus),
  extractAIC(psm_logistic),
  extractAIC(psm_lnorm))
###Make useable AIC table
rownames(aic.scores.psm) <- c("Weibull", "Gaussian", "Logist", "Lognorm")
colnames(aic.scores.psm) <- c("df", "AIC")
aic.scores.psm
```

```
##           df       AIC
## Weibull  117 113645.10
## Gaussian 117  79600.29
## Logist   117  80607.59
## Lognorm  117  80392.17
```

Gaussian is the best fit. Explore the gaussian psm

```
library("multcomp")
```

```
## Loading required package: mvtnorm
```

```
## Loading required package: TH.data
```

```
## Loading required package: MASS
```

```
## 
## Attaching package: 'TH.data'
```

```
## The following object is masked from 'package:MASS':
## 
##     geyser
```

```
summary(glht(psm_gaus, linfct=mcp(Strain="Tukey")))
```

```
## Warning in mcp2matrix(model, linfct = linfct): covariate interactions found
## -- default contrast might be inappropriate
```

```
## Warning in RET$pfunction("adjusted", ...): Completion with error > abseps

## Warning in RET$pfunction("adjusted", ...): Completion with error > abseps

## Warning in RET$pfunction("adjusted", ...): Completion with error > abseps

## Warning in RET$pfunction("adjusted", ...): Completion with error > abseps

## Warning in RET$pfunction("adjusted", ...): Completion with error > abseps

## Warning in RET$pfunction("adjusted", ...): Completion with error > abseps

## Warning in RET$pfunction("adjusted", ...): Completion with error > abseps

## Warning in RET$pfunction("adjusted", ...): Completion with error > abseps

## Warning in RET$pfunction("adjusted", ...): Completion with error > abseps

## Warning in RET$pfunction("adjusted", ...): Completion with error > abseps

## Warning in RET$pfunction("adjusted", ...): Completion with error > abseps

## Warning in RET$pfunction("adjusted", ...): Completion with error > abseps

## Warning in RET$pfunction("adjusted", ...): Completion with error > abseps

## Warning in RET$pfunction("adjusted", ...): Completion with error > abseps
```

```
## 
##   Simultaneous Tests for General Linear Hypotheses
## 
## Multiple Comparisons of Means: Tukey Contrasts
## 
## 
## Fit: psm(formula = Surv(DPI, as.numeric(Diseased), type = "right") ~ 
##     Strain * Plant + ROTOPLAN, data = surv_table, dist = "gaussian")
## 
## Linear Hypotheses:
##                   Estimate Std. Error z value Pr(>|z|)    
## RS10 - RS1 == 0  -10.95176    1.04690 -10.461    <0.01 ***
## RS11 - RS1 == 0   -8.71967    1.07746  -8.093    <0.01 ***
## RS12 - RS1 == 0   -1.14039    1.21223  -0.941   0.9996    
## RS13 - RS1 == 0    2.94921    1.34993   2.185   0.6354    
## RS14 - RS1 == 0   -2.49739    1.20444  -2.073   0.7141    
## RS2 - RS1 == 0    -0.65822    1.21126  -0.543   1.0000    
## RS3 - RS1 == 0     0.67626    1.24148   0.545   1.0000    
## RS4 - RS1 == 0    -0.01345    1.21878  -0.011   1.0000    
## RS5 - RS1 == 0    -0.26534    1.21387  -0.219   1.0000    
## RS6 - RS1 == 0    -1.17259    1.18939  -0.986   0.9994    
## RS7 - RS1 == 0    -0.27662    1.21800  -0.227   1.0000    
## RS8 - RS1 == 0    -0.41951    1.21248  -0.346   1.0000    
## RS9 - RS1 == 0    -1.72957    1.18244  -1.463   0.9731    
## RS11 - RS10 == 0   2.23208    0.86330   2.586   0.3443    
## RS12 - RS10 == 0   9.81137    1.02700   9.553    <0.01 ***
## RS13 - RS10 == 0  13.90097    1.18639  11.717    <0.01 ***
## RS14 - RS10 == 0   8.45436    1.01736   8.310    <0.01 ***
## RS2 - RS10 == 0   10.29354    1.02573  10.035    <0.01 ***
## RS3 - RS10 == 0   11.62801    1.06140  10.955    <0.01 ***
## RS4 - RS10 == 0   10.93830    1.03459  10.573    <0.01 ***
## RS5 - RS10 == 0   10.68642    1.02863  10.389    <0.01 ***
## RS6 - RS10 == 0    9.77917    0.99965   9.783    <0.01 ***
## RS7 - RS10 == 0   10.67514    1.03361  10.328    <0.01 ***
## RS8 - RS10 == 0   10.53225    1.02710  10.254    <0.01 ***
## RS9 - RS10 == 0    9.22219    0.99143   9.302    <0.01 ***
## RS12 - RS11 == 0   7.57929    1.05813   7.163    <0.01 ***
## RS13 - RS11 == 0  11.66888    1.21347   9.616    <0.01 ***
## RS14 - RS11 == 0   6.22228    1.04907   5.931    <0.01 ***
## RS2 - RS11 == 0    8.06146    1.05708   7.626    <0.01 ***
## RS3 - RS11 == 0    9.39593    1.09150   8.608    <0.01 ***
## RS4 - RS11 == 0    8.70622    1.06550   8.171    <0.01 ***
## RS5 - RS11 == 0    8.45433    1.05977   7.977    <0.01 ***
## RS6 - RS11 == 0    7.54709    1.03170   7.315    <0.01 ***
## RS7 - RS11 == 0    8.44305    1.06461   7.931    <0.01 ***
## RS8 - RS11 == 0    8.30016    1.05831   7.843    <0.01 ***
## RS9 - RS11 == 0    6.99010    1.02371   6.828    <0.01 ***
## RS13 - RS12 == 0   4.08959    1.33441   3.065   0.1140    
## RS14 - RS12 == 0  -1.35701    1.18709  -1.143   0.9973    
## RS2 - RS12 == 0    0.48217    1.19424   0.404   1.0000    
## RS3 - RS12 == 0    1.81664    1.22485   1.483   0.9700    
## RS4 - RS12 == 0    1.12693    1.20173   0.938   0.9997    
## RS5 - RS12 == 0    0.87505    1.19676   0.731   1.0000    
## RS6 - RS12 == 0   -0.03220    1.17184  -0.027   1.0000    
## RS7 - RS12 == 0    0.86376    1.20082   0.719   1.0000    
## RS8 - RS12 == 0    0.72088    1.19543   0.603   1.0000    
## RS9 - RS12 == 0   -0.58918    1.16495  -0.506   1.0000    
## RS14 - RS13 == 0  -5.44660    1.32740  -4.103    <0.01 ** 
## RS2 - RS13 == 0   -3.60742    1.33362  -2.705   0.2719    
## RS3 - RS13 == 0   -2.27295    1.36115  -1.670   0.9246    
## RS4 - RS13 == 0   -2.96266    1.34046  -2.210   0.6160    
## RS5 - RS13 == 0   -3.21455    1.33589  -2.406   0.4697    
## RS6 - RS13 == 0   -4.12180    1.31368  -3.138   0.0942 .  
## RS7 - RS13 == 0   -3.22583    1.33973  -2.408   0.4681    
## RS8 - RS13 == 0   -3.36872    1.33468  -2.524   0.3848    
## RS9 - RS13 == 0   -4.67878    1.30755  -3.578   0.0234 *  
## RS2 - RS14 == 0    1.83918    1.18609   1.551   0.9569    
## RS3 - RS14 == 0    3.17365    1.21712   2.608   0.3306    
## RS4 - RS14 == 0    2.48394    1.19384   2.081   0.7104    
## RS5 - RS14 == 0    2.23206    1.18872   1.878   0.8352    
## RS6 - RS14 == 0    1.32481    1.16368   1.138   0.9974    
## RS7 - RS14 == 0    2.22077    1.19292   1.862   0.8437    
## RS8 - RS14 == 0    2.07788    1.18733   1.750   0.8955    
## RS9 - RS14 == 0    0.76782    1.15668   0.664   1.0000    
## RS3 - RS2 == 0     1.33447    1.22377   1.090   0.9983    
## RS4 - RS2 == 0     0.64476    1.20076   0.537   1.0000    
## RS5 - RS2 == 0     0.39288    1.19570   0.329   1.0000    
## RS6 - RS2 == 0    -0.51437    1.17086  -0.439   1.0000    
## RS7 - RS2 == 0     0.38160    1.19995   0.318   1.0000    
## RS8 - RS2 == 0     0.23871    1.19423   0.200   1.0000    
## RS9 - RS2 == 0    -1.07135    1.16380  -0.921   0.9997    
## RS4 - RS3 == 0    -0.68971    1.23118  -0.560   1.0000    
## RS5 - RS3 == 0    -0.94159    1.22632  -0.768   1.0000    
## RS6 - RS3 == 0    -1.84884    1.20215  -1.538   0.9597    
## RS7 - RS3 == 0    -0.95288    1.23055  -0.774   1.0000    
## RS8 - RS3 == 0    -1.09577    1.22498  -0.895   0.9998    
## RS9 - RS3 == 0    -2.40583    1.19523  -2.013   0.7555    
## RS5 - RS4 == 0    -0.25188    1.20329  -0.209   1.0000    
## RS6 - RS4 == 0    -1.15913    1.17862  -0.983   0.9994    
## RS7 - RS4 == 0    -0.26317    1.20755  -0.218   1.0000    
## RS8 - RS4 == 0    -0.40606    1.20195  -0.338   1.0000    
## RS9 - RS4 == 0    -1.71612    1.17162  -1.465   0.9728    
## RS6 - RS5 == 0    -0.90725    1.17341  -0.773   1.0000    
## RS7 - RS5 == 0    -0.01128    1.20250  -0.009   1.0000    
## RS8 - RS5 == 0    -0.15417    1.19684  -0.129   1.0000    
## RS9 - RS5 == 0    -1.46423    1.16644  -1.255   0.9931    
## RS7 - RS6 == 0     0.89597    1.17776   0.761   1.0000    
## RS8 - RS6 == 0     0.75308    1.17204   0.643   1.0000    
## RS9 - RS6 == 0    -0.55698    1.14099  -0.488   1.0000    
## RS8 - RS7 == 0    -0.14289    1.20111  -0.119   1.0000    
## RS9 - RS7 == 0    -1.45295    1.17084  -1.241   0.9938    
## RS9 - RS8 == 0    -1.31006    1.16504  -1.124   0.9977    
## ---
## Signif. codes:  0 '***' 0.001 '**' 0.01 '*' 0.05 '.' 0.1 ' ' 1
## (Adjusted p values reported -- single-step method)
```

```
summary(glht(psm_gaus, linfct=mcp(Plant="Tukey")))
```

```
## Warning in mcp2matrix(model, linfct = linfct): covariate interactions found
## -- default contrast might be inappropriate

## Warning in mcp2matrix(model, linfct = linfct): Completion with error >
## abseps

## Warning in mcp2matrix(model, linfct = linfct): Completion with error >
## abseps

## Warning in mcp2matrix(model, linfct = linfct): Completion with error >
## abseps

## Warning in mcp2matrix(model, linfct = linfct): Completion with error >
## abseps

## Warning in mcp2matrix(model, linfct = linfct): Completion with error >
## abseps
```

```
## 
##   Simultaneous Tests for General Linear Hypotheses
## 
## Multiple Comparisons of Means: Tukey Contrasts
## 
## 
## Fit: psm(formula = Surv(DPI, as.numeric(Diseased), type = "right") ~ 
##     Strain * Plant + ROTOPLAN, data = surv_table, dist = "gaussian")
## 
## Linear Hypotheses:
##            Estimate Std. Error z value Pr(>|z|)    
## B - A == 0  -0.7901     1.2049  -0.656  0.99801    
## C - A == 0  -2.4904     1.1628  -2.142  0.38577    
## D - A == 0  -3.7013     1.1459  -3.230  0.02680 *  
## E - A == 0   1.6412     1.2791   1.283  0.90451    
## F - A == 0   0.6841     1.2635   0.541  0.99942    
## G - A == 0   2.3893     1.3163   1.815  0.60753    
## H - A == 0  -2.9529     1.1568  -2.553  0.17143    
## C - B == 0  -1.7004     1.1371  -1.495  0.80895    
## D - B == 0  -2.9112     1.1198  -2.600  0.15461    
## E - B == 0   2.4313     1.2559   1.936  0.52384    
## F - B == 0   1.4741     1.2399   1.189  0.93467    
## G - B == 0   3.1793     1.2937   2.458  0.21194    
## H - B == 0  -2.1628     1.1309  -1.912  0.54007    
## D - C == 0  -1.2109     1.0743  -1.127  0.95058    
## E - C == 0   4.1316     1.2155   3.399  0.01545 *  
## F - C == 0   3.1745     1.1991   2.647  0.13757    
## G - C == 0   4.8797     1.2547   3.889  0.00240 ** 
## H - C == 0  -0.4625     1.0859  -0.426  0.99988    
## E - D == 0   5.3425     1.1993   4.455  < 0.001 ***
## F - D == 0   4.3854     1.1828   3.708  0.00497 ** 
## G - D == 0   6.0905     1.2389   4.916  < 0.001 ***
## H - D == 0   0.7484     1.0678   0.701  0.99695    
## F - E == 0  -0.9571     1.3123  -0.729  0.99609    
## G - E == 0   0.7481     1.3630   0.549  0.99937    
## H - E == 0  -4.5941     1.2097  -3.798  0.00353 ** 
## G - F == 0   1.7052     1.3483   1.265  0.91109    
## H - F == 0  -3.6370     1.1933  -3.048  0.04682 *  
## H - G == 0  -5.3422     1.2491  -4.277  < 0.001 ***
## ---
## Signif. codes:  0 '***' 0.001 '**' 0.01 '*' 0.05 '.' 0.1 ' ' 1
## (Adjusted p values reported -- single-step method)
```

## By plant genotype analysis

Maybe, it would be interesting to only know which strain performed how within each Plant genotype used.

```
options(warn = -1)
plants_array <- array(levels(surv_table$Plant))
plant_within <- sapply(plants_array, 
                    function(plant) {
                      psm(Surv(DPI, as.numeric(Diseased), type="right") ~ Strain + ROTOPLAN, data=surv_table[surv_table$Plant==plant,], dist="gaussian")
                    }, simplify=F)
names(plant_within) <- levels(surv_table$Plant)

plant_within_cld <- sapply(plants_array, 
                    function(plant) {
                      cld( glht( 
                        psm(Surv(DPI, as.numeric(Diseased), type="right") ~ Strain + ROTOPLAN, data=surv_table[surv_table$Plant==plant,], dist="gaussian"),
                        linfct=mcp(Strain="Tukey")
                      )
                      )
                    }
                    , simplify=F)
names(plant_within_cld) <- levels(surv_table$Plant)

plant_within_cld
```

```
## $A
##  RS1 RS10 RS11 RS12 RS13 RS14  RS2  RS3  RS4  RS5  RS6  RS7  RS8  RS9 
## "ab"  "c"  "c" "ab"  "a"  "b" "ab" "ab" "ab" "ab" "ab" "ab" "ab"  "b" 
## 
## $B
##  RS1 RS10 RS11 RS12 RS13 RS14  RS2  RS3  RS4  RS5  RS6  RS7  RS8  RS9 
## "ab"  "c"  "c" "ab" "ab" "ab" "ab"  "a" "ab" "ab" "ab" "ab" "ab"  "b" 
## 
## $C
##  RS1 RS10 RS11 RS12 RS13 RS14  RS2  RS3  RS4  RS5  RS6  RS7  RS8  RS9 
## "bc"  "e"  "e"  "c" "ab"  "d" "ab" "ab" "ab" "ab" "bc"  "a" "bc" "bc" 
## 
## $D
##   RS1  RS10  RS11  RS12  RS13  RS14   RS2   RS3   RS4   RS5   RS6   RS7 
##  "ad"   "f"   "e" "acd"   "b"  "ab"  "bc"  "ab"   "b"  "ab"  "ae"  "ab" 
##   RS8   RS9 
##  "bd"  "ab" 
## 
## $E
##   RS1  RS10  RS11  RS12  RS13  RS14   RS2   RS3   RS4   RS5   RS6   RS7 
##  "bc"   "d"   "d"  "ab"  "ab" "bcd"  "bc"  "bc"   "c"  "bc"  "ab"  "ac" 
##   RS8   RS9 
##  "bc"   "b" 
## 
## $F
##   RS1  RS10  RS11  RS12  RS13  RS14   RS2   RS3   RS4   RS5   RS6   RS7 
##  "ac"   "f"   "f"  "cd" "bce"  "ab"  "ae"  "ac"   "a" "abd"  "bc"  "ac" 
##   RS8   RS9 
##  "ac"   "c" 
## 
## $G
##   RS1  RS10  RS11  RS12  RS13  RS14   RS2   RS3   RS4   RS5   RS6   RS7 
##  "bc"   "a"   "a"  "cd"   "b" "abc"  "bd"  "bc"  "bc"  "bc"  "bc"  "cd" 
##   RS8   RS9 
##  "bd"   "c" 
## 
## $H
##   RS1  RS10  RS11  RS12  RS13  RS14   RS2   RS3   RS4   RS5   RS6   RS7 
##  "be"   "f"   "f"   "b"   "b"   "a" "cde"   "c"   "c"  "ce"  "bc"  "ce" 
##   RS8   RS9 
## "cde"  "bd"
```

## By strain analysis

```
strain_array <- array(levels(surv_table$Strain))
strain_within <- sapply(strain_array, 
                    function(strain) {
                      psm(Surv(DPI, as.numeric(Diseased), type="right") ~ Plant + ROTOPLAN, data=surv_table[surv_table$Strain==strain,], dist="gaussian")
                    }, simplify=F)
names(strain_within) <- levels(surv_table$Strain)

###Strain4 / Rs14, can not be fit. This is probably because it is flatlining (not causing symptoms) for some plants. This strain is excluded.
###I would consider this a relevant finding, but it is also quite obvious from all analysis in this file that this strain is different from the others

strain_within_cld <- sapply(strain_array[c(1:5,7:length(strain_array))], 
                    function(strain) {
                      cld( glht( 
                        psm(Surv(DPI, as.numeric(Diseased), type="right") ~ Plant + ROTOPLAN, data=surv_table[surv_table$Strain==strain,], dist="gaussian"),
                        linfct=mcp(Plant="Tukey")
                      )
                      )
                    }
                    , simplify=F)
names(strain_within_cld) <- levels(surv_table$Strain)[ c(1:5,7:length(strain_array)) ]

strain_within_cld
```

```
## $RS1
##     A     B     C     D     E     F     G     H 
##  "ac"  "ad" "bcd"   "d"   "a"  "ab"   "a"  "cd" 
## 
## $RS10
##    A    B    C    D    E    F    G    H 
## "bc" "ac" "ac"  "c"  "a" "ab" "ab"  "c" 
## 
## $RS11
##    A    B    C    D    E    F    G    H 
## "ab" "ab" "ab"  "a" "ab"  "b" "ab"  "c" 
## 
## $RS12
##    A    B    C    D    E    F    G    H 
## "ac" "ac"  "b" "ab"  "c" "bc"  "c"  "b" 
## 
## $RS13
##    A    B    C    D    E    F    G    H 
## "ab" "bc" "bc" "bc" "bc"  "c"  "a"  "d" 
## 
## $RS2
##    A    B    C    D    E    F    G    H 
## "bc"  "c" "ac" "ac" "ab" "ab"  "a" "ac" 
## 
## $RS3
##    A    B    C    D    E    F    G    H 
## "ab" "ab" "ab"  "a"  "b" "ab" "ab" "ab" 
## 
## $RS4
##    A    B    C    D    E    F    G    H 
## "bc"  "c" "bc" "bc"  "a" "ab" "ab" "ac" 
## 
## $RS5
##    A    B    C    D    E    F    G    H 
## "bc"  "b" "ab"  "b"  "a" "ab" "ac" "ab" 
## 
## $RS6
##    A    B    C    D    E    F    G    H 
## "ab" "ab" "bc"  "c" "ab" "ac"  "b" "bc" 
## 
## $RS7
##    A    B    C    D    E    F    G    H 
## "ab" "ab" "ac"  "b"  "c" "ab" "bc" "bc" 
## 
## $RS8
##    A    B    C    D    E    F    G    H 
## "bc"  "c"  "c" "bc" "ab" "ac"  "a"  "c" 
## 
## $RS9
##   A   B   C   D   E   F   G   H 
## "a" "a" "a" "a" "a" "a" "a" "a"
```

```
contrasts(surv_table$Strain)
```

```
##      RS10 RS11 RS12 RS13 RS14 RS2 RS3 RS4 RS5 RS6 RS7 RS8 RS9
## RS1     0    0    0    0    0   0   0   0   0   0   0   0   0
## RS10    1    0    0    0    0   0   0   0   0   0   0   0   0
## RS11    0    1    0    0    0   0   0   0   0   0   0   0   0
## RS12    0    0    1    0    0   0   0   0   0   0   0   0   0
## RS13    0    0    0    1    0   0   0   0   0   0   0   0   0
## RS14    0    0    0    0    1   0   0   0   0   0   0   0   0
## RS2     0    0    0    0    0   1   0   0   0   0   0   0   0
## RS3     0    0    0    0    0   0   1   0   0   0   0   0   0
## RS4     0    0    0    0    0   0   0   1   0   0   0   0   0
## RS5     0    0    0    0    0   0   0   0   1   0   0   0   0
## RS6     0    0    0    0    0   0   0   0   0   1   0   0   0
## RS7     0    0    0    0    0   0   0   0   0   0   1   0   0
## RS8     0    0    0    0    0   0   0   0   0   0   0   1   0
## RS9     0    0    0    0    0   0   0   0   0   0   0   0   1
```

Finally, check for a rotoplan effect across the full dataset, using the same distribution as earlier

## Rotoplan effect?

```
cld(glht(
  survreg(Surv(DPI, as.numeric(Diseased), type="right") ~ Strain + ROTOPLAN, data=surv_table, dist="gaussian"),
                        linfct=mcp(ROTOPLAN="Tukey")
))
```

```
##   1   2   3   4   5 
## "c" "b" "b" "c" "a"
```

As can be seen, ROTOPLAN has a signficant effect. Rotoplans 2 and 3 are in the same signficance group, meaning they are not significantly different. The same is true for 1 and 4. One could explore this further by subsetting the dataset into two groups, one made of ROTOPLANs (1,4) and one of (2,3) and analyze those individually.

## Plotting survival regression

```
library("stringr")
library("dplyr")
```

```
## 
## Attaching package: 'dplyr'
```

```
## The following object is masked from 'package:MASS':
## 
##     select
```

```
## The following objects are masked from 'package:Hmisc':
## 
##     combine, src, summarize
```

```
## The following objects are masked from 'package:stats':
## 
##     filter, lag
```

```
## The following objects are masked from 'package:base':
## 
##     intersect, setdiff, setequal, union
```

```
###The StrainPlant variable was generated earlier.
###The below assumes interactions between Strain and Plant, meaning we could write
### Surv(DPI, as.numeric(Diseased), type="right") ~ Strain * Plant
###and obtain the same results. I prefer the way it is currently done, because i can split it back into the original things more conveniently.
s_reg_gaus <- survreg(Surv(DPI, as.numeric(Diseased), type="right") ~ StrainPlant, surv_table, dist="gaussian")
s_reg_logistic <- survreg(Surv(DPI, as.numeric(Diseased), type="right") ~ StrainPlant, surv_table, dist="logistic")
s_reg_lnorm <- survreg(Surv(DPI, as.numeric(Diseased), type="right") ~ StrainPlant, surv_table, dist="lognormal")
s_reg_wei <- survreg(Surv(DPI, as.numeric(Diseased), type="right") ~ StrainPlant, surv_table, dist="weibull")

###Step 1, extract the coefficients. These are relative to StrainPlant combination RS1.A because StrainPlant is treatment contrasted.
surv_fit.df$StrainPlant <- interaction(surv_fit.df$Strain,surv_fit.df$Plant)
for (i in 1:nlevels(surv_fit.df$StrainPlant)) { #For loop through strains
  if(i==1) { #Strain1 is relative to itself, so no change
  coef_wei <- list()
  coef_logistic <- list()
  coef_gaus <- list()
  coef_lnorm <- list()
  coef_wei[i] <- coef(s_reg_wei)[i]
  coef_logistic[i] <- coef(s_reg_logistic)[i]
  coef_gaus[i] <- coef(s_reg_gaus)[i]
  coef_lnorm[i] <- coef(s_reg_lnorm)[i]
  } else { ###Other strains are relative to 1
  coef_wei[i] <- coef(s_reg_wei)[1] + coef(s_reg_wei)[i]
  coef_logistic[i] <- coef(s_reg_logistic)[1] + coef(s_reg_logistic)[i]
  coef_gaus[i] <- coef(s_reg_gaus)[1] + coef(s_reg_gaus)[i]
  coef_lnorm[i] <- coef(s_reg_lnorm)[1] + coef(s_reg_lnorm)[i]
  }
}
##Step 2
####Store the coefficients and the scale in a new data frame, of parameters
### Keep in mind that survreg.distributions$weibull is different from rweibull, hence the difference in names.
sregparams <- data.frame(
  StrainPlant = rep(levels(surv_table$StrainPlant),4 ), #Fill with strains
  scale.wei = exp(unlist(coef_wei)), #weibull fit scale parameters
  scale.logistic = rep(s_reg_logistic$scale, nlevels(surv_table$StrainPlant)), #fill with logis scales
  scale.gaus = rep(s_reg_gaus$scale, nlevels(surv_table$StrainPlant)), #fill with gaus scales
  scale.lnorm = rep(s_reg_lnorm$scale, nlevels(surv_table$StrainPlant)), #fill with lnorm scale
  shape.wei =  rep(1/s_reg_wei$scale, nlevels(surv_table$StrainPlant)), #shape for weibull
  shape.logistic = unlist(coef_logistic), #shape for logistic
  shape.gaus =  unlist(coef_gaus), #shape for gaus
  shape.lnorm =   unlist(coef_lnorm) #shape for lnorm
  )
##Step 3
###Calculate the "daily" value of each curve
for (i in 1:nlevels(surv_fit.df$StrainPlant)){
  if(i==1) {
    wei <- list()
    logis <- list()
    gaus <- list()
    lnorm <- list()
  }
  x <- levels(surv_fit.df$StrainPlant)[i]
  n <- c(1:max(surv_table$DPI))
  data <- dplyr::filter(sregparams, StrainPlant==x) ###Watch out here. It is crucial to use dplyr::filter and not base::filter
  time <- n
  wei <- cbind(wei, pweibull(
    q=n,
    scale=data$scale.wei,
    shape=data$shape.wei,
    lower.tail=FALSE))
  logis <- cbind(logis,plogis(
    q=n,
    scale=data$scale.logistic,
    location=data$shape.logistic,
    lower.tail=FALSE  ))
  gaus <- cbind(gaus,pnorm(
   q=n,
   sd=data$scale.gaus,
   mean=data$shape.gaus,
   lower.tail = F))
  lnorm <- cbind(lnorm, plnorm(
    q=n,
    sd=data$scale.lnorm,
    mean=data$shape.lnorm,
    lower.tail=F))
}

##Step 4
###Put all the curves into a data.frame that contains information on "time" and also "Strain", for compatibility with other data.frames
sreg_curves <- data.frame(
  wei.sreg = cbind(unlist(wei)),
  logis.sreg = cbind(unlist(logis)),
  gaus.sreg = cbind(unlist(gaus)),
  lnorm.sreg = cbind(unlist(lnorm)),
  StrainPlant = rep(unlist(levels(surv_fit.df$StrainPlant)),each=max(surv_table$DPI)),
  time = rep(c(1:max(surv_table$DPI)), nlevels(surv_fit.df$StrainPlant))
)
sreg_curves$Strain <- str_split_fixed(sreg_curves$StrainPlant, "\\.",2)[,1]
sreg_curves$Plant <- str_split_fixed(sreg_curves$StrainPlant, "\\.",2)[,2]
##Step 5
###Turn that data.frame into a long data.frame (not used here but for other figures.)
sreg_long <- sreg_curves %>% gather(., key="Distribution",values = c(lnorm.sreg, wei.sreg,gaus.sreg,logis.sreg) )
sreg_long$Distribution <- as.factor(sreg_long$Distribution)
##Levels: gaus.sreg wei.sreg
levels(sreg_long$Distribution) <- c("Gaussian","Lognormal","Loglogistic","Weibull")

sreg_long$Strain <- str_split_fixed(sreg_long$StrainPlant, "\\.",2)[,1]
sreg_long$Plant <- str_split_fixed(sreg_long$StrainPlant, "\\.",2)[,2]
```

Now, these can be plotted and inspected visually..

```
###Plot of KM+Weibull
  ggplot(surv_fit.df, aes(time, surv, colour = Strain)) +
  geom_step() +
  geom_line(data=sreg_curves,aes(y=wei.sreg),color="black") +
  facet_grid(Plant~Strain)+ 
  ggtitle("Kaplan-Meier estimates and fit to\nWeibull distribution")
```

```
###Plot of KM+Gaussian  
  ggplot(surv_fit.df, aes(time, surv, colour = Strain)) +
    geom_step() +
    geom_line(data=sreg_curves,aes(y=gaus.sreg),color="black") +
    facet_grid(Plant~Strain) + 
    ggtitle("Kaplan-Meier estimates and fit to\nGaussian distribution")
```

```
##Logis
    ggplot(surv_fit.df, aes(time, surv, colour = Strain)) +
    geom_step() +
    geom_line(data=sreg_curves,aes(y=logis.sreg),color="black") +
    facet_grid(Plant~Strain) + 
    ggtitle("Kaplan-Meier estimates and fit to\nLogistic distribution")
```

```
  ##Lnorm
    ggplot(surv_fit.df, aes(time, surv, colour = Strain)) +
    geom_step() +
    geom_line(data=sreg_curves,aes(y=lnorm.sreg),color="black") +
    facet_grid(Plant~Strain) + 
    ggtitle("Kaplan-Meier estimates and fit to\nLognormal distribution")
```

# Session Info

```
sessionInfo()
```

```
## R version 3.3.2 (2016-10-31)
## Platform: x86_64-apple-darwin13.4.0 (64-bit)
## Running under: macOS Sierra 10.12.1
## 
## locale:
## [1] en_US.UTF-8/en_US.UTF-8/en_US.UTF-8/C/en_US.UTF-8/en_US.UTF-8
## 
## attached base packages:
## [1] stats     graphics  grDevices utils     datasets  methods   base     
## 
## other attached packages:
##  [1] dplyr_0.5.0     stringr_1.1.0   multcomp_1.4-6  TH.data_1.0-7  
##  [5] MASS_7.3-45     mvtnorm_1.0-5   rms_5.1-0       SparseM_1.74   
##  [9] Hmisc_4.0-2     Formula_1.2-1   lattice_0.20-34 survcomp_1.24.0
## [13] prodlim_1.5.7   ggplot2_2.2.1   survival_2.40-1 tidyr_0.6.0    
## 
## loaded via a namespace (and not attached):
##  [1] zoo_1.7-14          reshape2_1.4.2      splines_3.3.2      
##  [4] colorspace_1.3-2    htmltools_0.3.5     yaml_2.1.14        
##  [7] base64enc_0.1-3     foreign_0.8-67      DBI_0.5-1          
## [10] RColorBrewer_1.1-2  plyr_1.8.4          lava_1.4.6         
## [13] MatrixModels_0.4-1  munsell_0.4.3       survivalROC_1.0.3  
## [16] gtable_0.2.0        codetools_0.2-15    evaluate_0.10      
## [19] labeling_0.3        latticeExtra_0.6-28 knitr_1.15.1       
## [22] quantreg_5.29       htmlTable_1.8       Rcpp_0.12.8        
## [25] acepack_1.4.1       KernSmooth_2.23-15  scales_0.4.1       
## [28] backports_1.0.4     checkmate_1.8.2     rmeta_2.16         
## [31] bootstrap_2015.2    gridExtra_2.2.1     digest_0.6.11      
## [34] polspline_1.1.12    stringi_1.1.2       SuppDists_1.1-9.4  
## [37] grid_3.3.2          rprojroot_1.1       tools_3.3.2        
## [40] sandwich_2.3-4      magrittr_1.5        lazyeval_0.2.0     
## [43] tibble_1.2          cluster_2.0.5       Matrix_1.2-7.1     
## [46] data.table_1.10.0   assertthat_0.1      rmarkdown_1.3      
## [49] R6_2.2.0            rpart_4.1-10        nlme_3.1-128       
## [52] nnet_7.3-12
```
